# ECCENTRIC: A fast and unrestrained approach for high-resolution in vivo metabolic imaging at ultra-high field MR

**DOI:** 10.1162/imag_a_00313

**Published:** 2024-10-14

**Authors:** Antoine Klauser, Bernhard Strasser, Wolfgang Bogner, Lukas Hingerl, Sebastien Courvoisier, Claudiu Schirda, Bruce R. Rosen, Francois Lazeyras, Ovidiu C. Andronesi

**Affiliations:** Athinoula A. Martinos Center for Biomedical Imaging, Department of Radiology, Massachusetts General Hospital, Harvard Medical School, Boston, MA, United States; Advanced Clinical Imaging Technology, Siemens Healthcare AG, Lausanne, Switzerland; Department of Radiology and Medical Informatics, University of Geneva, Geneva, Switzerland; CIBM Center for Biomedical Imaging, Geneva, Switzerland; High-Field MR Center, Department of Biomedical Imaging and Image-Guided Therapy, Medical University of Vienna, Vienna, Austria; Department of Radiology, University of Pittsburgh School of Medicine, Pittsburgh, PA, United States

**Keywords:** high resolution whole-brain metabolite imaging, 3D magnetic resonance spectroscopic imaging, ultra-high field, non-Cartesian, compressed sensing, low-rank

## Abstract

A novel method for fast and high-resolution metabolic imaging, called ECcentric Circle ENcoding TRajectorIes for Compressed sensing (ECCENTRIC), has been developed at 7 Tesla MRI. ECCENTRIC is a non-Cartesian spatial-spectral encoding method designed to accelerate magnetic resonance spectroscopic imaging (MRSI) with high signal-to-noise at ultra-high field. The approach provides flexible and random sampling of the Fourier space without temporal interleaving to improve spatial response function and spectral quality. ECCENTRIC enables the implementation of spatial-spectral MRSI with reduced gradient amplitudes and slew-rates, thereby mitigating electrical, mechanical, and thermal stress of the scanner hardware. Moreover, it exhibits robustness against timing imperfections and eddy-current delay. Combined with a model-based low-rank reconstruction, this approach enables simultaneous imaging of up to 14 metabolites over the whole brain at 2–3 mm isotropic resolution in 4–10 min. MRSI ECCENTRIC was performed on four healthy volunteers, yielding high-resolution spatial mappings of neurochemical profiles within the human brain. This innovative tool introduces a novel approach to neuroscience, providing new insights into the exploration of brain activity and physiology.

## 1 Introduction

Magnetic Resonance Spectroscopic Imaging (MRSI) is a well-established molecular MR imaging modality, facilitating non-invasive exploration of *in vivo* metabolism in both human and animal models without the use of ionizing radiation. In particular, ^1^H-MRSI can simultaneously image up to 20 brain metabolites, providing quantification of steady-state concentrations (Maudsley et al., 2020) and the dynamic change of concentrations under functional tasks (Bednarik et al., 2023; Mullins, 2018). In addition to measuring intrinsic metabolism without the need of contrast agents, MRSI can probe metabolic enzymatic rates that are not accessible by nuclear imaging techniques such as PET and SPECT (Davis et al., 2020). Many studies demonstrated significant value of MRSI for neuroscience (Oz et al., 2014), but the performance of current MRSI is severely lacking behind other MRI methods, which limits its use and wider adoption.

Among MRI modalities, MRSI is positioned to benefit the most from ultra-high field (UHF ≥ 7T) due to increased spectral dispersion and signal-to-noise ratio (SNR). MRSI using very short echo-time (≈1 ms) free induction decay (FID) excitation (Boer et al., 2012; Bogner et al., 2012; Henning et al., 2009) has great potential for metabolite imaging due to its high SNR. Nevertheless, MRSI is hampered by significant limitations, including low resolution and long scan times required for acquiring the 4D(k,t ) spatial-temporal space (Bogner et al., 2021). This highlights a pressing demand for acceleration strategies in high-resolution MRSI to overcome these challenges. This is especially pertinent in the context of high-resolution whole-brain MRSI, where conventional phase-encoding acquisition schemes would require scan time as long as several hours. Acceleration of UHF MRSI has been shown by parallel imaging such as SENSE, GRAPPA, and CAIPIRINHA with uniform undersampling (Hangel et al., 2018; Nassirpour, Chang, & Henning, 2018b; Strasser et al., 2017), or by Compressed Sensing (CS) with random undersampling (Nassirpour, Chang, Avdievitch, & Henning, 2018). However, similar to MRI, these techniques, as for MRI, generally do not allow acceleration factors (AF) above 6–10 for MRSI. Additionally, spatial-spectral encoding (SSE) techniques introduce additional prospects for accelerating UHF MRSI as demonstrated by Otazo et al. (2006). By combining spatial-spectral encoding with undersampling, higher accelerations (AF>50 ) of UHF MRSI can be achieved (Ma et al., 2016; Moser et al., 2019; Saucedo et al., 2021).

So far, SSE has been demonstrated at ultra-high fields using either Cartesian (echo-planar) (An et al., 2018; Nam et al., 2022; Weng et al., 2022) and non-Cartesian (spirals, rosettes, concentric circles) (Chiew et al., 2018; Esmaeili et al., 2021; Hingerl et al., 2020; Moser et al., 2019) k-space trajectories.

Nonetheless, integrating SSE at ultra-high field (UHF) presents formidable challenges stemming from the inverse relationship between the maximum time allocated for a trajectory repetition and the desired spectral bandwidth (SBW). This difficulty is accentuated at higher spatial resolutions in UHF, where trajectories must cover extensive k-space while maintaining even faster repetition rates for larger SBW at UHF. Consequently, this imposes significant technical demands on the gradient system in terms of amplitude and slew rate. The employment of temporal interleaving presents a viable strategy to overcome this constraint, facilitating the achievement of broad spectral bandwidth and high spatial resolution for circle or spiral trajectory (Adalsteinsson et al., 1998; Hingerl et al., 2018; Matsui et al., 1985). For echo-planar trajectory, achieving high SBW can be achieved by interlaced Fourier transform (Ebel et al., 2005) and interleaved readout gradients with alternating polarity (An et al., 2018; Posse et al., 2007). Nevertheless, these approaches concurrently prolong the acquisition duration and might introduce spectral side-bands that degrade the signal-to-noise ratio (SNR) and interfere with metabolite spectra. This issue arises if the number of temporal interleaves and the SBW are not optimally selected to prevent side-bands from appearing within the frequency range of interest (Bogner et al., 2021). Additionally, dedicated hardware, such as gradient inserts, allows circumvention of limitations on SBW (Versteeg et al., 2024).

In this study, we introduce ECCENTRIC method (ECcentric Circle ENcoding TRajectorIes for Compressed sensing) that benefits from: 1) improved pseudo-random sampling for CS with non-Cartesian trajectories, 2) flexible sampling of the 4D (k,t ) space for optimal SNR, 3) low-rank reconstruction for dimensionality reduction and denoising of data, and 4) reduced demand on the gradient system for spectral quality. Traditional trajectory designs, as mentioned above, often struggle to achieve high-resolution SSE MRSI at UHF without using temporal interleaving. To address this limitation, we designed the ECCENTRIC sampling pattern with smaller-sized circles. These circles, with their reduced radius, require lower gradient amplitude and slew rate compared to trajectories spanning the entire k-space, for identical spatial resolution and SBW. Given the upper limit in gradient amplitude and slew rate, ECCENTRIC is therefore advantageous as it allows for higher spatial resolution and/or SBW than established trajectories before reaching the gradient hardware limits, thereby avoiding the need for temporal interleaving.

The performance of the new acquisition-reconstruction scheme was first investigated by simulations and in a structural-metabolic phantom, and subsequently evaluated in vivo in healthy subjects.

## 2 Theory

Circular trajectories, including rosettes and concentric circles, provide several advantages over spiral and echo-planar trajectories in MRSI and MRI (Adalsteinsson et al., 1998; Furuyama et al., 2012; Posse et al., 1994; Schirda et al., 2018). By design, ECCENTRIC’s circular trajectories need smaller diameter than rosettes and concentric circles, which help achieve high-resolution and large spectral bandwidth at ultra-high field without temporal interleaving. ECCENTRIC trajectories are produced by readout gradient wave-forms that: 1) do not need rewinding which eliminates dead-time and the associated loss in SNR per unit time, 2) permit high matrix sizes with limited gradient amplitude, and 3) have constant and moderate gradient slew-rate that minimizes patient nerve stimulation and is not demanding for the gradient hardware. In contrast, MRSI SSE acquisitions are characterized by high gradient amplitudes and slew rates, which can exacerbate $B_{0}$ field drift (An et al., 2018) and induce eddy current artifacts (Xu et al., 2020) with echo-planar trajectories. Spiral SSE trajectories suffer from gradient imperfections and eddy currents, leading to trajectory distortions (Kim & Spielman, 2006). Similarly, concentric ring trajectories experience rotation errors due to timing delays and eddy currents, although to a lesser extent (Jiang et al., 2016). Additionally, the high demand on the gradient system often leads to temperature increases (Nam et al., 2023) and mechanical resonances (Dillinger et al., 2024).

This effect is particularly pronounced at 7T, where, for equivalent spatial resolution, the FID bandwidth is approximately doubled compared to 3T, necessitating a doubling of the gradient slew-rate for SSE encoding.

**Fig. 1.**
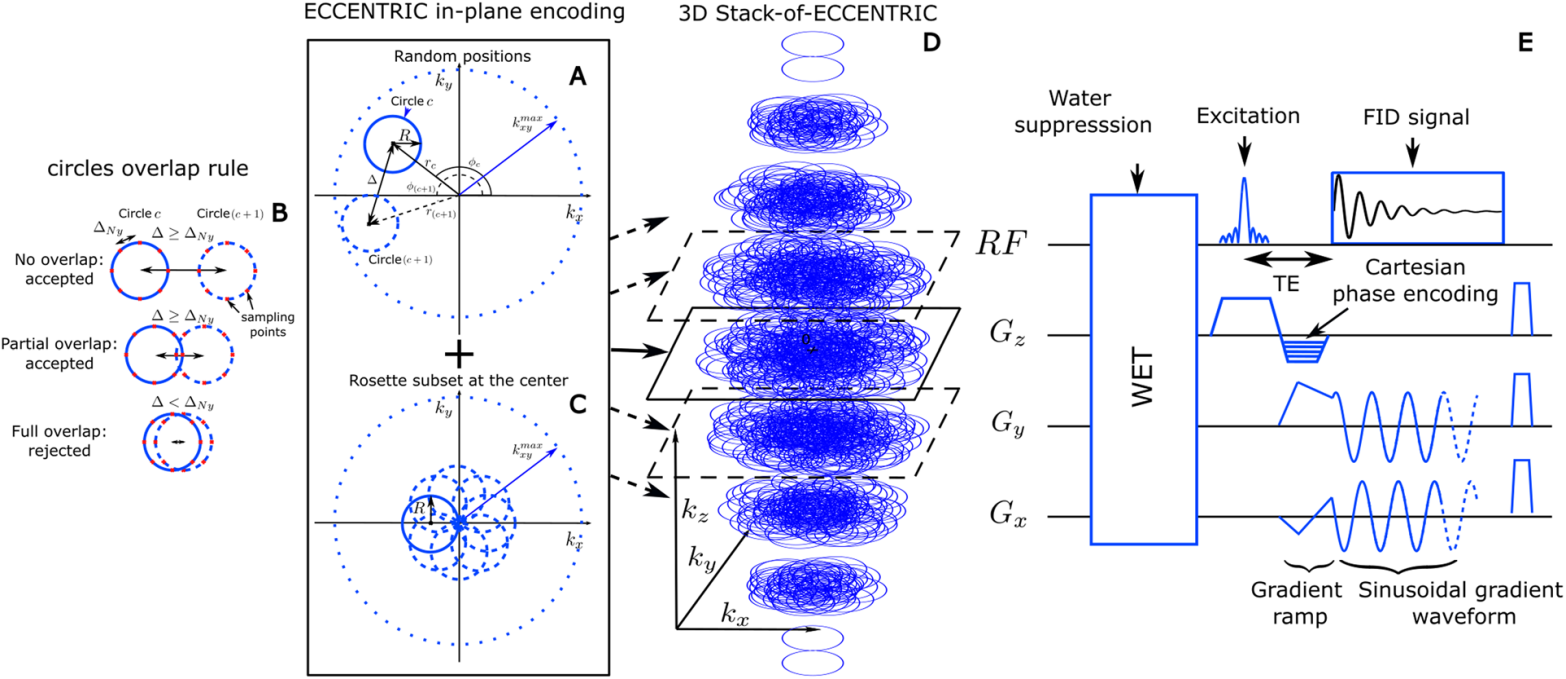
3D ECCENTRIC sampling and acquisition. (A) Circle center positions are parameterized in polar coordinates $\left(r_{c},\phi_{c}\right)$ that are chosen randomly in the ranges $r_{c}\in \left[0,\text{max}\left(k_{x,y}^{max}-r,r\right)\right]$ and $\phi_{c}\in \left[0,2\pi \right]$. Two consecutive circles (c and c+1 ) must respect the overlap rule described in (B): the distance between their respective centers, Δ, must be greater or equal to the Nyquist distance, $\Delta_{Ny}$ . (C), to satisfy a systematic full sampling of the k-space center, a small subset (<5% ) of circles is positioned in rosette pattern in each ECCENTRIC encoding planes. (D), 3D k-space sampling is achieved by a stack of ECCENTRIC encoding planes with variable $k_{x,y}^{max}$ to realize an ellipsoid coverage. (E) Diagram of the 3D ECCENTRIC FID-MRSI sequence. First, a 4-pulse WET water suppression technique is used, followed by the Shinnar–Le Roux optimized excitation pulse. After the excitation, the Cartesian encoding is performed along the z-axis, simultaneously to the gradient ramp along the x- and y-axes to reach the desired k-space off-center position and velocity. Finally, a sinusoidal gradient wave-form is applied along the x- and y-axis during acquisition to produce the circular trajectory.

Moreover, the implementation of CS acceleration (Candes et al., 2006; Donoho, 2006) relies on two prerequisites. The first is that the signal or image exhibits sparsity in a known transform domain (Knoll, Clason, et al., 2011; Michael et al., 2007). The second is that the data are randomly undersampled, which can be achieved by random undersampling of the k-space in MRI applications. To enable the random sparse undersampling necessary for CS, we utilized a novel approach where successive circular trajectories are randomly positioned in k-space, rather than using regular patterns such as rosette, concentric, or uniformly distributed circles. The acquisition strategy of ECCENTRIC is illustrated in Figure 1. The circle centers’ polar coordinates $\left(r_{c},\phi_{c}\right)$ are chosen randomly with a uniform probability within the ranges $r_{c}\in \left[0,\text{max}\left(k_{x,y}^{max}-R,R\right)\right]$ and $\phi_{c}\in \left[0,2\pi \right)$ (Fig. 1A). Here, R represents the circle radius, $k_{x,y}^{max}$ is the largest in-plane k-space coordinate (assuming the same spatial resolution along all axial plane directions). The majority of circles are placed randomly as shown with two successive circles (c and c+1 ) in the sketch Figure 1A, but with the constraint to avoid significant overlap between circles (Fig. 1B): the distance between the centers of each circle, Δ, must be larger or equal to the Nyquist distance (the inverse of the field-of-view size). When Δ<2R , there is partial overlap between circles; however, this redundant sampling is predominantly concentrated in the central region of k-space, enabling an enhancement in SNR. In addition to the random pattern, a small subset of circles (<5% of the total number) positioned in rosette fashion is acquired in the center of k-space (Fig. 1C). This ensures complete sampling of the center of k-space, which is beneficial for SNR and reconstruction performance (Knoll, Clason, et al., 2011; Michael et al., 2007) with negligible effect on acquisition time. The homogeneous random distribution of circle polar coordinates results intrinsically in a pseudo-random k-space sampling with density following 1/∥k∥ outside of the rosette sampled central region.

The ECCENTRIC design offers the flexibility to choose the radius of circles within the range of 0 to $\frac{k_{x,y}^{max}}{2}$. Therefore, for a given matrix size, field-of-view (FoV), and spectral bandwidth, the radius of ECCENTRIC circles can be chosen to ensure that, even without temporal interleaving, the limits of gradient hardware’s slew rate and amplitude are not exceeded. As detailed below, ECCENTRIC sampling fulfills better the random undersampling required by CS compared to echo-planar (Hu et al., 2008, 2010; Iqbal et al., 2016; Kampf et al., 2010; Otazo et al., 2009; Santos-Díaz & Noseworthy, 2019), spiral (Chatnuntawech et al., 2015), and radial (Saucedo et al., 2021) trajectories.

**Fig. 2.**
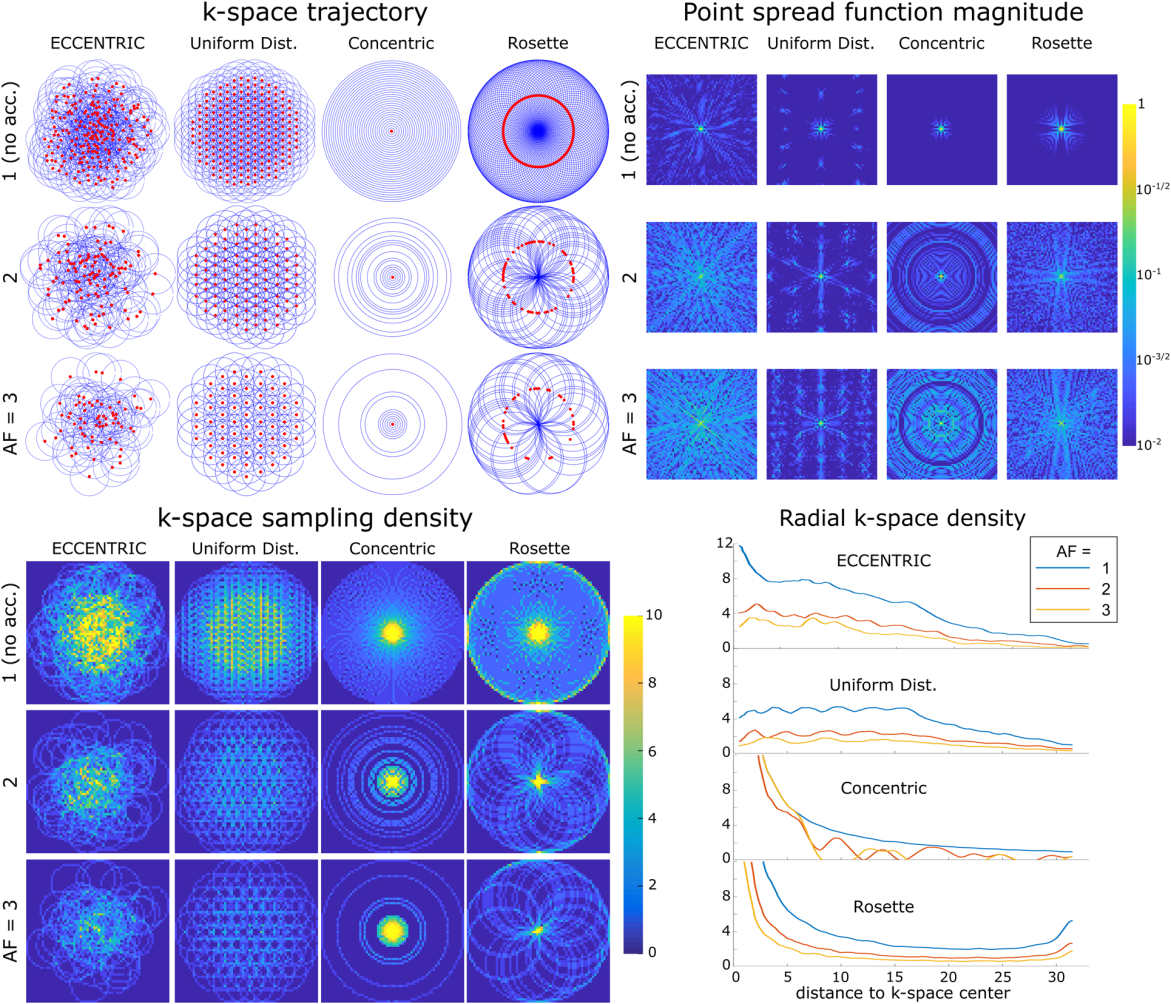
Comparative analysis of circular k-space encodings. Top left, *k*-space trajectories for ECCENTRIC, uniform distributed circles, concentric circles, and rosette trajectories for a 64×64 encoding matrix. Red dots indicate the circle center positions. The acceleration factors AF = 1,2,3 correspond to ECCENTRIC and uniform distributed circles trajectories with 202 , 101 , and 67 circles respectively; 31 , 16 , and 11 concentric circles; 101 , 51 , and 34 Rosette circles. Top right, the point spread function (PSF) calculated for each trajectory and acceleration on a log-scale highlight the presence of incoherent and coherent aliasing patterns. Bottom, the sampling density for the same trajectories and AFs, represented in the 2D k-space (left) and along a radial projection (right).

In Figure 2, a comparison is made between ECCENTRIC, uniform distributed circles trajectory, concentric circles, and rosette sampling. The trajectory and sampling density in the k-space for each pattern and acceleration factor highlight the differences in sampling distribution. While rosette and concentric circle trajectories provide high sampling density at the center of k-space, uniformly distributed circles exhibit a flat and less optimal sampling density in terms of SNR. Accurate reconstruction benefits from sampling the center of k-space, which contains data with the highest SNR. The density distribution of ECCENTRIC lies between that of rosette and concentric circles and uniform distribution. It features a high-density singularity at the k-space center which gradually decreases towards the periphery of k-space.

The point spread function (PSF) simulations in Figure 2 illustrate that ECCENTRIC is inherently suited for the random undersampling necessary to achieve optimal CS performance (Michael et al., 2007). The PSF reflects the interference between voxels in image space resulting from undersampling. Simulations reveal an incoherent pattern for PSF of ECCENTRIC due to the pseudo random k-space sampling, and the PSF pattern spreads with the increasing acceleration factor but conserve the pseudo-random behavior with undersampling (Fig. 2). In comparison, uniform distributed circles, concentric circles, and rosette trajectories have more compact PSFs for fully sampled acquisitions and their PSFs exhibit coherent patterns when undersampling is applied, which is less favorable for CS acceleration. The PSF was computed on a 64×64 matrix and obtained from a single point source (Mareci & Brooker, 1991) that was encoded in k-space and then reconstructed with Non-uniform Fourier transform with Voronoi’s partition density compensation (Rasche et al., 1999).

To extend ECCENTRIC to 3D k-space sampling, a stack of ECCENTRIC is employed with circles randomly placed in the $k_{x}$- $k_{y}$ planes, while $k_{z}$ is encoded using Cartesian phase-encoding (Fig. 1D). The 3D k-space can be covered using spherical or ellipsoid coverage, where the in-plane k-space boundary is defined as $k_{x,y}^{max}=\frac{n}{2FoV}\sqrt{1-{\left(k_{z}/k_{z}^{max}\right)}^{2}}$, with FoV representing the FoV size and n the spatial resolution. The spherical k-space coverage yields an additional acceleration factor of 1.5 compared to cylindrical k-space coverage. To achieve complete sampling in a single $k_{x}$- $k_{y}$ plane, the number of needed ECCENTRIC circles can be derived similar to rosette encoding that requires $\frac{\pi n}{2}$ circles (Schirda et al., 2009). Expanding from the number of points of a fully sampled rosette trajectory to ellipsoidal coverage, the total number of ECCENTRIC circles required for complete sampling of a single partition of the stack is $\frac{\pi nk_{x,y}^{max}}{2R}$ . To achieve circle encoding with off-center position $\left(r_{c},\phi_{c}\right)$, a brief gradient ramp is used to gain an initial momentum $\left(k_{x},k_{y}\right)$ position and the necessary velocity. This process is done at the same time as the slab excitation rewinder overlapped by z-phase encoding and does not increase the echo time, as shown in Figure 1E. In implementing CS acceleration, the total number of ECCENTRIC circles $N_{c}$ is reduced uniformly across the stacks by a factor of AF. Since each circle pattern for every partition of the ECCENTRIC stack is randomly drawn, this results in sparse and random sampling across all three dimensions of the k-space.

Due to the non-uniformity and sparsity of the sampling, a specific model is necessary to reconstruct 4D (k,t ) data of ECCENTRIC into image-frequency space. In previous studies (Klauser et al., 2019, 2021, 2022), we demonstrated the effectiveness of CS-SENSE-LR model that combines partial-separability (or low-rank) with Total-Generalized-Variation (TGV) constraint for reconstructing Cartesian k-space data acquired with random undersampling, leading to improved SNR. Here, we extended the CS-SENSE-LR approach to incorporate non-uniform Fourier sampling necessary for reconstructing ECCENTRIC data. Defining the discrete MRSI data in image space to be ρ as an $N_{\text{r}}$ by T array (with $N_{\text{r}}$ the number of spatial points and T the number of sampling time points), the low-rank hypothesis on the magnetization assumes that the MRSI data can be separated into a small number of spatial and temporal components:

ρ=UV (1)

where U is a $N_{\text{r}}$ by K array and V a K by T array, with K the rank of the low-rank model. These components are retrieved by CS-SENSE-LR reconstruction solving the inverse problem

$$\begin{array}{cccc} \text{arg}\underset{\text{U},\text{V},\text{L}}{\text{min}} & {\left\|\;W\left(\text{s}-ℱCℬ\left(\text{UV}+\text{L}\right)\right)\;\right\|}_{2}^{2} & & \\ & +\lambda \sum_{c=1}^{K}{\text{TGV}}^{2}\left\{U_{c}\right\}. & & \end{array}$$ (2)

where s the measured data, ℱ the non-uniform Fourier transform (NUFT) encoding operator, C the coil sensitivity operator, ℬ the $B_{0}$ frequency shift operator, and L represent the lipid signal ($N_{\text{r}}$ by T array) from skull that is reconstructed simultaneously with brain metabolite U and V but on a separate spatial support. W is a weighting operator of a Hamming window shape, and decreasing with the distance to the center of the k-space (Ban et al., 2019; Klauser et al., 2021). The NUFT encoding operator of ECCENTRIC ℱ is a discrete non-uniform Fourier transform of type 1:

$${\left(ℱ\rho \right)}_{j,t}=\underset{i}{\sum }{\text{e}}^{\text{i}2\pi {\text{k}}_{j}\;\cdot \;{\text{r}}_{i}}\rho_{i,t}$$ (3)

with ${\text{r}}_{i}$ are the uniform image space coordinates and ${\text{k}}_{j}$ k-space sampling-point coordinates located on the 3D ECCENTRIC circles.

${\text{TGV}}^{2}$ is the total generalized variation cost function with λ the regularization parameter (Knoll, Bredies, et al., 2011). The regularization parameter used in the reconstruction was adjusted to $\lambda =3\times 10^{-4}$ by gradually increasing it from a low value until the noise-like artifacts in the metabolite maps disappeared (Klauser et al., 2022; Knoll, Bredies, et al., 2011). The reconstruction rank, K, was determined qualitatively as the minimum number of components that contain some signal distinguishable from noise. For the 3D ECCENTRIC reconstruction, K was specifically set to 40. Additional information regarding the determination of K and an illustration of its impact on the results are available in the Supplementary Material and depicted in Figures S9–S11.

The contamination of U and V by skull-lipid signal is prevented by filtering of the gradient descent during the reconstruction (Eq. 2). Lipid signal is removed from each step of the gradient descent by applying the operator (1−ℙ) with ℙ the lipid subspace projection computed from the estimated lipid signal at the skull L (Klauser et al., 2019). The reconstruction algorithm for U and V, along with the detailed utilization of the lipid suppression operator (1−ℙ), is further described in the Supplementary Material, accompanied by pseudocode for clarity.

## 3 Methods

### 3.1 ECCENTRIC FID-MRSI acquisition parameters

^1^H-FID-MRSI (Bogner et al., 2012; Henning et al., 2009) acquisition was implemented with 3D spherical stack-of-ECCENTRIC sampling as depicted in (Fig. 1E) on a 7T scanner (MAGNETOM Terra, Siemens Healthcare, Erlangen, Germany) running VE12U SP1 software and equipped with NOVA head coils (32Rx/1Tx and 32Rx/8Tx). The echo-time (TE) was set to the minimum possible: 0.9 ms with a 27 degree excitation flip-angle (FA) and 275 ms repetition-time (TR). A slab-selective excitation was performed with a Shinnar-LeRoux optimized pulse (Klauser et al., 2021; Pauly et al., 1991) with 6.5 kHz bandwidth and was preceded by four-pulses WET water suppression scheme (Klauser et al., 2021; Ogg et al., 1994) (Fig. 1). The FoV was 220×220×105 mm^3^ (A-P/R-L/H-F) with 85 mm-thick excited slab. A voxel size of 3.4×3.4×3.4 mm^3^ (40.5 μl ) was realized with a 64×64×31 matrix. The ECCENTRIC circles radius R was set to $1/8\frac{n}{FoV}$ which corresponds to a diameter that encompasses a quarter of the width of the k-space, with n being the square matrix size. The radius R was selected to maximize the number of sampled points per circle, thereby optimizing SSE acceleration, while ensuring not to exceed the gradient system limits for the desired spectral bandwidth. With $R=1/8\frac{n}{FoV}$ , ECCENTRIC enables a spectral bandwidth of 2,280 Hz without the need for temporal interleaving for in-plane FoV = 220 x 220 $mm^{2}$ and 64×64 matrix. The effect of circle radius on the SNR and the quality of metabolic maps was investigated in Supplementary Figures S14–S16. With this radius, ECCENTRIC circles cover 20 points in k-space, inherently providing an SSE acceleration of 20 without undersampling. The FID was sampled with 500 time-points which corresponds to the number of revolutions on each ECCENTRIC circle, and resulted in a total FID duration of 220 ms. To obtain the fully sampled (AF = 1) spherical 3D stack-of-ECCENTRIC with these parameters, a total number of $N_{c}=4,072$ circles is required, which corresponds to 18 min 40 sec acquisition time (TA). For accelerated acquisitions, we decreased the number of circles to $N_{c}/AF$ , with TA being shortened proportionally. For instance, with the same encoding parameters, AF = 2 needs $N_{c}=2,036$ in 9 min 20 sec, AF = 3 needs $N_{c}=1,357$ in 6 min 16 sec, AF = 4 needs $N_{c}=1,018$ in 4 min 40 sec, and so on.

A rapid calibration scan of water reference data was performed by turning off water suppression and using the same FoV, FA slab excitation, TR, FID duration, and spectral bandwidth, but with rosette trajectory sampling at lower resolution (23×23×19 ) in 1 min 16 sec.

The $B_{0}$ shimming of the 85 mm thick whole-brain slab was performed using the manufacturer methods that adjusted the shim currents over 12 spherical harmonics coils: three 1st order, five 2nd order, and four 3rd order. The global linewidth of the water over the entire 85 mm slab was between 25–42 Hz across all subjects. In the majority of the subjects the global water linewidth was between 30–35 Hz. Adjustment of the $B_{1}+$ transmit and water suppression was subsequently performed with manufacturer methods. The entire adjustment procedure took between 1–2 min for every subject.

### 3.2 Reconstruction of ECCENTRIC FID-MRSI metabolic images

The rapid water reference data were used to compute the coil sensitivity maps using ESPIRiT (Uecker et al., 2014) (C operator in Eq. 2) and to estimate a $\Delta {\text{B}}_{0}$ field map with *multiple signal classification algorithm* (MUSIC) (Gruber & Hayes, 1997). The field correction operator in Equation 2 was then determined by $ℬ={\text{e}}^{\text{i}2\pi t\gamma \Delta {\text{B}}_{0}}$, where $\gamma \Delta {\text{B}}_{0}$ is the spatial frequency shift caused by the field inhomogeneity map (in Hz). To reconstruct 3D ECCENTRIC FID-MRSI data and obtain the metabolic images, we employed a comprehensive pipeline that included: 1) water removal using the HSVD method (Barkhuijsen et al., 1987) for each coil channel (Klauser et al., 2019), 2) determination of the $\Delta {\text{B}}_{0}$ field map and the coil sensitivity maps from the rapid water reference data, 3) CS-SENSE-LR reconstruction model from Equation 2, which includes simultaneous suppression of scalp lipid signals, and 4) spectral fitting by LCModel software (Provencher, 1993) with the reconstructed rapid water reference data serving as the normative signal. Because FID gradient-echo excitation does not refocus chemical shift evolution during echo-time the spectra need first order phase correction, which was performed by backward linear prediction of the evolution (Nassirpour, Chang, & Henning, 2018a). A metabolite basis obtained by quantum mechanics simulations in GAMMA (Smith et al., 1994) was utilized to fit and quantify 21 metabolites: phosphocholine (PCh), glycerophosphocholine (GPC), creatine (Cr), phosphocreatine (PCr), gamma-aminobutyric acid (GABA), glutamate (Glu), glutamine (Gln), glycine (Gly), glutathione (GSH), myo-inositol (Ins), N-acetylaspartate (NAA), N-acetyl aspartylglutamate (NAAG), scyllo-inositol (Sci), lactate (Lac), threonine (Thr), beta-glucose (bGlu), alanine (Ala), aspartate (Asp), ascorbate (Asc), serine (Ser), and taurine (Tau). Phosphorylcholine and glycerophosphorylcholine were combined into total choline-containing compounds (Cho), while creatine and phosphocreatine were combined into total creatine (tCre). Concentration maps were then generated for the metabolites included in the simulated basis.

The water reference signal was used as quantification reference by LCModel, and the resulting concentration estimates were expressed in institutional units (I.U.). This allowed for comparisons of metabolite levels across both subjects and different metabolites. The ultra-short TE used in the ECCENTRIC MRSI data acquisition meant that $T_{2}$ relaxation correction was unnecessary for both metabolite and water signals. As a result of employing a short TR with Ernst flip angle, metabolite maps may contain $T_{1}$-weighted contrast. Correcting for this would necessitate measuring $B_{1}+$ field and incorporating prior knowledge of metabolite $T_{1}$. The results of the LCModel fitting for each voxel were further used to generate spatial maps of the concentration of each metabolite. To assess the quality of the MRSI data and the goodness of fit, quality control maps of Cramer-Rao lower bounds (CRLB), line-width (FWHM), and SNR were generated from the LCModel fitting.

### 3.3 ECCENTRIC FID-MRSI in high-resolution phantom

**Fig. 3.**
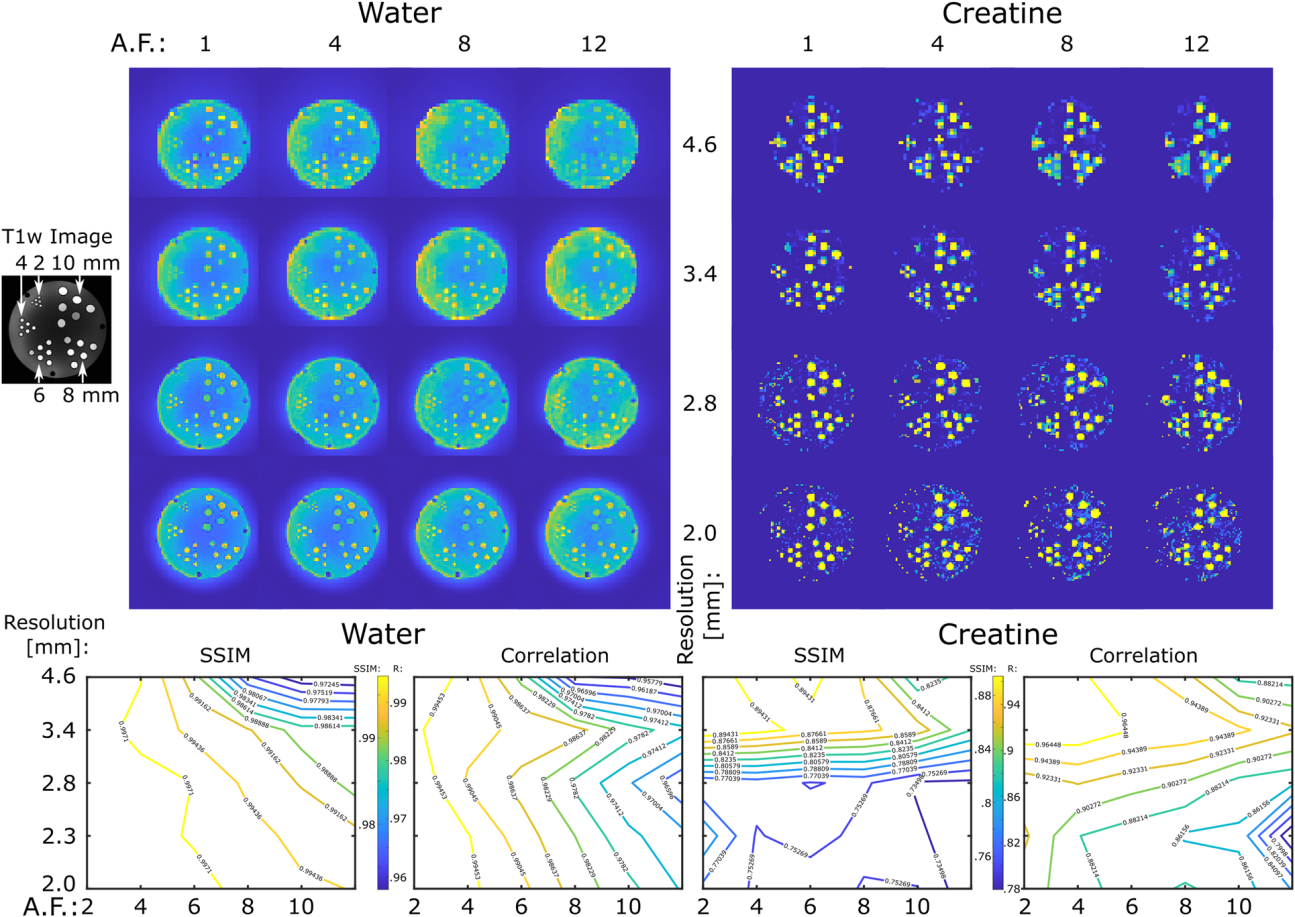
ECCENTRIC imaging of water and metabolites in the high-resolution structural-metabolic phantom. ECCENTRIC performance was tested for spatial resolutions of 4.6, 3.4, 2.8, 2.0 mm and acceleration factors between 1–12. Top, examples of water and creatine images are shown for all 4 resolutions and 4 acceleration (AF: 1,4,8,12). Bottom, SSIM and correlation factors for each resolution and acceleration are calculated considering as ground truth the fully sampled image (AF = 1).

Experimental performance of ECCENTRIC sampling was first tested on a high-resolution structural-metabolic phantom. We used a custom-made phantom with geometry similar to Derenzo molecular imaging phantom (Derenzo et al., 1977) containing 5 sets of tubes with diameters of 2, 4, 6, 8 and 10 mm as shown in Figure 3. Each set contained 6 tubes of identical diameter separated by a distance equal to twice the inner diameter positioned in a triangular configuration. In every set, the six tubes were filled with metabolite solutions containing 10 mM of creatine. Magnevist (Gd-DTPA) was added (1 mL/L) in each tube to shorten $T_{1}$ and create $T_{1}$-weighted contrast for structural MRI. The whole tube structure was inserted in a large cylindrical container (13.33 cm inner diameter) which was filled with 10 mM NaCl solution. Further details of phantom manufacturing and chemical composition are mentioned in Klauser et al. (2021).

Due to the phantom’s geometric structure being present only in the axial section, we opted for a 2D ECCENTRIC acquisition. The sampling scheme for 2D ECCENTRIC is identical to the central k-space partition used in 3D ECCENTRIC. The 2D ECCENTRIC acquisition maintained the same RF-pulse and FA but required slightly longer TE to 1.15 ms, and the TR was set to 450 ms to accommodate the longer $T_{1}$ relaxation times in the phantom. The FID was measured with a spectral bandwidth of 2,000 Hz over 350 ms, and successive acquisitions were performed with increased in-plane resolution. The circle radius (R) was set to $\frac{n}{8FoV}$ , $\frac{n}{8FoV}$ , $\frac{n}{9FoV}$ , and $\frac{n}{10FoV}$ for 4.6 , 3.4 , 2.8 , and 2.0 mm in-plane resolutions, respectively, to avoid temporal interleaving for any spatial resolution.

In addition to metabolite imaging, we conducted water imaging of the structural phantom at identical spatial resolutions to more comprehensively evaluate the spatial encoding performance of ECCENTRIC. To accomplish this, we employed the 2D ECCENTRIC sequence without water suppression, utilizing a short TR of 100 ms and an FA of 40 degrees. This choice was made to maximize the $T_{1}$-weighted ($T_{1}$w) contrast of the tubes within the cylindrical phantom. Subsequently, the first point of the acquired timeseries was reconstructed to generate the $T_{1}$-weighted water image. Both metabolite and water data were acquired with fully sampled ECCENTRIC. To investigate accelerations, the fully sampled ECCENTRIC data were retrospectively undersampled for acceleration factors (AF) between 2−12 .

The effect of the acceleration on water and metabolite imaging was evaluated by analyzing the structural similarity index (SSIM) and correlation coefficient for all voxels inside the phantom with respect to the fully sampled data (Fig. 3).

### 3.4 ECCENTRIC FID-MRSI in healthy volunteers

Five healthy volunteers were scanned at Athinoula A. Martinos Center For Biomedical Imaging for this study. The protocol was approved by the institutional ethics committee, and written informed consent was given by all subjects before participation. The 3D ECCENTRIC FID-MRSI sequence described above was acquired with voxel size of 3.4 mm isotropic at AF = 1,2,3 and 4 successively. In two volunteers, the performance of 3D ECCENTRIC FID-MRSI was also tested at ultra-high resolution with voxel size of 2.5 mm isotropic (matrix 88×88×43 , AF = 4, TA = 10 min 26 sec) and compared to 3.4 mm isotropic (matrix 64×64×31 , AF = 2, TA = 9 min 20 sec). Our objective was to determine the feasibility of achieving a metabolic imaging protocol that delivers close to 3 mm isotropic whole-brain coverage in under 10 min using ECCENTRIC.

All volunteers were scanned with a $T_{1}$-weighted anatomical MP2RAGE sequence (Marques et al., 2010) (1 mm isotropic, 4,300 ms TR, 840 ms and 2,370 ms TI) for positioning of the MRSI FoV and for the generation of skull-masks that are needed for the lipid removal during the reconstruction and to exclude voxels located outside the head volume.

### 3.5 Quantitative analysis

For quantitative analysis of 3D ECCENTRIC FID-MRSI, the metabolite concentrations (I.U.) were analyzed in each brain lobe and tissue type. This involved segmenting MP2RAGE images into gray matter, white matter, and cerebrospinal fluid using Freesurfer version 7.1.1 software (Fischl et al., 2002). Cerebral lobes were then identified utilizing a standard atlas, and a general linear model was employed to estimate metabolite concentrations within each atlas-defined structure (Klauser et al., 2022).

### 3.6 Reproducibility analysis

The reproducibility of metabolite maps measured by 3D ECCENTRIC FID-MRSI were assessed by repeated imaging in four healthy volunteers. All scans were conducted sequentially without repositioning the volunteer. For the reproducibility analysis, three data sets with AF=3 in each volunteer were compared. One data set was acquired with AF=3 , and the other two datasets were obtained by retrospective undersampling to AF=3 the data acquired with AF=1 and AF=2 . Coefficients-of-variation (COV) were computed for individual anatomical regions from the three data sets. Both inter-measurement and inter-subject COVs were calculated and then averaged across subjects.

## 4 Results

### 4.1 ECCENTRIC imaging in high-resolution phantom

In the series of water imaging experiments, ECCENTRIC can resolve the structural details of the phantom up to the resolution targeted by the imaging protocol as can be seen in Figure 3.

For water images, no visible difference in image quality can be seen for retrospective accelerations factors up to AF = 4, minor changes can be detected for AF between 4–8, and moderate loss of details for AF between 8–12 when compared to the fully sampled acquisition (AF = 1). Considering AF = 1 as ground truth, quantitative analysis reveals that SSIM ≥ 0.99 across all resolutions for accelerations up to AF = 4, and SSIM decreases to 0.97 for the highest acceleration and resolution tested. Similarly, correlation factors larger than 0.99 are observed up AF = 4, which decrease to 0.95 for the lowest resolution and largest acceleration factor. Comparing the different spatial resolutions, the higher CS accelerations show better performance for higher resolution images.

In the series of metabolite imaging experiments, ECCENTRIC was used to image the creatine metabolite present in the tubes with the same resolutions and acceleration factors as in the water imaging (Fig. 3). These results of these experiments show that: 1) metabolite maps exhibit comparable quality for AF between 1–4, 2) for AF>4 there is reduction in image details and an elevation of the noise level. Considering AF = 1 as ground truth, across the entire series of measurements SSIM range between 0.75–0.89, and correlation factors between 0.79–0.96. Unlike the water imaging results, the increasing acceleration does not yield higher SSIM and correlation factors as the spatial resolution increases. This discrepancy is likely attributed to the much lower ($10^{3}-10^{4}$ less) SNR of metabolites in comparison to water, which becomes critical for the smallest voxel size. In particular, we note that for isotropic voxel size of 3.4 mm and for acceleration factors up to 4 we obtained the highest SSIM and correlation factors for metabolic imaging.

### 4.2 ECCENTRIC FID-MRSI in healthy volunteers

**Fig. 4.**
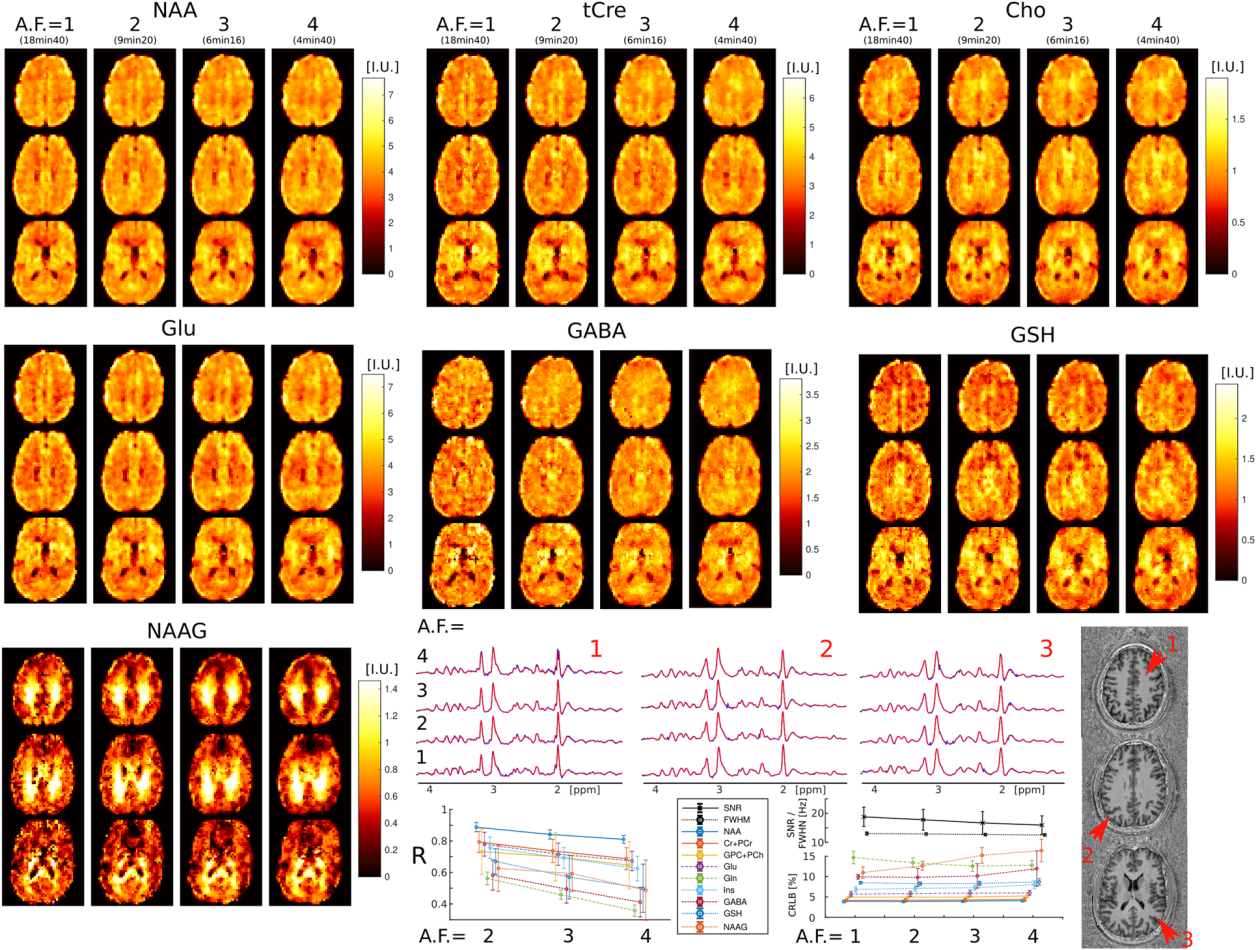
3D ECCENTRIC FID-MRSI metabolic images of human brain acquired in a healthy volunteer with 3.4 mm isotropic voxel size and CS acceleration factors AF = 1–4. Top, metabolite maps of seven relevant brain metabolites (NAA, tCre, Cho, Glu, GABA, GSH, and NAAG) are shown for all acceleration factors (AF). Spectra from three brain locations indicated by red arrows on the anatomical image. At the bottom, the left plot displays the correlation coefficients between accelerated images (AF=2,3,4 ) and fully sampled images (AF=1 ), while the right plot shows the LCModel quantification error (CRLB), linewidth (FWHM), and SNR.

Examples of metabolic images for seven metabolites obtained with retrospective AF=1−4 are shown in Figure 4. Very similar structural details and tissue contrast of metabolic images are obtained for all accelerations compared to the fully sampled data. This is visible also by inspecting spectra that show the same metabolic profile across acceleration factors (AF=1−4 ). The CS accelerations (AF=2,3,4 ) were achieved by retrospectively undersampling the fully acquired data (AF=1 ). The purpose of this was to focus the analysis on the effects of CS acceleration, while avoiding any image differences that could be caused by head motion during different acquisitions. To illustrate the effect of each component of the CS-SENSE-LR reconstruction, we modified the model to isolate and evaluate each feature independently. The results are presented in Supplementary Material Section II.B and Figure S8, and are consistent with previously published data obtained using similar reconstruction models for Cartesian CS FID-MRSI (see supplementary material in Klauser et al. (2021)). To validate the low-resolution rapid water reference measurement described in Section 3.1 and used for the reconstruction and quantification, we compared it in Supplementary Figure S12 with a water 3D ECCENTRIC FID-MRSI reference acquisition, which matched the spatial resolution of the metabolite measurement.

Visual inspection of metabolic images reveal that: 1) tCre, Glu, and GABA have larger signal in gray matter than white matter, 2) NAA has more signal in gray than white matter, but with lower gray-white matter contrast compared to tCre, Glu, and GABA, 3) Cho has higher signal in frontal white matter than gray matter, and 4) NAAG has the largest contrast from all metabolites, with much larger signal in white matter compared to gray matter. Metabolic images obtained with AF=1 and AF=2 are largely identical. Minor blurring of fine structural details starts to become noticeable for AF≥3 , however adequate delineation of gray-white matter folding is maintained up to AF=4 . Additionally, the performance of ECCENTRIC to reveal brain structural details was probed also by water imaging in four healthy volunteers. Brain water imaging by ECCENTRIC shows comparable performance for AF between 1–10 (Supplementary Fig. S13), similar to the phantom results.

Quantitative image analysis shows that the correlation between the accelerated and fully sampled metabolic images is high (R > 0.7) for metabolites that have a high SNR (>10 ) such as NAA, Cho, tCre, Glu, and Ins, while metabolites of lower SNR such as Gln, GABA, GSH, and NAAG exhibit lower correlations (R = 0.4−0.7 ). The error (CRLB) of spectral fitting is below 20%, which indicates very high goodness-of-fit by LCModel software. The CRLB does not degrade with the acceleration factor, except for NAAG and GABA, although even in this case it does not exceed the 20% limit. The SNR shows only a minor decrease between AF=1 (SNR = 17) and AF=4 (SNR = 15), while the linewidth does not degrade with acceleration.

### 4.3 Quantitative analysis of ECCENTRIC metabolic imaging

Table 1 presents brain regional concentrations of nine metabolites quantified using water signal as reference and expressed in institutional units (I.U.). The concentrations were calculated in the gray and white matter of the five major brain lobes across the five healthy volunteers. Our results indicate that: 1) six metabolites have higher concentrations in gray-matter compared to white matter (GM/WM = 1.19 tCre, 1.12 NAA, 1.2 Glu, 1.34 Gln, 1.15 GABA, 1.11 GSH); 2) two metabolites have higher concentrations in white-matter compared to gray-matter (GM/WM = 0.96 Cho, 0.47 NAAG); and 3) one metabolite has region-dependent gray/white-matter ratio (GM/WM = 1.17–0.87 Ins). The largest gray-white matter contrast is exhibited by NAAG due to its specific compartmentalization in white matter.

**Table 1.** Metabolite concentrations (I.U.) and quantification error (Cramér-Rao lower bound, CRLB %) in each brain lobe and tissue type.

Mean across volunteers	Frontal		Limbic		Parietal		Occipital		Temporal		Usable voxels
(Standard deviation)	WM	GM	WM	GM	WM	GM	WM	GM	WM	GM	Mean % (std)
tCre [I.U.]	3.34 (0.34)	3.84 (0.04)	3.22 (0.35)	4.46 (0.28)	3.30 (0.28)	3.95 (0.28)	3.31 (0.30)	3.39 (0.19)	3.09 (0.25)	3.81 (0.19)	
tCre CRLB [%]	3.25 (0.24)	3.54 (0.41)	3.38 (0.31)	3.18 (0.30)	3.21 (0.33)	3.26 (0.26)	3.45 (0.62)	3.64 (0.62)	3.42 (0.37)	3.33 (0.26)	73.3 (3.3)
NAA [I.U.]	4.16 (0.98)	4.51 (0.66)	4.07 (1.01)	5.38 (0.90)	4.31 (0.96)	4.84 (0.88)	4.33 (0.98)	4.18 (0.85)	3.91 (0.84)	4.35 (0.73)	
NAA CRLB [%]	3.13 (0.34)	3.55 (0.64)	3.26 (0.33)	3.07 (0.36)	2.86 (0.26)	3.05 (0.37)	3.30 (0.54)	3.63 (0.64)	3.32 (0.39)	3.32 (0.34)	73.0 (3.5)
Ins [I.U.]	3.49 (0.52)	3.51 (0.35)	3.65 (0.60)	4.28 (0.48)	3.73 (0.66)	3.58 (0.29)	3.74 (0.65)	3.27 (0.57)	3.56 (0.65)	3.39 (0.51)	
Ins CRLB [%]	5.33 (0.64)	6.04 (0.94)	5.29 (0.52)	5.36 (0.42)	5.16 (0.42)	5.63 (0.52)	5.38 (0.48)	5.84 (0.50)	5.34 (0.33)	5.68 (0.40)	73.0 (3.5)
GPC+PCh [I.U.]	1.03 (0.10)	0.93 (0.05)	1.10 (0.10)	1.24 (0.08)	1.06 (0.11)	0.95 (0.09)	0.94 (0.12)	0.79 (0.06)	0.99 (0.07)	0.98 (0.12)	
GPC+PCh CRLB [%]	3.63 (0.34)	4.24 (0.59)	3.46 (0.31)	3.56 (0.27)	3.56 (0.29)	3.97 (0.33)	4.13 (0.57)	4.59 (0.64)	3.68 (0.29)	3.94 (0.25)	73.5 (3.3)
Glu [I.U.]	3.86 (0.53)	4.50 (0.29)	3.72 (0.49)	5.24 (0.46)	3.86 (0.39)	4.61 (0.39)	3.80 (0.36)	3.82 (0.38)	3.50 (0.35)	4.29 (0.44)	
Glu CRLB [%]	4.51 (0.51)	5.01 (0.85)	4.72 (0.59)	4.37 (0.47)	4.31 (0.53)	4.48 (0.55)	4.82 (1.14)	5.22 (1.32)	4.80 (0.74)	4.71 (0.64)	72.8 (3.8)
Gln [I.U.]	0.89 (0.06)	1.24 (0.25)	0.83 (0.06)	1.33 (0.16)	0.80 (0.16)	1.08 (0.26)	0.87 (0.22)	0.91 (0.21)	0.80 (0.09)	1.07 (0.22)	
Gln CRLB [%]	15.93 (2.45)	14.81 (2.22)	17.72 (2.68)	14.38 (2.48)	17.92 (4.78)	16.22 (3.24)	18.99 (5.14)	18.77 (4.11)	18.30 (2.95)	16.45 (2.60)	56.3 (4.6)
GABA [I.U.]	1.34 (0.09)	1.48 (0.29)	1.34 (0.10)	1.79 (0.37)	1.37 (0.14)	1.58 (0.30)	1.33 (0.18)	1.37 (0.18)	1.21 (0.16)	1.39 (0.32)	
GABA CRLB [%]	8.68 (1.10)	9.61 (1.51)	8.86 (1.50)	8.52 (1.74)	8.54 (2.08)	8.78 (1.76)	9.48 (2.34)	9.90 (2.22)	9.60 (2.16)	9.52 (2.09)	68.3 (1.5)
GSH [I.U.]	1.07 (0.24)	1.22 (0.05)	1.11 (0.26)	1.45 (0.20)	1.10 (0.20)	1.17 (0.07)	1.05 (0.18)	1.00 (0.09)	1.02 (0.17)	1.14 (0.12)	
GSH CRLB [%]	8.20 (0.88)	8.77 (1.15)	8.29 (0.93)	7.96 (0.79)	8.04 (1.00)	8.43 (0.87)	9.12 (2.01)	9.80 (1.90)	8.65 (1.23)	8.81 (1.17)	71.5 (3.1)
NAAG [I.U.]	0.82 (0.14)	0.39 (0.12)	0.90 (0.13)	0.49 (0.17)	0.96 (0.17)	0.34 (0.13)	0.78 (0.16)	0.41 (0.12)	0.76 (0.11)	0.35 (0.10)	
NAAG CRLB [%]	14.11 (4.10)	19.02 (5.07)	12.43 (3.87)	17.35 (6.07)	12.01 (3.14)	18.03 (5.15)	15.51 (5.08)	19.40 (6.62)	15.36 (5.01)	20.13 (6.40)	45.3 (11.6)
SNR	24.64 (4.41)	23.67 (3.84)	22.92 (4.50)	24.53 (4.02)	25.64 (4.82)	24.73 (4.17)	22.48 (6.13)	21.32 (5.56)	22.07 (4.23)	22.40 (3.37)	∅
FWHM [Hz]	11.43 (0.91)	12.09 (0.77)	11.28 (1.15)	11.13 (1.13)	10.21 (0.79)	11.02 (0.84)	11.64 (0.91)	12.33 (1.04)	12.26 (1.23)	12.38 (1.22)	∅

The two bottom rows present the SNR and FWHM values. The last column shows the percentage of voxels inside the brain and FoV that meet the criteria of good quality: CRLB <20%, FWHM <0.07 ppm, SNR >5. The values are calculated as the average (standard deviation) across the healthy volunteers imaged by 3D ECCENTRCIC at 3.4 mm isotropic with AF=2 (9 min:20 sec acquisition time).

Quantitative parameters for the quality of MRSI data are also listed in Table 1, including the precision of metabolite quantification by the Cramer-Rao lower bounds (CRLB), the SNR, and spectral linewidth (FWHM). It is worth noting that the values of spectral SNR and fitting CRLB are influenced by our reconstruction method. The CS-SENSE-LR reconstruction incorporates a low-rank model, effectively reducing noise, thereby enhancing SNR and lowering CRLB values. Consequently, the SNR and CRLB metrics not only reflect the acquisition quality but also the effectiveness of the reconstruction process. Therefore, these values may vary from studies that do not employ similar reconstruction techniques. It can be seen that mean CRLB is below <20% for all the metabolites across the imaged whole-brain volume. In particular, mean CRLB is below <6% for the five metabolites with highest SNR (NAA, tCre, Cho, Glu, and Ins), between 8% –10% for two metabolites (GABA and GSH) and between 14% –20% for other two metabolites (Gln and NAAG). Across the brain, the mean SNR after the denoising reconstruction is larger than 20 and the mean linewidth is less than 12 Hz (0.04 ppm).

### 4.4 Reproducibility of ECCENTRIC metabolic imaging

**Fig. 5.**
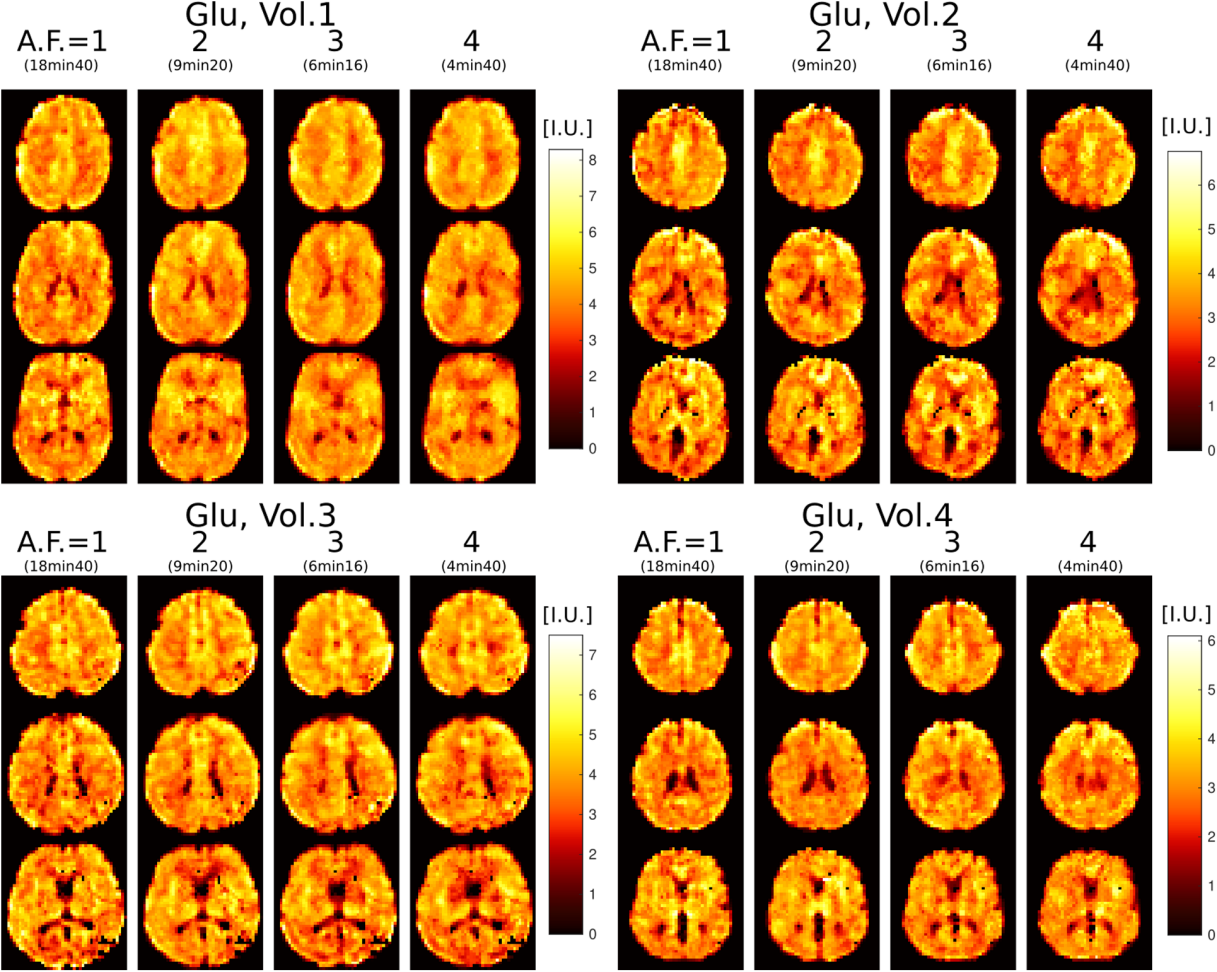
Glutamate imaging at 3.4 mm isotropic voxel size in four healthy volunteers scanned with 3D ECCENTRIC FID-MRSI in four successive acquisitions with increasing accelerations AF = 1, 2, 3, and 4. Three slices are shown for each volunteer at each acceleration.

Results from repeated measurements are shown in Figure 5 for Glu imaging. Due to high concentration of Glu in gray mater, Glu images have high gray-white matter contrast and show fine structural details of brain that can be used to visually assess the stability of the imaging with increasing AF. It can be seen that across all four scans in all four subjects the metabolite images appear visually similar. We note that with repeated measurements some anatomical differences may also be attributed to slight head motion. All metabolite maps, along with their respective CRLB, SNR, and FWHM maps for all four volunteers and AF, are presented in Supplementary Figures S1–S7.

The inter-measurement COV for mapping metabolite concentrations across brain regions are presented in Table 2 and the inter-subject COV in Table 3. Inter-measurement COV smaller than 7% are observed for five metabolites (NAA, tCre, Ins, Cho, Glu) that are the most abundant in the brain. COV between 8%−14% are obtained for Gln, GSH, and GABA. NAAG has higher COV in brain regions (gray matter) where its concentration is low.

**Table 2.** The inter-measurement coefficient of variation (COV) for each metabolite determined in every lobe and tissue type for the 3D ECCENTRIC FID-MRSI acquired at 3.4 mm isotropic resolution in 6 min:16 sec (AF = 3).

	Inter-measurement COV									
	Frontal		Limbic		Parietal		Occipital		Temporal	
	WM	GM	WM	GM	WM	GM	WM	GM	WM	GM
NAA	0.06	0.03	0.06	0.05	0.06	0.05	0.05	0.07	0.06	0.06
tCre	0.05	0.05	0.05	0.03	0.03	0.04	0.04	0.03	0.06	0.05
Ins	0.05	0.04	0.07	0.06	0.05	0.05	0.04	0.06	0.06	0.07
GPC+PCh	0.04	0.03	0.03	0.04	0.04	0.06	0.03	0.04	0.03	0.05
Glu	0.05	0.03	0.05	0.05	0.07	0.07	0.07	0.07	0.06	0.07
Gln	0.14	0.10	0.15	0.11	0.12	0.12	0.12	0.07	0.14	0.08
GABA	0.07	0.11	0.05	0.07	0.09	0.12	0.05	0.11	0.06	0.11
GSH	0.12	0.10	0.14	0.13	0.13	0.13	0.13	0.10	0.14	0.11
NAAG	0.23	0.41	0.28	0.33	0.24	0.55	0.26	0.40	0.28	0.54

**Table 3.** The inter-subject coefficient of variation (COV) for each metabolite determined in every lobe and tissue type for the 3D ECCENTRIC FID-MRSI acquired at 3.4 mm isotropic resolution in 6 min:16 sec (AF = 3).

	Inter-subject COV									
	Frontal		Limbic		Parietal		Occipital		Temporal	
	WM	GM	WM	GM	WM	GM	WM	GM	WM	GM
NAA	0.21	0.15	0.23	0.17	0.23	0.17	0.24	0.23	0.23	0.22
tCre	0.10	0.05	0.12	0.07	0.11	0.05	0.09	0.09	0.11	0.09
Ins	0.18	0.15	0.22	0.17	0.22	0.14	0.21	0.26	0.24	0.19
GPC+PCh	0.06	0.07	0.07	0.06	0.10	0.10	0.12	0.14	0.11	0.15
Glu	0.19	0.13	0.20	0.16	0.20	0.15	0.18	0.18	0.18	0.20
Gln	0.16	0.25	0.12	0.16	0.18	0.21	0.21	0.23	0.17	0.18
GABA	0.37	0.33	0.39	0.38	0.36	0.36	0.38	0.39	0.37	0.40
GSH	0.13	0.09	0.15	0.11	0.13	0.07	0.11	0.13	0.11	0.12
NAAG	0.32	0.37	0.35	0.29	0.45	0.47	0.50	0.48	0.38	0.29

The results from Tables 2 and 3 show that 3D ECCENTRIC FID-MRSI had reproducible and stable performance in three repeat measurements. The inter-measurement COV exhibits a markedly lower value (2–4 times smaller) compared to the inter-subject COV. The former is primarily influenced by technical variability, whereas the latter reflects a combination of technical and biological variability. The quantification of the five main metabolites that have the highest SNR in brain MRSI (NAA, tCre, Cho, Ins, Glu) shows the lowest variability, with a slight increase in the case of less abundant metabolites (Gln, GABA, and GSH). The highest variability is noticed for NAAG outside of the fronto-parietal white matter due to its specific localization in this brain area. We note that in-vivo variability of metabolite quantification in repeat measurements is also influenced by patient motion and scanner stability in addition to 3D ECCENTRIC FID-MRSI, hence methods that reduce the effects of motion and field drift (Andronesi et al., 2021; Bogner et al., 2014) are likely to reduce variability.

### 4.5 Ultra-high resolution metabolic imaging of human brain using 3D ECCENTRIC FID-MRSI

**Fig. 6.**
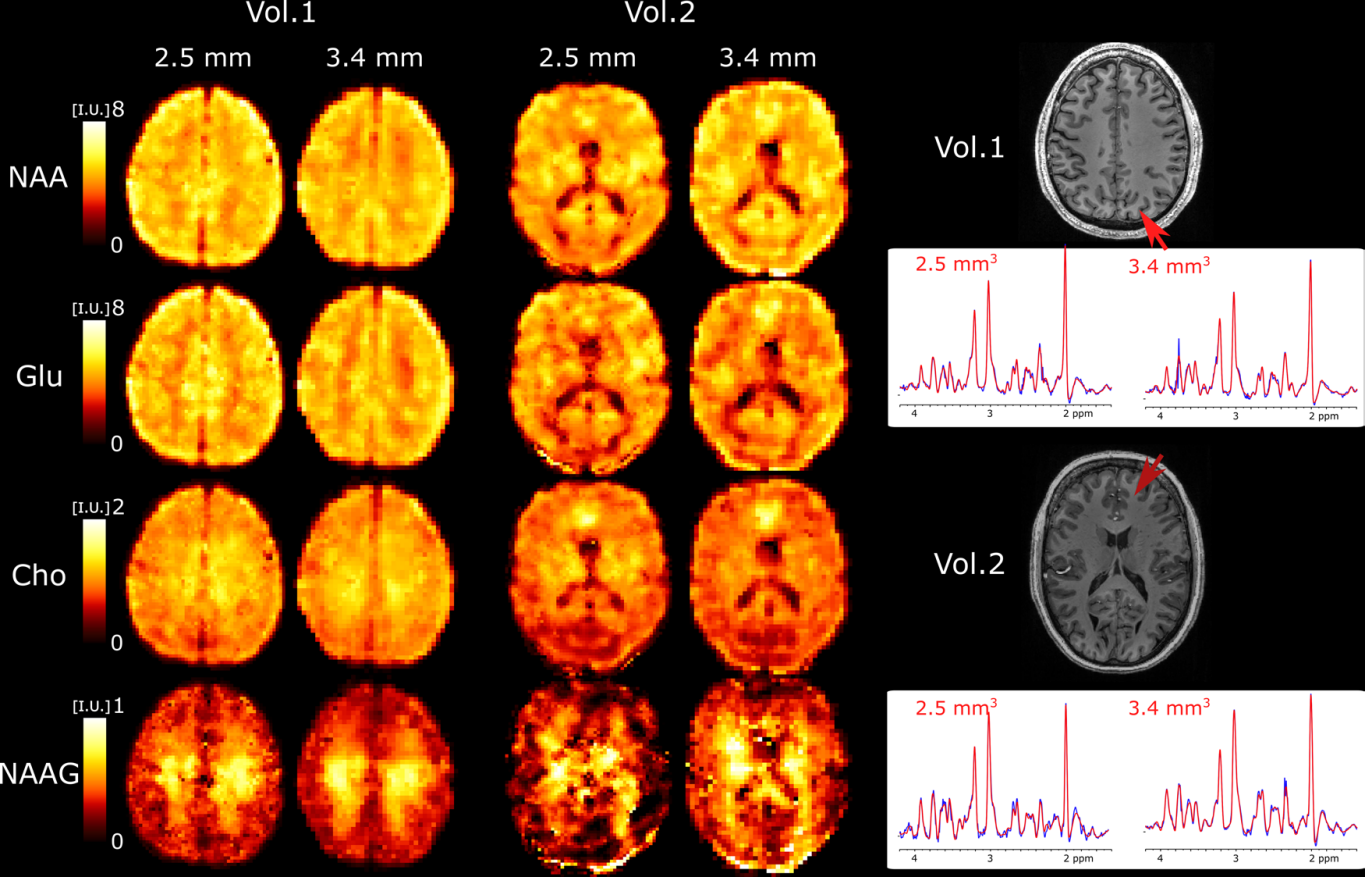
Ultra-high resolution metabolic imaging acquired with 3D ECCENTRIC FID-MRSI at 2.5 mm isotropic voxel size (AF=4 , TA = 10min:26s ) in two healthy volunteers. The ultra-high resolution metabolic imaging is compared to 3D ECCENTRIC FID-MRSI at the typical voxel size of 3.4 mm isotropic (AF=2 , TA = 9min:20s ). Right, two spectra from both spatial resolution and corresponding to the red arrow location are shown. The blue line represents the MRSI data, and the red line is the fit performed by LCModel.

We further explored the performance of 3D ECCENTRIC FID-MRSI for ultra-high resolution metabolic imaging in several healthy volunteers. Based on the high SNR of the 3.4 mm data after the denoising reconstruction, we expected that smaller voxels at higher resolution will still provide sufficient SNR for metabolite imaging. Figure 6 shows metabolic images obtained using 3D ECCENTRIC FID-MRSI with isotropic voxel size of 2.5 mm in two healthy volunteers.

To achieve a feasible scan time, we used CS with AF=4 . We demonstrated at the beginning of our work that AF=4 provides metabolic maps that are similar to those obtained through fully sampled 3D ECCENTRIC FID-MRSI. The AF=4 acceleration enabled the acquisition of 3D ECCENTRIC FID-MRSI at 2.5 mm isotropic resolution in 10 min:26 sec. For comparison, we also acquired the typical 3D ECCENTRIC FID-MRSI at 3.4 mm isotropic with AF=2 acceleration in 9 min:20 sec. As readily apparent by visual inspection, the metabolic maps at higher spatial resolution provide sharper delineation of the brain structure. No compromise is visible for signal-to-noise, contrast-to noise or other data quality metric at ultra-high resolution compared to typical resolution. We note that the acquisition time of 3D ECCENTRIC FID-MRSI at 2.5 mm with AF=4 is only slightly longer (1 min) than at 3.4 mm with AF=2 . However, for the same acceleration factor the acquisition time of 3D ECCENTRIC FID-MRSI at 3.4 mm is 2.2 times faster than at 2.5 mm.

## 5 Discussion

Our results here demonstrate that 3D ECCENTRIC FID-MRSI at 7T can simultaneously image an extended neurochemical panel of 10–14 metabolites with high SNR at high spatial resolution across whole-brain and with acquisition times that are feasible for human imaging. Particularly, we showed that the acquisition of fast non-Cartesian MRSI can be further accelerated up to 4-fold by CS, allowing metabolic imaging at 3.4 mm isotropic resolution in 4 min:40 sec and at 2.5 mm isotropic resolution in 10 min:26 sec, respectively. The CS-SENSE-LR reconstruction produces metabolic images with an effective voxel size identical to the nominal size (Klauser et al., 2021). This provides an advantage compared to other filtered reconstructions (Hangel et al., 2020; Hingerl et al., 2020) which increase the effective voxel volume. ECCENTRIC preserves the features of metabolic images across accelerations. When accelerated up to 4-fold by CS the loss of image quality is minor and the metabolic images effectively visualize the laminar structure of the brain similar to the unaccelerated (AF = 1) ground truth. Through the design of ECCENTRIC acquisition and denoising reconstruction, SNR is enhanced even at high accelerations. This enhancement is achieved by fully sampling the center of k-space and employing a low-rank reconstruction technique.

Here, we investigated the performance of 3D ECCENTRIC FID-MRSI for two applications scenarios: 1) high resolution metabolic imaging (3.4 mm in 4 min:40 sec) for studies that need to minimize imaging time, and 2) ultra-high resolution metabolic imaging (2.5 mm in 10 min:26 sec) for applications that need to probe brain neurochemistry with highest structural detail. Both of these protocols represent a significant advancement for non-invasive imaging of human brain metabolism by *in vivo* MRSI. Their performance level is comparable to other advanced MR imaging methods, such as CEST and perfusion imaging.

Results obtained with the 3.4 mm imaging protocol show good delineation of brain structures. At 2.5 mm ultra-high resolution there is increased gray-white matter contrast of metabolites due to less partial volume effect which reveals the brain folding more clearly than at 3.4 mm. Several metabolites show particularly high contrast between gray and white matter in healthy brain, such as the energy buffer tCre, the neurotransmitter Glu, and the dipeptide NAAG. In particular, NAAG is the most abundant dipeptide in the brain, which is selectively localized in several regions (Pouwels & Frahm, 1998) where it neuromodulates the glutamatergic synapses required for normal brain activity. Importantly, NAAG is also implicated in neurodegenerative diseases, schizophrenia, stroke, epilepsy, traumatic brain injury and pain (Morland & Nordengen, 2022). Our data show the highest resolution of 3D imaging for NAAG to date. 3D ECCENTRIC FID-MRSI provides images of NAAG brain distribution, which could offer valuable insights into both basic and clinical neuroscience questions. Good-quality metabolic images are obtained also for some of the most important but challenging metabolites such as GABA, Gln, and GSH. The combination of higher SNR and narrower linewidth (FWHM) results in lower CRLB for GABA, Gln, and GSH. The potential of short-echo FID spectra to detect GABA, Gln, and GSH at ultra-high field is supported also by previous findings reported in 9.4T studies (Nassirpour, Chang, & Henning, 2018a; Ziegs et al., 2023). Here, we extend the imaging of NAAG, GABA, Gln and GSH from single-slice to whole-brain and show that this is feasible at 7T which is more available for ultra-high field human imaging compared to 9.4T. As the 7T scanners have gained approval by regulatory agencies worldwide, the 7T imaging has reached clinical use and we expect ECCENTRIC will have a great contribution in clinical studies.

While correlation coefficients between the ground truth and accelerated ECCENTRIC acquisitions generally exhibit lower values for metabolic imaging (Fig. 4) compared to water imaging (Fig. 3; Supplementary Fig. S13), visual assessments indicate that the quality of metabolite mapping in the healthy brain is consistently preserved across all acceleration levels. Notably, the discrepancy between the results for metabolites and water images arises from the fact that, unlike water images where correlation coefficients are directly determined from reconstructed images, the correlation coefficients for metabolic images are influenced by additional processing steps such as water removal, fat removal, and LCModel fitting. These additional processing steps of MRSI data introduce variability that contributes to the lower correlation coefficients observed in metabolic imaging results.

The 3D ECCENTRIC FID-MRSI showed robust performance in healthy volunteers. The low variability in repeat measurements indicates high precision of metabolite quantification and significant potential for longitudinal studies to detect metabolite changes due to disease, treatment and functional tests. The high quality of the data was achieved through the use of third-order shimming, which provides more uniform $B_{0}$ field across the brain, as well as the shortened scan time, which minimized the scanner drift and possibility of subject motion. The scanner drift typically ranged from 5–10 Hz over a 10 min scan time.

ECCENTRIC encoding is highly versatile with flexible choice of FoV, spatial resolution, and spectral bandwidth that can be set to optimize SNR and acquisition time. The advantage and strength of ECCENTRIC is enabled by the possibility to freely choose the radius and position of circle trajectories in covering the k-space: 1) the free choice of circle radius allows freedom in setting FoV, spatial resolution, and spectral bandwidth without the need of temporal interleaving, 2) the free choice of circle center position allows freedom for random undersampling the k-space to accelerate acquisition by CS. This flexibility is particularly important for ^1^H-MRSI at 7T and beyond, due to the increased spectral bandwidth required which limits the duration of k-space trajectories. In addition, free choice of circle position should enable FoV with different extent along the axial dimensions for additional time saving, which cannot be achieved by concentric, rosette and spiral trajectories.

ECCENTRIC flexibility in setting FoV, resolution, and spectral bandwidth by varying the circle radius and CS undersampling to optimize SNR and acquisition time is shown in Supplementary Figures S14–S16. These results indicate that, when using the same image resolution and acquisition time, ECCENTRIC provides a higher SNR for protocols that use smaller circle radii and higher acceleration compared to protocols using larger circle radii and lower acceleration. This flexibility allows to adapt ECCENTRIC acquisition to resolutions required across a range of FoV. Although we demonstrated ECCENTRIC for human brain imaging, this method can be used for ultra-high resolution metabolic imaging of mice brain where submillimeter resolution is required for a centimeter FoV. ECCENTRIC sampling was specifically designed for MRSI SSE measurements, requiring a trajectory that is successively repeated at a high frequency. While this high-frequency repeated sampling is not a prerequisite for traditional MRI technique, certain imaging techniques may demand the rapid acquisition of successive echoes and could potentially benefit from ECCENTRIC sampling at UHF.

There are some limitations in the current implementation. In particular, reconstruction time of 3D ECCENTRIC data requires several hours. For example, using a 64×64×31 matrix size for 3.4 mm isotropic, the water removal step took 1 h, the CS-SENSE-LR reconstruction took 3 h on a GPU (or 12 h on a CPU), and the parallel LCModel fitting took 1 h on a high-performance server such as the Dell PowerEdge R7525 (with 64 cores of 2.9GHz and 128M cache, 512GB RAM, and 3 NVIDIA Ampere A40 GPUs). This computation time may be considered relatively long for routine clinical applications. Also, at the moment subject motion or scanner drift is not corrected during ECCENTRIC acquisition, which may increase the variability of metabolite quantification. The metabolite concentrations were not provided in absolute units such as millimolar but expressed in institutional units (I.U.) relative to the water reference, which still provides comparable values across subjects and scanners. We note that for absolute quantification of FID-MRSI data only the T1 relaxation correction is needed, while ultra-short (<1 ms) echo-time makes T2 relaxation negligible. Our current ECCENTRIC design restricts circle positioning to a stack-of-ECCENTRIC configuration without tilting or rotating the circles. While this simplifies reconstruction, it also limits exploration of more complex sampling schemes, such as fully tilted circles, which could enhance k-space sampling efficiency. However, implementing tilted circles in 3D k-space would pose practical challenges due to the significantly increased computational demands of a full 3D discrete Fourier transform. Nevertheless, alternative reconstruction methods, such as NUFFT-based gridding (Fessler, 2007), may offer solutions to overcome these limitations and enable more thorough exploration of sampling strategies in 3D k-space.

Artificial intelligence is reshaping the landscape of MR image reconstruction, demonstrating improved performance and enabling more efficient and sparser data acquisition (Lin et al., 2021). In the domain of MRSI, the application of deep learning for reconstruction is an evolving field, marked by notable progress in super-resolution reconstruction (Dong et al., 2022; Li et al., 2020), learned-subspace approaches (Lam et al., 2020), channel combination strategies (Motyka et al., 2021), and parallel MRSI methods (Nassirpour, Chang, & Henning, 2018b). These encouraging findings suggest that the integration of deep learning holds substantial potential to enhance reconstruction outcomes over the model-based CS-SENSE-LR reconstruction employed in this study. Such improvement may manifest in improved quality of metabolite maps or even enable higher AF, consequently leading to faster acquisition.

In summary, we have introduced ECCENTRIC an advanced acquisition-reconstruction method for MRSI that pushes the boundaries of spatial and temporal capabilities for *in vivo* metabolic imaging. Although here we specifically demonstrated ECCENTRIC for MRSI at 7T ultra-high field, this method is not limited to this field and could be used at higher (≥9.4T) and lower (3T) fields. When combined with FID-MRSI acquisition, ECCENTRIC has demonstrated outstanding performance in the comprehensive mapping of metabolites throughout the entire brain, encompassing crucial neurotransmitters such as Glu and GABA. Anticipating that ECCENTRIC will pave the way for novel advancements in neuroscience, we envision its potential to provide detailed insights into brain neurochemistry in both healthy and pathological conditions. This innovation is poised to address crucial questions and facilitate groundbreaking discoveries in both fundamental research and clinical studies.

## Supplementary Material

Supplementary Material

## Data Availability

Data can be obtained from the corresponding author (O.C.A.) upon request with institutional agreement for data sharing. 3D ECCENTRIC FID-MRSI sequence and reconstruction can be obtained from the corresponding author (A.K.) upon request for Siemens Healthcare MAGNETOM Terra / Terra.X MRI system.
