# ECCENTRIC: A fast and unrestrained approach for high-resolution in vivo metabolic imaging at ultra-high field MR

**DOI:** 10.1162/imag_a_00313

**Published:** 2024-10-15

**Authors:** Antoine Klauser, Bernhard Strasser, Wolfgang Bogner, Lukas Hingerl, Sebastien Courvoisier, Claudiu Schirda, Bruce R. Rosen, Francois Lazeyras, Ovidiu C. Andronesi

**Affiliations:** Athinoula A. Martinos Center for Biomedical Imaging, Department of Radiology, Massachusetts General Hospital, Harvard Medical School, Boston, MA, United States; Advanced Clinical Imaging Technology, Siemens Healthcare AG, Lausanne, Switzerland; Department of Radiology and Medical Informatics, University of Geneva, Geneva, Switzerland; CIBM Center for Biomedical Imaging, Geneva, Switzerland; High-Field MR Center, Department of Biomedical Imaging and Image-Guided Therapy, Medical University of Vienna, Vienna, Austria; Department of Radiology, University of Pittsburgh School of Medicine, Pittsburgh, PA, United States

**Keywords:** high resolution whole-brain metabolite imaging, 3D magnetic resonance spectroscopic imaging, ultra-high field, non-Cartesian, compressed sensing, low-rank

## Abstract

A novel method for fast and high-resolution metabolic imaging, called ECcentricCircle ENcoding TRajectorIes for Compressed sensing (ECCENTRIC), has beendeveloped at 7 Tesla MRI. ECCENTRIC is a non-Cartesian spatial-spectral encodingmethod designed to accelerate magnetic resonance spectroscopic imaging (MRSI)with high signal-to-noise at ultra-high field. The approach provides flexibleand random sampling of the Fourier space without temporal interleaving toimprove spatial response function and spectral quality. ECCENTRIC enables theimplementation of spatial-spectral MRSI with reduced gradient amplitudes andslew-rates, thereby mitigating electrical, mechanical, and thermal stress of thescanner hardware. Moreover, it exhibits robustness against timing imperfectionsand eddy-current delay. Combined with a model-based low-rank reconstruction,this approach enables simultaneous imaging of up to 14 metabolites over thewhole brain at 2–3 mm isotropic resolution in 4–10 min. MRSIECCENTRIC was performed on four healthy volunteers, yielding high-resolutionspatial mappings of neurochemical profiles within the human brain. Thisinnovative tool introduces a novel approach to neuroscience, providing newinsights into the exploration of brain activity and physiology.

## 1 Introduction

Magnetic Resonance Spectroscopic Imaging (MRSI) is a well-established molecular MRimaging modality, facilitating non-invasive exploration of*in vivo*metabolism in both human and animal models without the use of ionizing radiation. Inparticular,^1^H-MRSI can simultaneously image up to 20 brain metabolites,providing quantification of steady-state concentrations (Maudsley et al., 2020) and the dynamic change ofconcentrations under functional tasks (Bednarik etal., 2023;Mullins, 2018). Inaddition to measuring intrinsic metabolism without the need of contrast agents, MRSIcan probe metabolic enzymatic rates that are not accessible by nuclear imagingtechniques such as PET and SPECT (Davis et al.,2020). Many studies demonstrated significant value of MRSI forneuroscience (Oz et al., 2014), but theperformance of current MRSI is severely lacking behind other MRI methods, whichlimits its use and wider adoption.

Among MRI modalities, MRSI is positioned to benefit the most from ultra-high field(UHF≥7T) due to increased spectral dispersion and signal-to-noise ratio (SNR). MRSI usingvery short echo-time(≈1ms) free induction decay (FID) excitation (Boer etal., 2012;Bogner et al., 2012;Henning et al., 2009) has great potentialfor metabolite imaging due to its high SNR. Nevertheless, MRSI is hampered bysignificant limitations, including low resolution and long scan times required foracquiring the 4D(k,t) spatial-temporal space (Bogner etal., 2021). This highlights a pressing demand for acceleration strategiesin high-resolution MRSI to overcome these challenges. This is especially pertinentin the context of high-resolution whole-brain MRSI, where conventionalphase-encoding acquisition schemes would require scan time as long as several hours.Acceleration of UHF MRSI has been shown by parallel imaging such as SENSE, GRAPPA,and CAIPIRINHA with uniform undersampling (Hangel etal., 2018;Nassirpour, Chang, &Henning, 2018b;Strasser et al.,2017), or by Compressed Sensing (CS) with random undersampling (Nassirpour, Chang, Avdievitch, & Henning,2018). However, similar to MRI, these techniques, as for MRI, generallydo not allow acceleration factors (AF) above 6–10 for MRSI. Additionally,spatial-spectral encoding (SSE) techniques introduce additional prospects foraccelerating UHF MRSI as demonstrated byOtazo etal. (2006). By combining spatial-spectral encoding with undersampling,higher accelerations (AF>50) of UHF MRSI can be achieved (Maet al., 2016;Moser et al., 2019;Saucedo et al., 2021).

So far, SSE has been demonstrated at ultra-high fields using either Cartesian(echo-planar) (An et al., 2018;Nam et al., 2022;Weng et al., 2022) and non-Cartesian (spirals, rosettes,concentric circles) (Chiew et al., 2018;Esmaeili et al., 2021;Hingerl et al., 2020;Moser et al., 2019)k-spacetrajectories.

Nonetheless, integrating SSE at ultra-high field (UHF) presents formidable challengesstemming from the inverse relationship between the maximum time allocated for atrajectory repetition and the desired spectral bandwidth (SBW). This difficulty isaccentuated at higher spatial resolutions in UHF, where trajectories must coverextensive k-space while maintaining even faster repetition rates for larger SBW atUHF. Consequently, this imposes significant technical demands on the gradient systemin terms of amplitude and slew rate. The employment of temporal interleavingpresents a viable strategy to overcome this constraint, facilitating the achievementof broad spectral bandwidth and high spatial resolution for circle or spiraltrajectory (Adalsteinsson et al., 1998;Hingerl et al., 2018;Matsui et al., 1985). For echo-planar trajectory,achieving high SBW can be achieved by interlaced Fourier transform (Ebel et al., 2005) and interleaved readout gradients withalternating polarity (An et al., 2018;Posse et al., 2007). Nevertheless, theseapproaches concurrently prolong the acquisition duration and might introducespectral side-bands that degrade the signal-to-noise ratio (SNR) and interfere withmetabolite spectra. This issue arises if the number of temporal interleaves and theSBW are not optimally selected to prevent side-bands from appearing within thefrequency range of interest (Bogner et al.,2021). Additionally, dedicated hardware, such as gradient inserts, allowscircumvention of limitations on SBW (Versteeg etal., 2024).

In this study, we introduce ECCENTRIC method (ECcentric Circle ENcoding TRajectorIesfor Compressed sensing) that benefits from: 1) improved pseudo-random sampling forCS with non-Cartesian trajectories, 2) flexible sampling of the 4D (k,t) space for optimal SNR, 3) low-rank reconstruction fordimensionality reduction and denoising of data, and 4) reduced demand on thegradient system for spectral quality. Traditional trajectory designs, as mentionedabove, often struggle to achieve high-resolution SSE MRSI at UHF without usingtemporal interleaving. To address this limitation, we designed the ECCENTRICsampling pattern with smaller-sized circles. These circles, with their reducedradius, require lower gradient amplitude and slew rate compared to trajectoriesspanning the entire k-space, for identical spatial resolution and SBW. Given theupper limit in gradient amplitude and slew rate, ECCENTRIC is therefore advantageousas it allows for higher spatial resolution and/or SBW than established trajectoriesbefore reaching the gradient hardware limits, thereby avoiding the need for temporalinterleaving.

The performance of the new acquisition-reconstruction scheme was first investigatedby simulations and in a structural-metabolic phantom, and subsequently evaluatedin vivoin healthy subjects.

## 2 Theory

Circular trajectories, including rosettes and concentric circles, provide severaladvantages over spiral and echo-planar trajectories in MRSI and MRI (Adalsteinsson et al., 1998;Furuyama et al., 2012;Posse et al., 1994;Schirda et al.,2018). By design, ECCENTRIC’s circular trajectories need smallerdiameter than rosettes and concentric circles, which help achieve high-resolutionand large spectral bandwidth at ultra-high field without temporal interleaving.ECCENTRIC trajectories are produced by readout gradient wave-forms that: 1) do notneed rewinding which eliminates dead-time and the associated loss in SNR per unittime, 2) permit high matrix sizes with limited gradient amplitude, and 3) haveconstant and moderate gradient slew-rate that minimizes patient nerve stimulationand is not demanding for the gradient hardware. In contrast, MRSI SSE acquisitionsare characterized by high gradient amplitudes and slew rates, which can exacerbate$B_{0}$field drift (An et al., 2018) and induce eddycurrent artifacts (Xu et al., 2020) withecho-planar trajectories. Spiral SSE trajectories suffer from gradient imperfectionsand eddy currents, leading to trajectory distortions (Kim & Spielman, 2006). Similarly, concentric ringtrajectories experience rotation errors due to timing delays and eddy currents,although to a lesser extent (Jiang et al.,2016). Additionally, the high demand on the gradient system often leadsto temperature increases (Nam et al., 2023)and mechanical resonances (Dillinger et al.,2024).

This effect is particularly pronounced at 7T, where, for equivalent spatialresolution, the FID bandwidth is approximately doubled compared to 3T, necessitatinga doubling of the gradient slew-rate for SSE encoding.

**Fig. 1.**
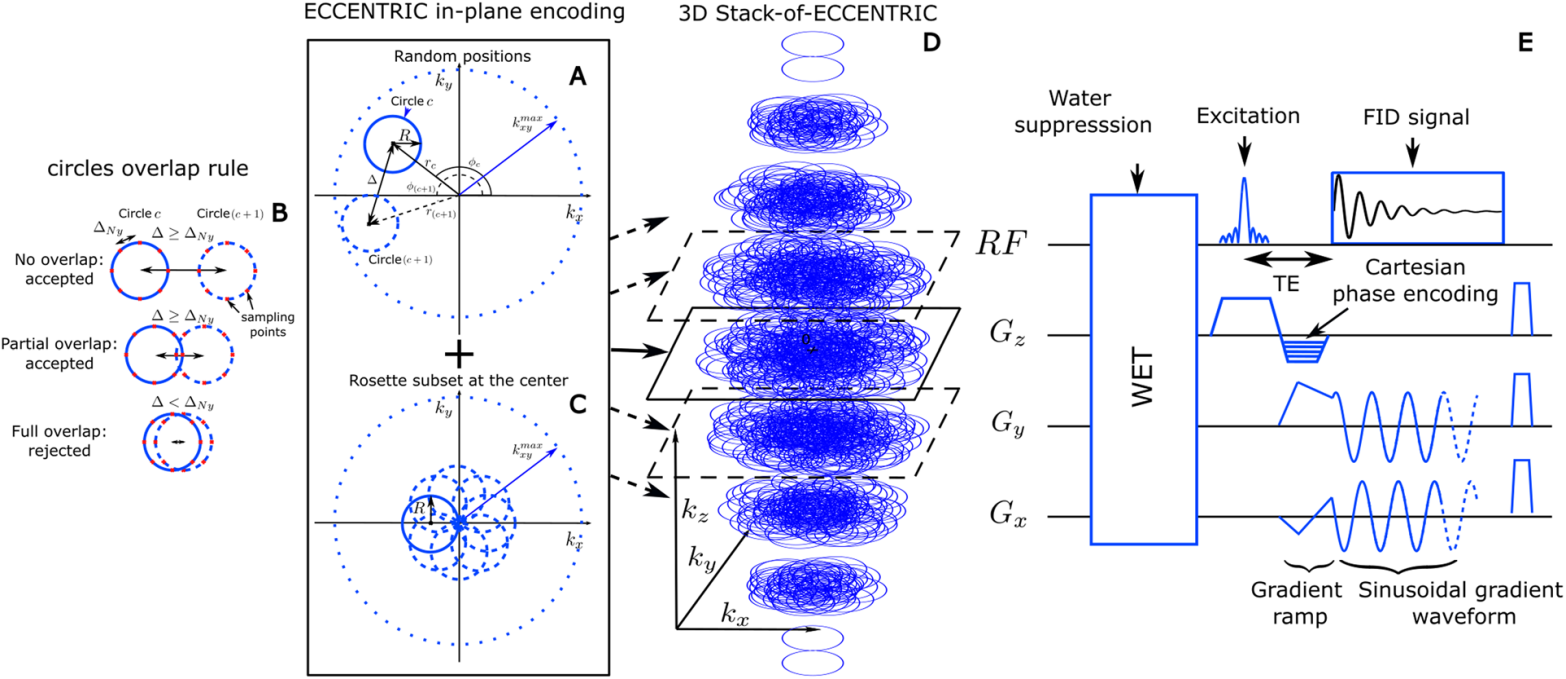
3D ECCENTRIC sampling and acquisition. (A) Circle center positions areparameterized in polar coordinates$\left(r_{c},\phi_{c}\right)$that are chosen randomly in the ranges$r_{c}\in \left[0,\text{max}\left(k_{x,y}^{max}-r,r\right)\right]$and$\phi_{c}\in \left[0,2\pi \right]$.Two consecutive circles(candc+1) must respect the overlap rule described in (B): thedistance between their respective centers,Δ,must be greater or equal to the Nyquist distance,$\Delta_{Ny}$. (C), to satisfy a systematic full sampling of thek-spacecenter, a small subset (<5%) of circles is positioned in rosette pattern in eachECCENTRIC encoding planes. (D), 3Dk-spacesampling is achieved by a stack of ECCENTRIC encoding planes with variable$k_{x,y}^{max}$to realize an ellipsoid coverage. (E) Diagram of the 3DECCENTRIC FID-MRSI sequence. First, a 4-pulse WET water suppressiontechnique is used, followed by the Shinnar–Le Roux optimizedexcitation pulse. After the excitation, the Cartesian encoding is performedalong the z-axis, simultaneously to the gradient ramp along the x- andy-axes to reach the desiredk-spaceoff-center position and velocity. Finally, a sinusoidal gradient wave-formis applied along the x- and y-axis during acquisition to produce thecircular trajectory.

Moreover, the implementation of CS acceleration (Candes et al., 2006;Donoho,2006) relies on two prerequisites. The first is that the signal or imageexhibits sparsity in a known transform domain (Knoll, Clason, et al., 2011;Michael etal., 2007). The second is that the data are randomly undersampled, whichcan be achieved by random undersampling of thek-spacein MRI applications. To enable the random sparse undersampling necessary for CS, weutilized a novel approach where successive circular trajectories are randomlypositioned ink-space,rather than using regular patterns such as rosette, concentric, or uniformlydistributed circles. The acquisition strategy of ECCENTRIC is illustrated inFigure 1. The circle centers’ polarcoordinates$\left(r_{c},\phi_{c}\right)$are chosen randomly with a uniform probability within the ranges$r_{c}\in \left[0,\text{max}\left(k_{x,y}^{max}-R,R\right)\right]$and$\phi_{c}\in \left[0,2\pi \right)$(Fig. 1A). Here,Rrepresents the circle radius,$k_{x,y}^{max}$is the largest in-planek-spacecoordinate (assuming the same spatial resolution along all axial plane directions).The majority of circles are placed randomly as shown with two successive circles(candc+1) in the sketchFigure 1A, butwith the constraint to avoid significant overlap between circles (Fig. 1B): the distance between the centers ofeach circle,Δ,must be larger or equal to the Nyquist distance (the inverse of the field-of-viewsize). WhenΔ<2R, there is partial overlap between circles; however, this redundantsampling is predominantly concentrated in the central region ofk-space,enabling an enhancement in SNR. In addition to the random pattern, a small subset ofcircles (<5%of the total number) positioned in rosette fashion is acquired inthe center ofk-space(Fig. 1C). This ensures complete samplingof the center ofk-space,which is beneficial for SNR and reconstruction performance (Knoll, Clason, et al., 2011;Michael et al., 2007) with negligible effect onacquisition time. The homogeneous random distribution of circle polar coordinatesresults intrinsically in a pseudo-randomk-spacesampling with density following1/∥k∥outside of the rosette sampled central region.

The ECCENTRIC design offers the flexibility to choose the radius of circles withinthe range of0to$\frac{k_{x,y}^{max}}{2}$.Therefore, for a given matrix size, field-of-view (FoV), and spectral bandwidth, theradius of ECCENTRIC circles can be chosen to ensure that, even without temporalinterleaving, the limits of gradient hardware’s slew rate and amplitude arenot exceeded. As detailed below, ECCENTRIC sampling fulfills better the randomundersampling required by CS compared to echo-planar (Hu et al., 2008,^2010^;Iqbal et al., 2016;Kampf et al., 2010;Otazo et al., 2009;Santos-Díaz & Noseworthy, 2019), spiral (Chatnuntawech et al., 2015), and radial (Saucedo et al., 2021) trajectories.

InFigure 2, a comparison is made betweenECCENTRIC, uniform distributed circles trajectory, concentric circles, and rosettesampling. The trajectory and sampling density in thek-spacefor each pattern and acceleration factor highlight the differences in samplingdistribution. While rosette and concentric circle trajectories provide high samplingdensity at the center ofk-space,uniformly distributed circles exhibit a flat and less optimal sampling density interms of SNR. Accurate reconstruction benefits from sampling the center ofk-space,which contains data with the highest SNR. The density distribution of ECCENTRIC liesbetween that of rosette and concentric circles and uniform distribution. It featuresa high-density singularity at thek-spacecenter which gradually decreases towards the periphery ofk-space.

**Fig. 2.**
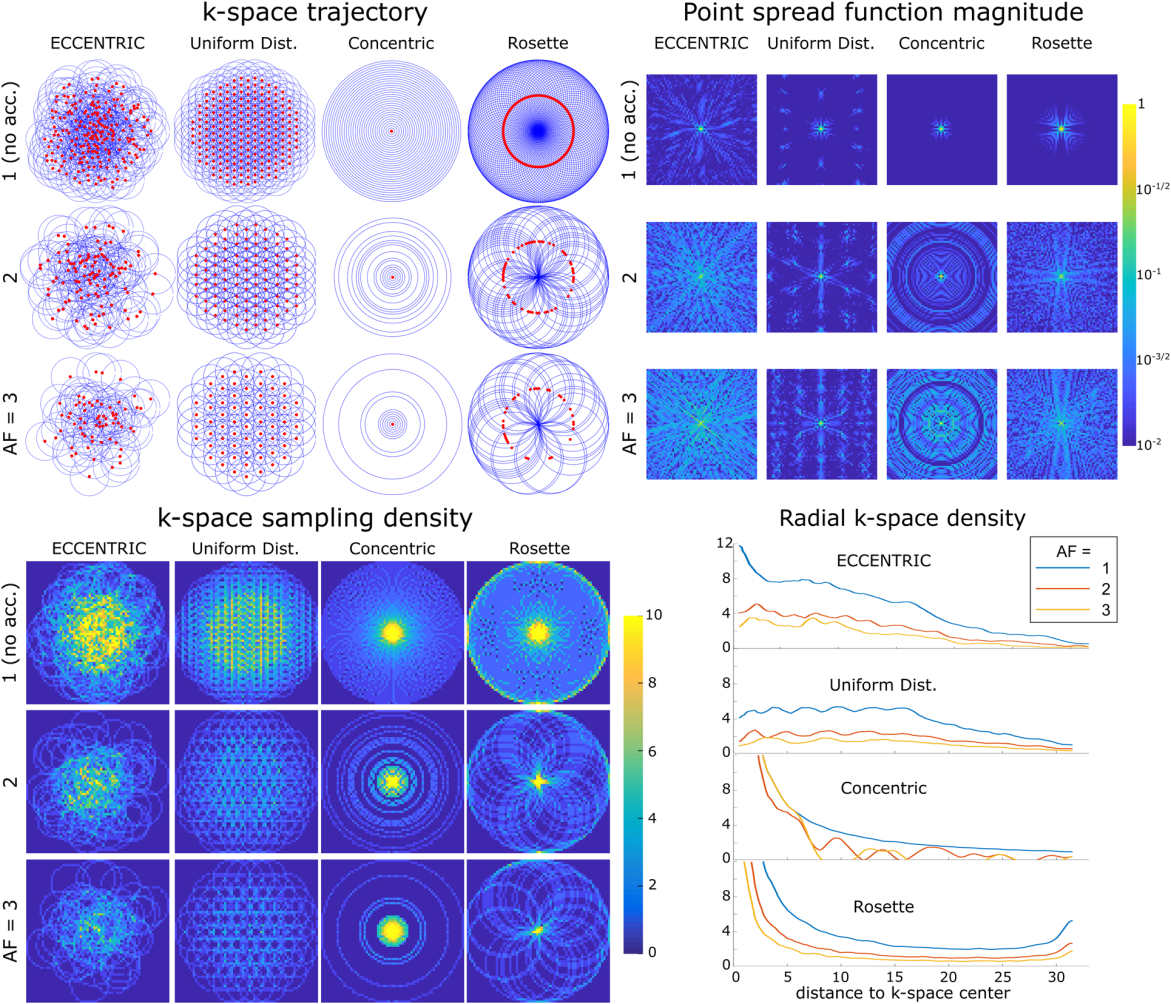
Comparative analysis of circulark-spaceencodings. Top left,*k*-space trajectories for ECCENTRIC,uniform distributed circles, concentric circles, and rosette trajectoriesfor a64×64encoding matrix. Red dots indicate the circle centerpositions. The acceleration factors AF =1,2,3correspond to ECCENTRIC and uniform distributed circlestrajectories with202,101, and67circles respectively;31,16, and11concentric circles;101,51, and34Rosette circles. Top right, the point spread function(PSF) calculated for each trajectory and acceleration on a log-scalehighlight the presence of incoherent and coherent aliasing patterns. Bottom,the sampling density for the same trajectories and AFs, represented in the2Dk-space(left) and along a radial projection (right).

The point spread function (PSF) simulations inFigure2illustrate that ECCENTRIC is inherently suited for the randomundersampling necessary to achieve optimal CS performance (Michael et al., 2007). The PSF reflects the interferencebetween voxels in image space resulting from undersampling. Simulations reveal anincoherent pattern for PSF of ECCENTRIC due to the pseudo randomk-spacesampling, and the PSF pattern spreads with the increasing acceleration factor butconserve the pseudo-random behavior with undersampling (Fig. 2). In comparison, uniform distributed circles, concentriccircles, and rosette trajectories have more compact PSFs for fully sampledacquisitions and their PSFs exhibit coherent patterns when undersampling is applied,which is less favorable for CS acceleration. The PSF was computed on a64×64matrix and obtained from a single point source (Mareci & Brooker, 1991) that was encoded ink-spaceand then reconstructed with Non-uniform Fourier transform with Voronoi’spartition density compensation (Rasche et al.,1999).

To extend ECCENTRIC to 3Dk-spacesampling, a stack of ECCENTRIC is employed with circles randomly placed in the$k_{x}$-$k_{y}$planes, while$k_{z}$is encoded using Cartesian phase-encoding (Fig.1D). The 3Dk-spacecan be covered using spherical or ellipsoid coverage, where the in-planek-spaceboundary is defined as$k_{x,y}^{max}=\frac{n}{2FoV}\sqrt{1-{\left(k_{z}/k_{z}^{max}\right)}^{2}}$,withFoVrepresenting the FoV size andnthe spatial resolution. The spherical k-space coverage yields an additionalacceleration factor of 1.5 compared to cylindrical k-space coverage. To achievecomplete sampling in a single$k_{x}$-$k_{y}$plane, the number of needed ECCENTRIC circles can be derived similar to rosetteencoding that requires$\frac{\pi n}{2}$circles (Schirda et al., 2009). Expandingfrom the number of points of a fully sampled rosette trajectory to ellipsoidalcoverage, the total number of ECCENTRIC circles required for complete sampling of asingle partition of the stack is$\frac{\pi nk_{x,y}^{max}}{2R}$. To achieve circle encoding with off-center position$\left(r_{c},\phi_{c}\right)$,a brief gradient ramp is used to gain an initial momentum$\left(k_{x},k_{y}\right)$position and the necessary velocity. This process is done at thesame time as the slab excitation rewinder overlapped byz-phaseencoding and does not increase the echo time, as shown inFigure 1E. In implementing CS acceleration, the total number ofECCENTRIC circles$N_{c}$is reduced uniformly across the stacks by a factor of AF. Since each circle patternfor every partition of the ECCENTRIC stack is randomly drawn, this results in sparseand random sampling across all three dimensions of the k-space.

Due to the non-uniformity and sparsity of the sampling, a specific model is necessaryto reconstruct 4D (k,t) data of ECCENTRIC into image-frequency space. In previous studies(Klauser et al., 2019,^2021^,^2022^), wedemonstrated the effectiveness of CS-SENSE-LR model that combinespartial-separability (or low-rank) with Total-Generalized-Variation (TGV) constraintfor reconstructing Cartesiank-spacedata acquired with random undersampling, leading to improved SNR. Here, we extendedthe CS-SENSE-LR approach to incorporate non-uniform Fourier sampling necessary forreconstructing ECCENTRIC data. Defining the discrete MRSI data in image space to beρas an$N_{\text{r}}$byTarray(with$N_{\text{r}}$the number of spatial points andTthenumber of sampling time points), the low-rank hypothesis on the magnetizationassumes that the MRSI data can be separated into a small number of spatial andtemporal components:

ρ=UV (1)

whereUis a$N_{\text{r}}$byKarrayandVaKbyTarray,withKtherank of the low-rank model. These components are retrieved by CS-SENSE-LRreconstruction solving the inverse problem

$$\begin{array}{cccc} \text{arg}\underset{\text{U},\text{V},\text{L}}{\text{min}} & {\left\|\;W\left(\text{s}-ℱCℬ\left(\text{UV}+\text{L}\right)\right)\;\right\|}_{2}^{2} & & \\ & +\lambda \sum_{c=1}^{K}{\text{TGV}}^{2}\left\{U_{c}\right\}. & & \end{array}$$ (2)

wheresthe measured data,ℱthe non-uniform Fourier transform (NUFT) encoding operator,Cthe coil sensitivity operator,ℬthe$B_{0}$frequency shift operator, andLrepresentthe lipid signal ($N_{\text{r}}$byTarray)from skull that is reconstructed simultaneously with brain metaboliteUandVbut on a separate spatialsupport.Wis a weighting operator of a Hammingwindow shape, and decreasing with the distance to the center of thek-space(Ban et al., 2019;Klauser et al., 2021). The NUFT encoding operator ofECCENTRICℱis a discrete non-uniform Fourier transform of type 1:

$${\left(ℱ\rho \right)}_{j,t}=\underset{i}{\sum }{\text{e}}^{\text{i}2\pi {\text{k}}_{j}\;\cdot \;{\text{r}}_{i}}\rho_{i,t}$$ (3)

with${\text{r}}_{i}$are the uniform image space coordinates and${\text{k}}_{j}$k-spacesampling-point coordinates located on the 3D ECCENTRIC circles.

${\text{TGV}}^{2}$is the total generalized variation cost function withλthe regularization parameter (Knoll, Bredies, etal., 2011). The regularization parameter used in the reconstruction wasadjusted to$\lambda =3\times 10^{-4}$by gradually increasing it from a low value until the noise-likeartifacts in the metabolite maps disappeared (Klauser et al., 2022;Knoll, Bredies, etal., 2011). The reconstruction rank,K,was determined qualitatively as the minimum number of components that contain somesignal distinguishable from noise. For the 3D ECCENTRIC reconstruction,Kwas specifically set to 40. Additional information regarding the determination ofKand an illustration of its impact on the results are available in the SupplementaryMaterial and depicted inFiguresS9–S11.

The contamination ofUandVby skull-lipid signal isprevented by filtering of the gradient descent during the reconstruction (Eq. 2). Lipid signal is removed fromeach step of the gradient descent by applying the operator(1−ℙ)withℙthe lipid subspace projection computed from the estimated lipid signal at the skullL(Klauser et al., 2019). The reconstruction algorithm forUandV, along with the detailedutilization of the lipid suppression operator(1−ℙ),is further described in theSupplementary Material, accompanied by pseudocode for clarity.

## 3 Methods

### 3.1 ECCENTRIC FID-MRSI acquisition parameters

^1^H-FID-MRSI (Bogner et al.,2012;Henning et al., 2009)acquisition was implemented with 3D spherical stack-of-ECCENTRIC sampling asdepicted in (Fig. 1E) on a 7T scanner(MAGNETOM Terra, Siemens Healthcare, Erlangen, Germany) running VE12U SP1software and equipped with NOVA head coils (32Rx/1Tx and 32Rx/8Tx). Theecho-time (TE) was set to the minimum possible:0.9ms with a27degree excitation flip-angle (FA) and275ms repetition-time (TR). A slab-selective excitation wasperformed with a Shinnar-LeRoux optimized pulse (Klauser et al., 2021;Pauly et al.,1991) with 6.5 kHz bandwidth and was preceded by four-pulses WETwater suppression scheme (Klauser et al.,2021;Ogg et al., 1994) (Fig. 1). The FoV was220×220×105mm^3^(A-P/R-L/H-F) with85mm-thick excited slab. A voxel size of3.4×3.4×3.4mm^3^(40.5μl) was realized with a64×64×31matrix. The ECCENTRIC circles radiusRwas set to$1/8\frac{n}{FoV}$which corresponds to a diameter that encompasses a quarter ofthe width of thek-space,withnbeing the square matrix size. The radiusRwas selected to maximize the number of sampled points per circle, therebyoptimizing SSE acceleration, while ensuring not to exceed the gradient systemlimits for the desired spectral bandwidth. With$R=1/8\frac{n}{FoV}$, ECCENTRIC enables a spectral bandwidth of 2,280 Hz withoutthe need for temporal interleaving for in-plane FoV = 220 x 220$mm^{2}$and64×64matrix. The effect of circle radius on the SNR and the qualityof metabolic maps was investigated inSupplementary Figures S14–S16. With this radius,ECCENTRIC circles cover 20 points in k-space, inherently providing an SSEacceleration of 20 without undersampling. The FID was sampled with 500time-points which corresponds to the number of revolutions on each ECCENTRICcircle, and resulted in a total FID duration of 220 ms. To obtain the fullysampled (AF = 1) spherical 3D stack-of-ECCENTRIC with these parameters, atotal number of$N_{c}=4,072$circles is required, which corresponds to18min40sec acquisition time (TA). For accelerated acquisitions, wedecreased the number of circles to$N_{c}/AF$, with TA being shortened proportionally. For instance, withthe same encoding parameters, AF = 2 needs$N_{c}=2,036$in 9 min 20 sec, AF = 3 needs$N_{c}=1,357$in 6 min 16 sec, AF = 4 needs$N_{c}=1,018$in 4 min 40 sec, and so on.

A rapid calibration scan of water reference data was performed by turning offwater suppression and using the same FoV, FA slab excitation, TR, FID duration,and spectral bandwidth, but with rosette trajectory sampling at lower resolution (23×23×19) in1min16sec.

The$B_{0}$shimming of the 85 mm thick whole-brain slab was performed using themanufacturer methods that adjusted the shim currents over 12 spherical harmonicscoils: three 1st order, five 2nd order, and four 3rd order. The global linewidthof the water over the entire 85 mm slab was between 25–42 Hz across allsubjects. In the majority of the subjects the global water linewidth was between30–35 Hz. Adjustment of the$B_{1}+$transmit and water suppression was subsequently performed withmanufacturer methods. The entire adjustment procedure took between 1–2min for every subject.

### 3.2 Reconstruction of ECCENTRIC FID-MRSI metabolic images

The rapid water reference data were used to compute the coil sensitivity mapsusing ESPIRiT (Uecker et al., 2014)(Coperator inEq. 2) and to estimate a$\Delta {\text{B}}_{0}$field map with*multiple signal classification algorithm*(MUSIC)(Gruber & Hayes, 1997). Thefield correction operator inEquation2was then determined by$ℬ={\text{e}}^{\text{i}2\pi t\gamma \Delta {\text{B}}_{0}}$,where$\gamma \Delta {\text{B}}_{0}$is the spatial frequency shift caused by the field inhomogeneity map (in Hz). Toreconstruct 3D ECCENTRIC FID-MRSI data and obtain the metabolic images, weemployed a comprehensive pipeline that included: 1) water removal using the HSVDmethod (Barkhuijsen et al., 1987) for eachcoil channel (Klauser et al., 2019), 2)determination of the$\Delta {\text{B}}_{0}$field map and the coil sensitivity maps from the rapid water reference data, 3)CS-SENSE-LR reconstruction model fromEquation 2, which includes simultaneous suppression of scalp lipidsignals, and 4) spectral fitting by LCModel software (Provencher, 1993) with the reconstructed rapid waterreference data serving as the normative signal. Because FID gradient-echoexcitation does not refocus chemical shift evolution during echo-time thespectra need first order phase correction, which was performed by backwardlinear prediction of the evolution (Nassirpour,Chang, & Henning, 2018a). A metabolite basis obtained byquantum mechanics simulations in GAMMA (Smith etal., 1994) was utilized to fit and quantify 21 metabolites:phosphocholine (PCh), glycerophosphocholine (GPC), creatine (Cr),phosphocreatine (PCr), gamma-aminobutyric acid (GABA), glutamate (Glu),glutamine (Gln), glycine (Gly), glutathione (GSH), myo-inositol (Ins),N-acetylaspartate (NAA), N-acetyl aspartylglutamate (NAAG), scyllo-inositol(Sci), lactate (Lac), threonine (Thr), beta-glucose (bGlu), alanine (Ala),aspartate (Asp), ascorbate (Asc), serine (Ser), and taurine (Tau).Phosphorylcholine and glycerophosphorylcholine were combined into totalcholine-containing compounds (Cho), while creatine and phosphocreatine werecombined into total creatine (tCre). Concentration maps were then generated forthe metabolites included in the simulated basis.

The water reference signal was used as quantification reference by LCModel, andthe resulting concentration estimates were expressed in institutional units(I.U.). This allowed for comparisons of metabolite levels across both subjectsand different metabolites. The ultra-short TE used in the ECCENTRIC MRSI dataacquisition meant that$T_{2}$relaxation correction was unnecessary for both metabolite and water signals. Asa result of employing a short TR with Ernst flip angle, metabolite maps maycontain$T_{1}$-weightedcontrast. Correcting for this would necessitate measuring$B_{1}+$field and incorporating prior knowledge of metabolite$T_{1}$.The results of the LCModel fitting for each voxel were further used to generatespatial maps of the concentration of each metabolite. To assess the quality ofthe MRSI data and the goodness of fit, quality control maps of Cramer-Rao lowerbounds (CRLB), line-width (FWHM), and SNR were generated from the LCModelfitting.

### 3.3 ECCENTRIC FID-MRSI in high-resolution phantom

Experimental performance of ECCENTRIC sampling was first tested on ahigh-resolution structural-metabolic phantom. We used a custom-made phantom withgeometry similar to Derenzo molecular imaging phantom (Derenzo et al., 1977) containing 5 sets of tubes withdiameters of2,4,6,8and10mm as shown inFigure 3.Each set contained 6 tubes of identical diameter separated by a distance equalto twice the inner diameter positioned in a triangular configuration. In everyset, the six tubes were filled with metabolite solutions containing 10 mM ofcreatine. Magnevist (Gd-DTPA) was added (1 mL/L) in each tube to shorten$T_{1}$and create$T_{1}$-weightedcontrast for structural MRI. The whole tube structure was inserted in a largecylindrical container (13.33 cm inner diameter) which was filled with 10 mM NaClsolution. Further details of phantom manufacturing and chemical composition arementioned inKlauser et al. (2021).

**Fig. 3.**
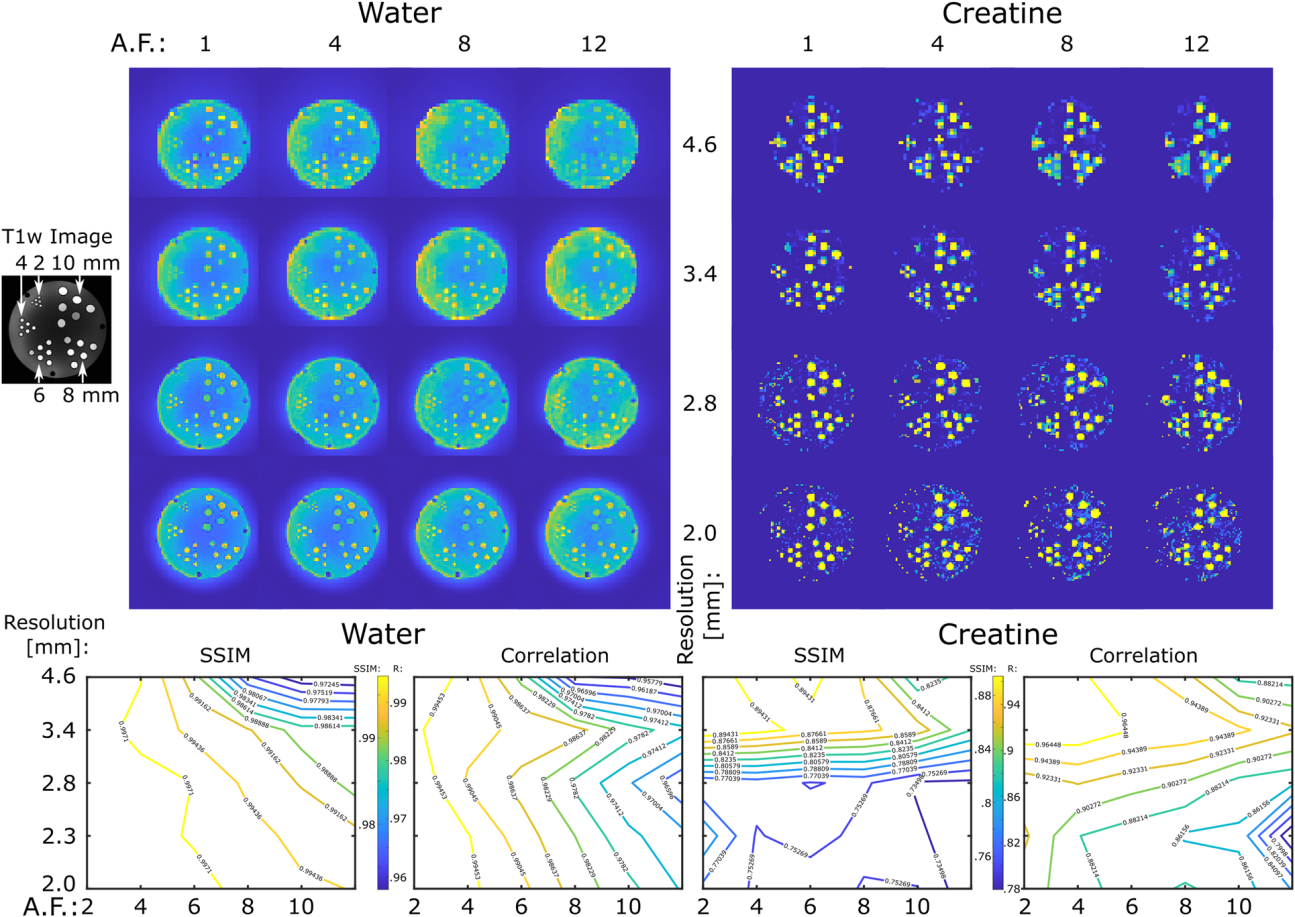
ECCENTRIC imaging of water and metabolites in the high-resolutionstructural-metabolic phantom. ECCENTRIC performance was tested forspatial resolutions of 4.6, 3.4, 2.8, 2.0 mm and acceleration factorsbetween 1–12. Top, examples of water and creatine images areshown for all 4 resolutions and 4 acceleration (AF: 1,4,8,12). Bottom,SSIM and correlation factors for each resolution and acceleration arecalculated considering as ground truth the fully sampled image (AF= 1).

Due to the phantom’s geometric structure being present only in the axialsection, we opted for a 2D ECCENTRIC acquisition. The sampling scheme for 2DECCENTRIC is identical to the centralk-spacepartition used in 3D ECCENTRIC. The 2D ECCENTRIC acquisition maintained the sameRF-pulse and FA but required slightly longer TE to1.15ms, and the TR was set to450ms to accommodate the longer$T_{1}$relaxation times in the phantom. The FID was measured with a spectral bandwidthof2,000Hz over350ms, and successive acquisitions were performed with increasedin-plane resolution. The circle radius(R)was set to$\frac{n}{8FoV}$,$\frac{n}{8FoV}$,$\frac{n}{9FoV}$, and$\frac{n}{10FoV}$for4.6,3.4,2.8, and2.0mm in-plane resolutions, respectively, to avoid temporalinterleaving for any spatial resolution.

In addition to metabolite imaging, we conducted water imaging of the structuralphantom at identical spatial resolutions to more comprehensively evaluate thespatial encoding performance of ECCENTRIC. To accomplish this, we employed the2D ECCENTRIC sequence without water suppression, utilizing a short TR of100ms and an FA of40degrees. This choice was made to maximize the$T_{1}$-weighted($T_{1}$w)contrast of the tubes within the cylindrical phantom. Subsequently, the firstpoint of the acquired timeseries was reconstructed to generate the$T_{1}$-weightedwater image. Both metabolite and water data were acquired with fully sampledECCENTRIC. To investigate accelerations, the fully sampled ECCENTRIC data wereretrospectively undersampled for acceleration factors (AF) between2−12.

The effect of the acceleration on water and metabolite imaging was evaluated byanalyzing the structural similarity index (SSIM) and correlation coefficient forall voxels inside the phantom with respect to the fully sampled data (Fig. 3).

### 3.4 ECCENTRIC FID-MRSI in healthy volunteers

Five healthy volunteers were scanned at Athinoula A. Martinos Center ForBiomedical Imaging for this study. The protocol was approved by theinstitutional ethics committee, and written informed consent was given by allsubjects before participation. The 3D ECCENTRIC FID-MRSI sequence describedabove was acquired with voxel size of3.4mm isotropic at AF = 1,2,3 and 4 successively. In twovolunteers, the performance of 3D ECCENTRIC FID-MRSI was also tested atultra-high resolution with voxel size of2.5mm isotropic (matrix88×88×43, AF = 4, TA = 10 min 26 sec) and compared to3.4mm isotropic (matrix64×64×31, AF = 2, TA = 9 min 20 sec). Our objective wasto determine the feasibility of achieving a metabolic imaging protocol thatdelivers close to 3 mm isotropic whole-brain coverage in under 10 min usingECCENTRIC.

All volunteers were scanned with a$T_{1}$-weightedanatomical MP2RAGE sequence (Marques et al.,2010) (1 mm isotropic, 4,300 ms TR, 840 ms and 2,370 ms TI) forpositioning of the MRSI FoV and for the generation of skull-masks that areneeded for the lipid removal during the reconstruction and to exclude voxelslocated outside the head volume.

### 3.5 Quantitative analysis

For quantitative analysis of 3D ECCENTRIC FID-MRSI, the metabolite concentrations(I.U.) were analyzed in each brain lobe and tissue type. This involvedsegmenting MP2RAGE images into gray matter, white matter, and cerebrospinalfluid using Freesurfer version 7.1.1 software (Fischl et al., 2002). Cerebral lobes were then identified utilizinga standard atlas, and a general linear model was employed to estimate metaboliteconcentrations within each atlas-defined structure (Klauser et al., 2022).

### 3.6 Reproducibility analysis

The reproducibility of metabolite maps measured by 3D ECCENTRIC FID-MRSI wereassessed by repeated imaging in four healthy volunteers. All scans wereconducted sequentially without repositioning the volunteer. For thereproducibility analysis, three data sets withAF=3in each volunteer were compared. One data set was acquiredwithAF=3, and the other two datasets were obtained by retrospectiveundersampling toAF=3the data acquired withAF=1andAF=2. Coefficients-of-variation (COV) were computed for individualanatomical regions from the three data sets. Both inter-measurement andinter-subject COVs were calculated and then averaged across subjects.

## 4 Results

### 4.1 ECCENTRIC imaging in high-resolution phantom

In the series of water imaging experiments, ECCENTRIC can resolve the structuraldetails of the phantom up to the resolution targeted by the imaging protocol ascan be seen inFigure 3.

For water images, no visible difference in image quality can be seen forretrospective accelerations factors up to AF = 4, minor changes can bedetected for AF between 4–8, and moderate loss of details for AF between8–12 when compared to the fully sampled acquisition (AF = 1).Considering AF = 1 as ground truth, quantitative analysis reveals thatSSIM≥0.99 across all resolutions for accelerations up to AF = 4, and SSIMdecreases to 0.97 for the highest acceleration and resolution tested. Similarly,correlation factors larger than 0.99 are observed up AF = 4, whichdecrease to 0.95 for the lowest resolution and largest acceleration factor.Comparing the different spatial resolutions, the higher CS accelerations showbetter performance for higher resolution images.

In the series of metabolite imaging experiments, ECCENTRIC was used to image thecreatine metabolite present in the tubes with the same resolutions andacceleration factors as in the water imaging (Fig.3). These results of these experiments show that: 1) metabolite mapsexhibit comparable quality for AF between 1–4, 2) forAF>4there is reduction in image details and an elevation of thenoise level. Considering AF = 1 as ground truth, across the entire seriesof measurements SSIM range between 0.75–0.89, and correlation factorsbetween 0.79–0.96. Unlike the water imaging results, the increasingacceleration does not yield higher SSIM and correlation factors as the spatialresolution increases. This discrepancy is likely attributed to the much lower ($10^{3}-10^{4}$less) SNR of metabolites in comparison to water, which becomes critical for thesmallest voxel size. In particular, we note that for isotropic voxel size of 3.4mm and for acceleration factors up to 4 we obtained the highest SSIM andcorrelation factors for metabolic imaging.

### 4.2 ECCENTRIC FID-MRSI in healthy volunteers

Examples of metabolic images for seven metabolites obtained with retrospectiveAF=1−4are shown inFigure 4.Very similar structural details and tissue contrast of metabolic images areobtained for all accelerations compared to the fully sampled data. This isvisible also by inspecting spectra that show the same metabolic profile acrossacceleration factors (AF=1−4). The CS accelerations (AF=2,3,4) were achieved by retrospectively undersampling the fullyacquired data (AF=1). The purpose of this was to focus the analysis on the effectsof CS acceleration, while avoiding any image differences that could be caused byhead motion during different acquisitions. To illustrate the effect of eachcomponent of the CS-SENSE-LR reconstruction, we modified the model to isolateand evaluate each feature independently. The results are presented inSupplementary Material Section II.B andFigure S8, and are consistent with previously publisheddata obtained using similar reconstruction models for Cartesian CS FID-MRSI (seesupplementarymaterialinKlauser et al.(2021)). To validate the low-resolution rapid water referencemeasurement described inSection 3.1andused for the reconstruction and quantification, we compared it inSupplementary FigureS12with a water 3D ECCENTRIC FID-MRSI reference acquisition, whichmatched the spatial resolution of the metabolite measurement.

**Fig. 4.**
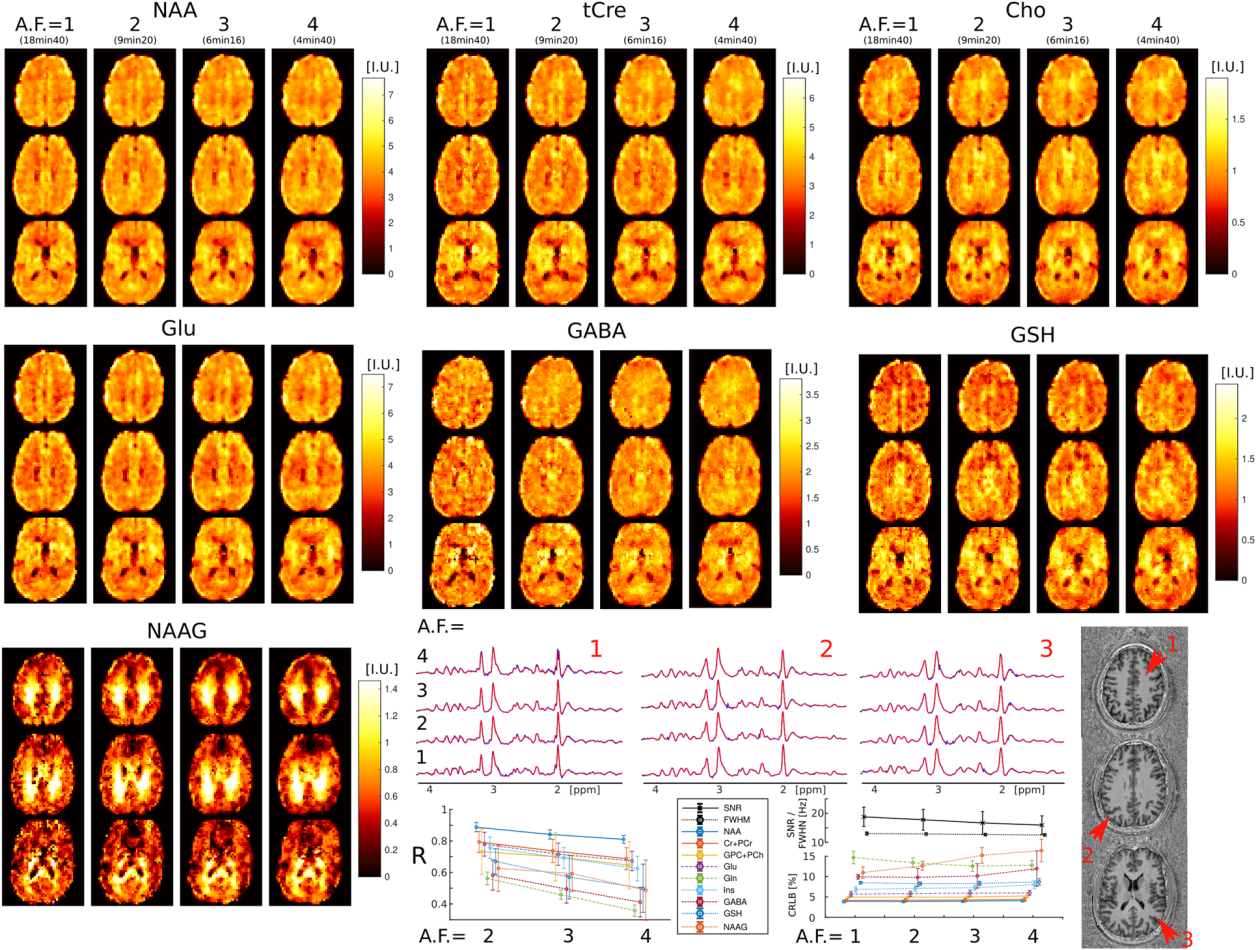
3D ECCENTRIC FID-MRSI metabolic images of human brain acquired in ahealthy volunteer with3.4mm isotropic voxel size and CS acceleration factors AF= 1–4. Top, metabolite maps of seven relevant brainmetabolites (NAA, tCre, Cho, Glu, GABA, GSH, and NAAG) are shown for allacceleration factors (AF). Spectra from three brain locations indicatedby red arrows on the anatomical image. At the bottom, the left plotdisplays the correlation coefficients between accelerated images (AF=2,3,4) and fully sampled images (AF=1), while the right plot shows the LCModelquantification error (CRLB), linewidth (FWHM), and SNR.

Visual inspection of metabolic images reveal that: 1) tCre, Glu, and GABA havelarger signal in gray matter than white matter, 2) NAA has more signal in graythan white matter, but with lower gray-white matter contrast compared to tCre,Glu, and GABA, 3) Cho has higher signal in frontal white matter than graymatter, and 4) NAAG has the largest contrast from all metabolites, with muchlarger signal in white matter compared to gray matter. Metabolic images obtainedwithAF=1andAF=2are largely identical. Minor blurring of fine structuraldetails starts to become noticeable forAF≥3, however adequate delineation of gray-white matter folding ismaintained up toAF=4. Additionally, the performance of ECCENTRIC to reveal brainstructural details was probed also by water imaging in four healthy volunteers.Brain water imaging by ECCENTRIC shows comparable performance forAFbetween 1–10 (Supplementary Fig. S13), similar to the phantomresults.

Quantitative image analysis shows that the correlation between the acceleratedand fully sampled metabolic images is high (R>0.7) for metabolites that have a high SNR (>10) such as NAA, Cho, tCre, Glu, and Ins, while metabolites oflower SNR such as Gln, GABA, GSH, and NAAG exhibit lower correlations (R=0.4−0.7). The error (CRLB) of spectral fitting is below20%,which indicates very high goodness-of-fit by LCModel software. The CRLB does notdegrade with the acceleration factor, except for NAAG and GABA, although even inthis case it does not exceed the20%limit. The SNR shows only a minor decrease betweenAF=1(SNR = 17) andAF=4(SNR = 15), while the linewidth does not degrade withacceleration.

### 4.3 Quantitative analysis of ECCENTRIC metabolic imaging

Table 1presents brain regionalconcentrations of nine metabolites quantified using water signal as referenceand expressed in institutional units (I.U.). The concentrations were calculatedin the gray and white matter of the five major brain lobes across the fivehealthy volunteers. Our results indicate that: 1) six metabolites have higherconcentrations in gray-matter compared to white matter (GM/WM = 1.19tCre, 1.12 NAA, 1.2 Glu, 1.34 Gln, 1.15 GABA, 1.11 GSH); 2) two metabolites havehigher concentrations in white-matter compared to gray-matter (GM/WM =0.96 Cho, 0.47 NAAG); and 3) one metabolite has region-dependentgray/white-matter ratio (GM/WM = 1.17–0.87 Ins). The largestgray-white matter contrast is exhibited by NAAG due to its specificcompartmentalization in white matter.

**Table 1.** Metabolite concentrations (I.U.) and quantification error(Cramér-Rao lower bound, CRLB %) in each brain lobe and tissuetype.

Mean across volunteers	Frontal		Limbic		Parietal		Occipital		Temporal		Usable voxels
(Standard deviation)	WM	GM	WM	GM	WM	GM	WM	GM	WM	GM	Mean % (std)
tCre [I.U.]	3.34 (0.34)	3.84 (0.04)	3.22 (0.35)	4.46 (0.28)	3.30 (0.28)	3.95 (0.28)	3.31 (0.30)	3.39 (0.19)	3.09 (0.25)	3.81 (0.19)	
tCre CRLB [%]	3.25 (0.24)	3.54 (0.41)	3.38 (0.31)	3.18 (0.30)	3.21 (0.33)	3.26 (0.26)	3.45 (0.62)	3.64 (0.62)	3.42 (0.37)	3.33 (0.26)	73.3 (3.3)
NAA [I.U.]	4.16 (0.98)	4.51 (0.66)	4.07 (1.01)	5.38 (0.90)	4.31 (0.96)	4.84 (0.88)	4.33 (0.98)	4.18 (0.85)	3.91 (0.84)	4.35 (0.73)	
NAA CRLB [%]	3.13 (0.34)	3.55 (0.64)	3.26 (0.33)	3.07 (0.36)	2.86 (0.26)	3.05 (0.37)	3.30 (0.54)	3.63 (0.64)	3.32 (0.39)	3.32 (0.34)	73.0 (3.5)
Ins [I.U.]	3.49 (0.52)	3.51 (0.35)	3.65 (0.60)	4.28 (0.48)	3.73 (0.66)	3.58 (0.29)	3.74 (0.65)	3.27 (0.57)	3.56 (0.65)	3.39 (0.51)	
Ins CRLB [%]	5.33 (0.64)	6.04 (0.94)	5.29 (0.52)	5.36 (0.42)	5.16 (0.42)	5.63 (0.52)	5.38 (0.48)	5.84 (0.50)	5.34 (0.33)	5.68 (0.40)	73.0 (3.5)
GPC+PCh [I.U.]	1.03 (0.10)	0.93 (0.05)	1.10 (0.10)	1.24 (0.08)	1.06 (0.11)	0.95 (0.09)	0.94 (0.12)	0.79 (0.06)	0.99 (0.07)	0.98 (0.12)	
GPC+PCh CRLB [%]	3.63 (0.34)	4.24 (0.59)	3.46 (0.31)	3.56 (0.27)	3.56 (0.29)	3.97 (0.33)	4.13 (0.57)	4.59 (0.64)	3.68 (0.29)	3.94 (0.25)	73.5 (3.3)
Glu [I.U.]	3.86 (0.53)	4.50 (0.29)	3.72 (0.49)	5.24 (0.46)	3.86 (0.39)	4.61 (0.39)	3.80 (0.36)	3.82 (0.38)	3.50 (0.35)	4.29 (0.44)	
Glu CRLB [%]	4.51 (0.51)	5.01 (0.85)	4.72 (0.59)	4.37 (0.47)	4.31 (0.53)	4.48 (0.55)	4.82 (1.14)	5.22 (1.32)	4.80 (0.74)	4.71 (0.64)	72.8 (3.8)
Gln [I.U.]	0.89 (0.06)	1.24 (0.25)	0.83 (0.06)	1.33 (0.16)	0.80 (0.16)	1.08 (0.26)	0.87 (0.22)	0.91 (0.21)	0.80 (0.09)	1.07 (0.22)	
Gln CRLB [%]	15.93 (2.45)	14.81 (2.22)	17.72 (2.68)	14.38 (2.48)	17.92 (4.78)	16.22 (3.24)	18.99 (5.14)	18.77 (4.11)	18.30 (2.95)	16.45 (2.60)	56.3 (4.6)
GABA [I.U.]	1.34 (0.09)	1.48 (0.29)	1.34 (0.10)	1.79 (0.37)	1.37 (0.14)	1.58 (0.30)	1.33 (0.18)	1.37 (0.18)	1.21 (0.16)	1.39 (0.32)	
GABA CRLB [%]	8.68 (1.10)	9.61 (1.51)	8.86 (1.50)	8.52 (1.74)	8.54 (2.08)	8.78 (1.76)	9.48 (2.34)	9.90 (2.22)	9.60 (2.16)	9.52 (2.09)	68.3 (1.5)
GSH [I.U.]	1.07 (0.24)	1.22 (0.05)	1.11 (0.26)	1.45 (0.20)	1.10 (0.20)	1.17 (0.07)	1.05 (0.18)	1.00 (0.09)	1.02 (0.17)	1.14 (0.12)	
GSH CRLB [%]	8.20 (0.88)	8.77 (1.15)	8.29 (0.93)	7.96 (0.79)	8.04 (1.00)	8.43 (0.87)	9.12 (2.01)	9.80 (1.90)	8.65 (1.23)	8.81 (1.17)	71.5 (3.1)
NAAG [I.U.]	0.82 (0.14)	0.39 (0.12)	0.90 (0.13)	0.49 (0.17)	0.96 (0.17)	0.34 (0.13)	0.78 (0.16)	0.41 (0.12)	0.76 (0.11)	0.35 (0.10)	
NAAG CRLB [%]	14.11 (4.10)	19.02 (5.07)	12.43 (3.87)	17.35 (6.07)	12.01 (3.14)	18.03 (5.15)	15.51 (5.08)	19.40 (6.62)	15.36 (5.01)	20.13 (6.40)	45.3 (11.6)
SNR	24.64 (4.41)	23.67 (3.84)	22.92 (4.50)	24.53 (4.02)	25.64 (4.82)	24.73 (4.17)	22.48 (6.13)	21.32 (5.56)	22.07 (4.23)	22.40 (3.37)	∅
FWHM [Hz]	11.43 (0.91)	12.09 (0.77)	11.28 (1.15)	11.13 (1.13)	10.21 (0.79)	11.02 (0.84)	11.64 (0.91)	12.33 (1.04)	12.26 (1.23)	12.38 (1.22)	∅

The two bottom rows present the SNR and FWHM values. The last columnshows the percentage of voxels inside the brain and FoV that meetthe criteria of good quality: CRLB<20%,FWHM<0.07ppm,SNR>5.The values are calculated as the average (standard deviation) acrossthe healthy volunteers imaged by 3D ECCENTRCIC at 3.4 mm isotropicwithAF=2(9 min:20 sec acquisition time).

Quantitative parameters for the quality of MRSI data are also listed inTable 1, including the precision ofmetabolite quantification by the Cramer-Rao lower bounds (CRLB), the SNR, andspectral linewidth (FWHM). It is worth noting that the values of spectral SNRand fitting CRLB are influenced by our reconstruction method. The CS-SENSE-LRreconstruction incorporates a low-rank model, effectively reducing noise,thereby enhancing SNR and lowering CRLB values. Consequently, the SNR and CRLBmetrics not only reflect the acquisition quality but also the effectiveness ofthe reconstruction process. Therefore, these values may vary from studies thatdo not employ similar reconstruction techniques. It can be seen that mean CRLBis below<20%for all the metabolites across the imaged whole-brain volume.In particular, mean CRLB is below<6%for the five metabolites with highest SNR (NAA, tCre, Cho,Glu, and Ins), between8%–10%for two metabolites (GABA and GSH) and between14%–20%for other two metabolites (Gln and NAAG). Across the brain,the mean SNR after the denoising reconstruction is larger than 20 and the meanlinewidth is less than 12 Hz (0.04 ppm).

### 4.4 Reproducibility of ECCENTRIC metabolic imaging

Results from repeated measurements are shown inFigure 5for Glu imaging. Due to high concentration of Glu in graymater, Glu images have high gray-white matter contrast and show fine structuraldetails of brain that can be used to visually assess the stability of theimaging with increasing AF. It can be seen that across all four scans in allfour subjects the metabolite images appear visually similar. We note that withrepeated measurements some anatomical differences may also be attributed toslight head motion. All metabolite maps, along with their respective CRLB, SNR,and FWHM maps for all four volunteers and AF, are presented inSupplementary FiguresS1–S7.

**Fig. 5.**
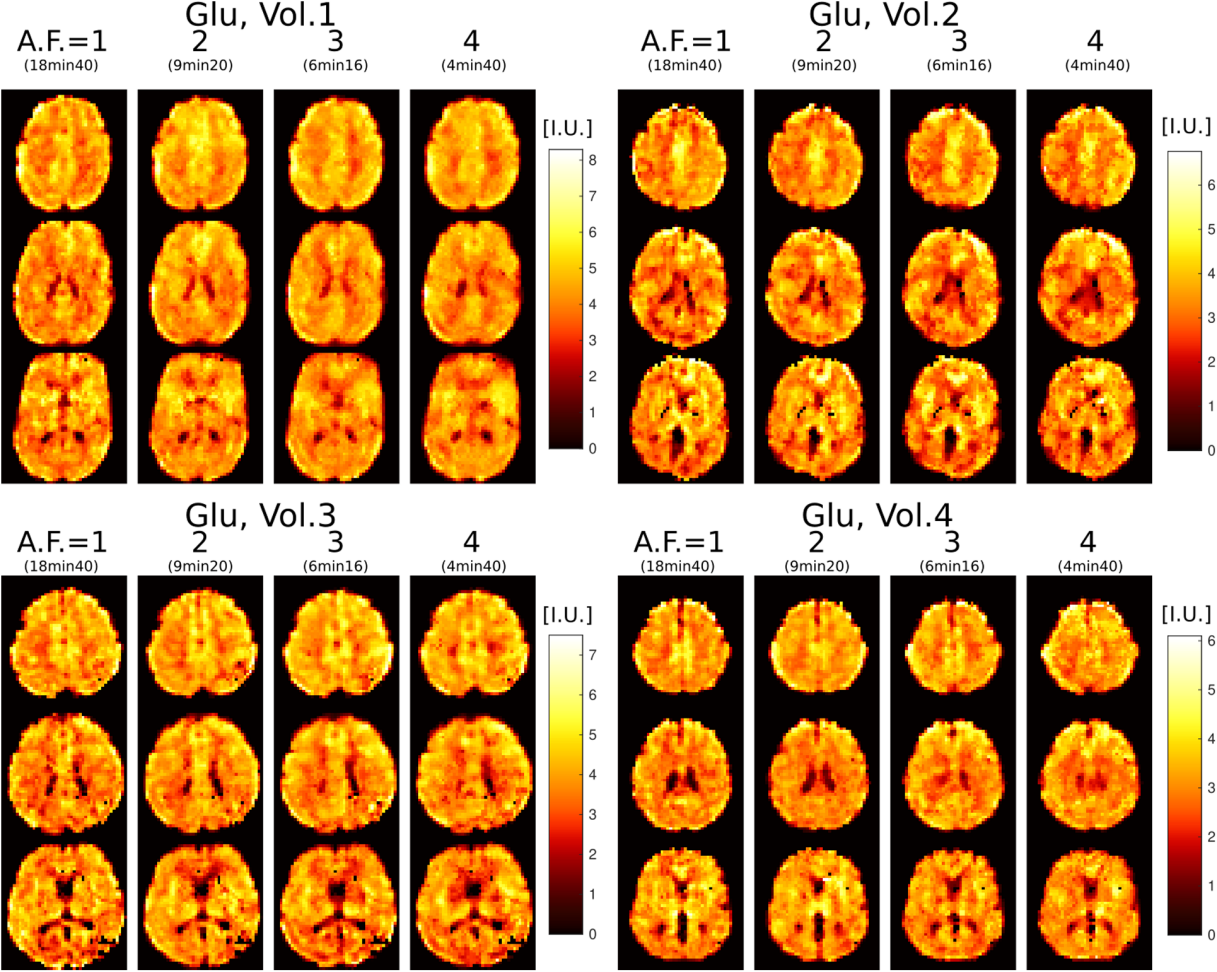
Glutamate imaging at3.4mm isotropic voxel size in four healthy volunteersscanned with 3D ECCENTRIC FID-MRSI in four successive acquisitions withincreasing accelerations AF = 1, 2, 3, and 4. Three slices areshown for each volunteer at each acceleration.

The inter-measurement COV for mapping metabolite concentrations across brainregions are presented inTable 2and theinter-subject COV inTable 3.Inter-measurement COV smaller than7%are observed for five metabolites (NAA, tCre, Ins, Cho, Glu)that are the most abundant in the brain. COV between8%−14%are obtained for Gln, GSH, and GABA. NAAG has higher COV inbrain regions (gray matter) where its concentration is low.

**Table 2.** The inter-measurement coefficient of variation (COV) for each metabolitedetermined in every lobe and tissue type for the 3D ECCENTRIC FID-MRSIacquired at 3.4 mm isotropic resolution in 6 min:16 sec (AF =3).

	Inter-measurement COV									
	Frontal		Limbic		Parietal		Occipital		Temporal	
	WM	GM	WM	GM	WM	GM	WM	GM	WM	GM
NAA	0.06	0.03	0.06	0.05	0.06	0.05	0.05	0.07	0.06	0.06
tCre	0.05	0.05	0.05	0.03	0.03	0.04	0.04	0.03	0.06	0.05
Ins	0.05	0.04	0.07	0.06	0.05	0.05	0.04	0.06	0.06	0.07
GPC+PCh	0.04	0.03	0.03	0.04	0.04	0.06	0.03	0.04	0.03	0.05
Glu	0.05	0.03	0.05	0.05	0.07	0.07	0.07	0.07	0.06	0.07
Gln	0.14	0.10	0.15	0.11	0.12	0.12	0.12	0.07	0.14	0.08
GABA	0.07	0.11	0.05	0.07	0.09	0.12	0.05	0.11	0.06	0.11
GSH	0.12	0.10	0.14	0.13	0.13	0.13	0.13	0.10	0.14	0.11
NAAG	0.23	0.41	0.28	0.33	0.24	0.55	0.26	0.40	0.28	0.54

**Table 3.** The inter-subject coefficient of variation (COV) for each metabolitedetermined in every lobe and tissue type for the 3D ECCENTRIC FID-MRSIacquired at 3.4 mm isotropic resolution in 6 min:16 sec (AF =3).

	Inter-subject COV									
	Frontal		Limbic		Parietal		Occipital		Temporal	
	WM	GM	WM	GM	WM	GM	WM	GM	WM	GM
NAA	0.21	0.15	0.23	0.17	0.23	0.17	0.24	0.23	0.23	0.22
tCre	0.10	0.05	0.12	0.07	0.11	0.05	0.09	0.09	0.11	0.09
Ins	0.18	0.15	0.22	0.17	0.22	0.14	0.21	0.26	0.24	0.19
GPC+PCh	0.06	0.07	0.07	0.06	0.10	0.10	0.12	0.14	0.11	0.15
Glu	0.19	0.13	0.20	0.16	0.20	0.15	0.18	0.18	0.18	0.20
Gln	0.16	0.25	0.12	0.16	0.18	0.21	0.21	0.23	0.17	0.18
GABA	0.37	0.33	0.39	0.38	0.36	0.36	0.38	0.39	0.37	0.40
GSH	0.13	0.09	0.15	0.11	0.13	0.07	0.11	0.13	0.11	0.12
NAAG	0.32	0.37	0.35	0.29	0.45	0.47	0.50	0.48	0.38	0.29

The results fromTables 2and3show that 3D ECCENTRIC FID-MRSI hadreproducible and stable performance in three repeat measurements. Theinter-measurement COV exhibits a markedly lower value (2–4 times smaller)compared to the inter-subject COV. The former is primarily influenced bytechnical variability, whereas the latter reflects a combination of technicaland biological variability. The quantification of the five main metabolites thathave the highest SNR in brain MRSI (NAA, tCre, Cho, Ins, Glu) shows the lowestvariability, with a slight increase in the case of less abundant metabolites(Gln, GABA, and GSH). The highest variability is noticed for NAAG outside of thefronto-parietal white matter due to its specific localization in this brainarea. We note that in-vivo variability of metabolite quantification in repeatmeasurements is also influenced by patient motion and scanner stability inaddition to 3D ECCENTRIC FID-MRSI, hence methods that reduce the effects ofmotion and field drift (Andronesi et al.,2021;Bogner et al., 2014) arelikely to reduce variability.

### 4.5 Ultra-high resolution metabolic imaging of human brain using 3D ECCENTRIC FID-MRSI

**Fig. 6.**
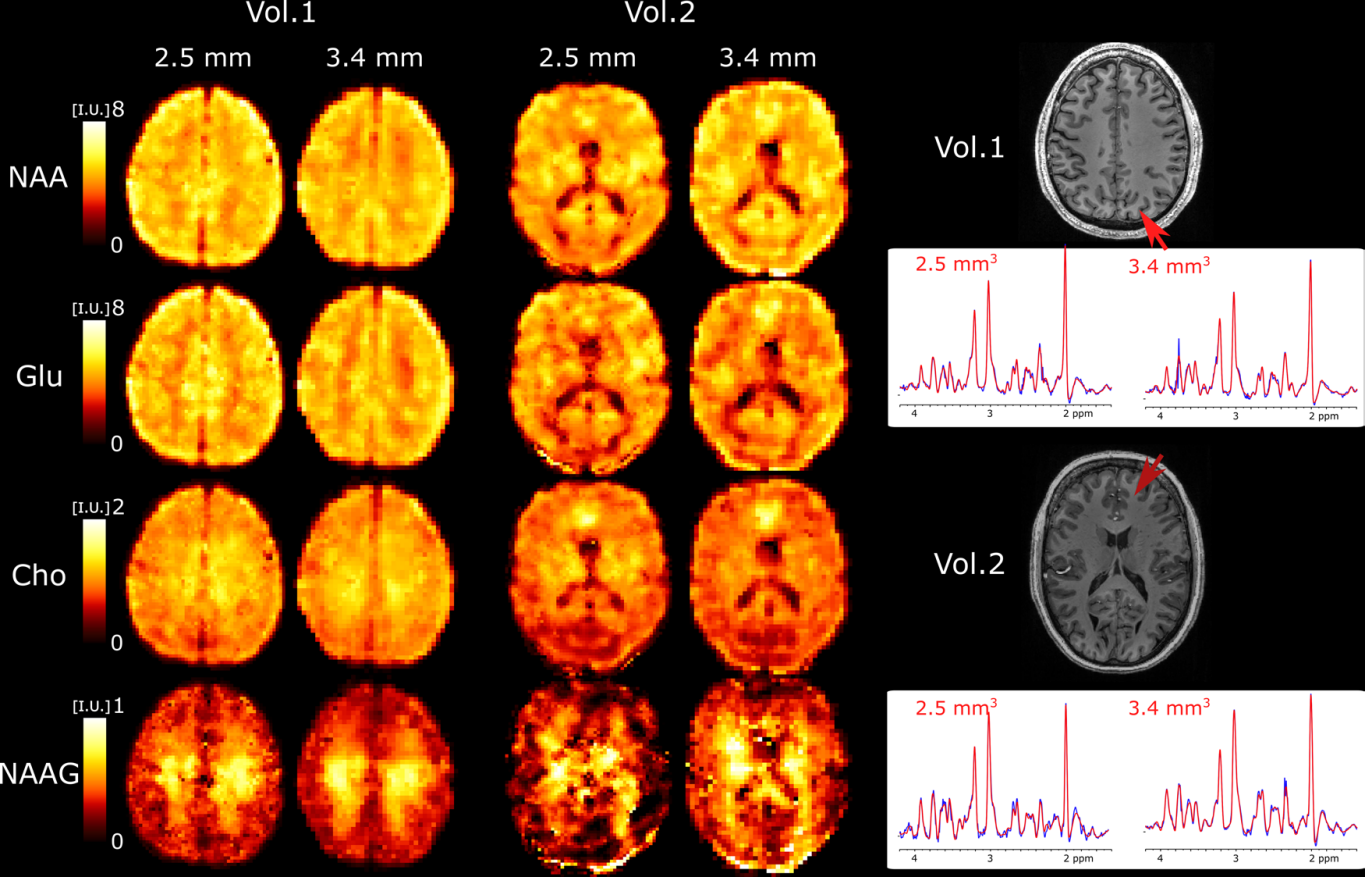
Ultra-high resolution metabolic imaging acquired with 3D ECCENTRICFID-MRSI at2.5mm isotropic voxel size (AF=4, TA =10min:26s) in two healthy volunteers. The ultra-high resolutionmetabolic imaging is compared to 3D ECCENTRIC FID-MRSI at the typicalvoxel size of3.4mm isotropic (AF=2, TA =9min:20s). Right, two spectra from both spatial resolution andcorresponding to the red arrow location are shown. The blue linerepresents the MRSI data, and the red line is the fit performed byLCModel.

We further explored the performance of 3D ECCENTRIC FID-MRSI for ultra-highresolution metabolic imaging in several healthy volunteers. Based on the highSNR of the 3.4 mm data after the denoising reconstruction, we expected thatsmaller voxels at higher resolution will still provide sufficient SNR formetabolite imaging.Figure 6showsmetabolic images obtained using 3D ECCENTRIC FID-MRSI with isotropic voxel sizeof 2.5 mm in two healthy volunteers.

To achieve a feasible scan time, we used CS withAF=4. We demonstrated at the beginning of our work thatAF=4provides metabolic maps that are similar to those obtainedthrough fully sampled 3D ECCENTRIC FID-MRSI. TheAF=4acceleration enabled the acquisition of 3D ECCENTRIC FID-MRSIat2.5mm isotropic resolution in 10 min:26 sec. For comparison, wealso acquired the typical 3D ECCENTRIC FID-MRSI at3.4mm isotropic withAF=2acceleration in 9 min:20 sec. As readily apparent by visualinspection, the metabolic maps at higher spatial resolution provide sharperdelineation of the brain structure. No compromise is visible forsignal-to-noise, contrast-to noise or other data quality metric at ultra-highresolution compared to typical resolution. We note that the acquisition time of3D ECCENTRIC FID-MRSI at2.5mm withAF=4is only slightly longer(1min) than at3.4mm withAF=2. However, for the same acceleration factor the acquisitiontime of 3D ECCENTRIC FID-MRSI at3.4mm is2.2times faster than at2.5mm.

## 5 Discussion

Our results here demonstrate that 3D ECCENTRIC FID-MRSI at 7T can simultaneouslyimage an extended neurochemical panel of 10–14 metabolites with high SNR athigh spatial resolution across whole-brain and with acquisition times that arefeasible for human imaging. Particularly, we showed that the acquisition of fastnon-Cartesian MRSI can be further accelerated up to 4-fold by CS, allowing metabolicimaging at 3.4 mm isotropic resolution in 4 min:40 sec and at 2.5 mm isotropicresolution in 10 min:26 sec, respectively. The CS-SENSE-LR reconstruction producesmetabolic images with an effective voxel size identical to the nominal size (Klauser et al., 2021). This provides anadvantage compared to other filtered reconstructions (Hangel et al., 2020;Hingerl et al., 2020) which increase the effective voxel volume.ECCENTRIC preserves the features of metabolic images across accelerations. Whenaccelerated up to 4-fold by CS the loss of image quality is minor and the metabolicimages effectively visualize the laminar structure of the brain similar to theunaccelerated (AF = 1) ground truth. Through the design of ECCENTRICacquisition and denoising reconstruction, SNR is enhanced even at highaccelerations. This enhancement is achieved by fully sampling the center of k-spaceand employing a low-rank reconstruction technique.

Here, we investigated the performance of 3D ECCENTRIC FID-MRSI for two applicationsscenarios: 1) high resolution metabolic imaging (3.4 mm in 4 min:40 sec) for studiesthat need to minimize imaging time, and 2) ultra-high resolution metabolic imaging(2.5 mm in 10 min:26 sec) for applications that need to probe brain neurochemistrywith highest structural detail. Both of these protocols represent a significantadvancement for non-invasive imaging of human brain metabolism by*invivo*MRSI. Their performance level is comparable to other advanced MRimaging methods, such as CEST and perfusion imaging.

Results obtained with the 3.4 mm imaging protocol show good delineation of brainstructures. At 2.5 mm ultra-high resolution there is increased gray-white mattercontrast of metabolites due to less partial volume effect which reveals the brainfolding more clearly than at 3.4 mm. Several metabolites show particularly highcontrast between gray and white matter in healthy brain, such as the energy buffertCre, the neurotransmitter Glu, and the dipeptide NAAG. In particular, NAAG is themost abundant dipeptide in the brain, which is selectively localized in severalregions (Pouwels & Frahm, 1998) whereit neuromodulates the glutamatergic synapses required for normal brain activity.Importantly, NAAG is also implicated in neurodegenerative diseases, schizophrenia,stroke, epilepsy, traumatic brain injury and pain (Morland & Nordengen, 2022). Our data show the highest resolutionof 3D imaging for NAAG to date. 3D ECCENTRIC FID-MRSI provides images of NAAG braindistribution, which could offer valuable insights into both basic and clinicalneuroscience questions. Good-quality metabolic images are obtained also for some ofthe most important but challenging metabolites such as GABA, Gln, and GSH. Thecombination of higher SNR and narrower linewidth (FWHM) results in lower CRLB forGABA, Gln, and GSH. The potential of short-echo FID spectra to detect GABA, Gln, andGSH at ultra-high field is supported also by previous findings reported in 9.4Tstudies (Nassirpour, Chang, & Henning,2018a;Ziegs et al., 2023). Here,we extend the imaging of NAAG, GABA, Gln and GSH from single-slice to whole-brainand show that this is feasible at 7T which is more available for ultra-high fieldhuman imaging compared to 9.4T. As the 7T scanners have gained approval byregulatory agencies worldwide, the 7T imaging has reached clinical use and we expectECCENTRIC will have a great contribution in clinical studies.

While correlation coefficients between the ground truth and accelerated ECCENTRICacquisitions generally exhibit lower values for metabolic imaging (Fig. 4) compared to water imaging (Fig. 3;Supplementary Fig. S13), visual assessments indicate that thequality of metabolite mapping in the healthy brain is consistently preserved acrossall acceleration levels. Notably, the discrepancy between the results formetabolites and water images arises from the fact that, unlike water images wherecorrelation coefficients are directly determined from reconstructed images, thecorrelation coefficients for metabolic images are influenced by additionalprocessing steps such as water removal, fat removal, and LCModel fitting. Theseadditional processing steps of MRSI data introduce variability that contributes tothe lower correlation coefficients observed in metabolic imaging results.

The 3D ECCENTRIC FID-MRSI showed robust performance in healthy volunteers. The lowvariability in repeat measurements indicates high precision of metabolitequantification and significant potential for longitudinal studies to detectmetabolite changes due to disease, treatment and functional tests. The high qualityof the data was achieved through the use of third-order shimming, which providesmore uniform$B_{0}$field across the brain, as well as the shortened scan time, which minimized thescanner drift and possibility of subject motion. The scanner drift typically rangedfrom 5–10 Hz over a 10 min scan time.

ECCENTRIC encoding is highly versatile with flexible choice of FoV, spatialresolution, and spectral bandwidth that can be set to optimize SNR and acquisitiontime. The advantage and strength of ECCENTRIC is enabled by the possibility tofreely choose the radius and position of circle trajectories in covering thek-space: 1) the free choice of circle radius allows freedom in setting FoV, spatialresolution, and spectral bandwidth without the need of temporal interleaving, 2) thefree choice of circle center position allows freedom for random undersampling thek-space to accelerate acquisition by CS. This flexibility is particularly importantfor^1^H-MRSI at 7T and beyond, due to the increased spectral bandwidthrequired which limits the duration ofk-spacetrajectories. In addition, free choice of circle position should enable FoV withdifferent extent along the axial dimensions for additional time saving, which cannotbe achieved by concentric, rosette and spiral trajectories.

ECCENTRIC flexibility in setting FoV, resolution, and spectral bandwidth by varyingthe circle radius and CS undersampling to optimize SNR and acquisition time is showninSupplementary FiguresS14–S16. These results indicate that, when using the same imageresolution and acquisition time, ECCENTRIC provides a higher SNR for protocols thatuse smaller circle radii and higher acceleration compared to protocols using largercircle radii and lower acceleration. This flexibility allows to adapt ECCENTRICacquisition to resolutions required across a range of FoV. Although we demonstratedECCENTRIC for human brain imaging, this method can be used for ultra-high resolutionmetabolic imaging of mice brain where submillimeter resolution is required for acentimeter FoV. ECCENTRIC sampling was specifically designed for MRSI SSEmeasurements, requiring a trajectory that is successively repeated at a highfrequency. While this high-frequency repeated sampling is not a prerequisite fortraditional MRI technique, certain imaging techniques may demand the rapidacquisition of successive echoes and could potentially benefit from ECCENTRICsampling at UHF.

There are some limitations in the current implementation. In particular,reconstruction time of 3D ECCENTRIC data requires several hours. For example, usinga64×64×31matrix size for3.4mm isotropic, the water removal step took 1 h, the CS-SENSE-LRreconstruction took 3 h on a GPU (or 12 h on a CPU), and the parallel LCModelfitting took 1 h on a high-performance server such as the Dell PowerEdge R7525 (with64 cores of 2.9GHz and 128M cache, 512GB RAM, and 3 NVIDIA Ampere A40 GPUs). Thiscomputation time may be considered relatively long for routine clinicalapplications. Also, at the moment subject motion or scanner drift is not correctedduring ECCENTRIC acquisition, which may increase the variability of metabolitequantification. The metabolite concentrations were not provided in absolute unitssuch as millimolar but expressed in institutional units (I.U.) relative to the waterreference, which still provides comparable values across subjects and scanners. Wenote that for absolute quantification of FID-MRSI data only the T1 relaxationcorrection is needed, while ultra-short(<1ms) echo-time makes T2 relaxation negligible. Our current ECCENTRIC design restrictscircle positioning to a stack-of-ECCENTRIC configuration without tilting or rotatingthe circles. While this simplifies reconstruction, it also limits exploration ofmore complex sampling schemes, such as fully tilted circles, which could enhancek-space sampling efficiency. However, implementing tilted circles in 3D k-spacewould pose practical challenges due to the significantly increased computationaldemands of a full 3D discrete Fourier transform. Nevertheless, alternativereconstruction methods, such as NUFFT-based gridding (Fessler, 2007), may offer solutions to overcome theselimitations and enable more thorough exploration of sampling strategies in 3Dk-space.

Artificial intelligence is reshaping the landscape of MR image reconstruction,demonstrating improved performance and enabling more efficient and sparser dataacquisition (Lin et al., 2021). In the domainof MRSI, the application of deep learning for reconstruction is an evolving field,marked by notable progress in super-resolution reconstruction (Dong et al., 2022;Li etal., 2020), learned-subspace approaches (Lam et al., 2020), channel combination strategies (Motyka et al., 2021), and parallel MRSI methods (Nassirpour, Chang, & Henning, 2018b).These encouraging findings suggest that the integration of deep learning holdssubstantial potential to enhance reconstruction outcomes over the model-basedCS-SENSE-LR reconstruction employed in this study. Such improvement may manifest inimproved quality of metabolite maps or even enable higher AF, consequently leadingto faster acquisition.

In summary, we have introduced ECCENTRIC an advanced acquisition-reconstructionmethod for MRSI that pushes the boundaries of spatial and temporal capabilities for*in vivo*metabolic imaging. Although here we specificallydemonstrated ECCENTRIC for MRSI at 7T ultra-high field, this method is not limitedto this field and could be used at higher(≥9.4T)and lower (3T) fields. When combined with FID-MRSI acquisition, ECCENTRIC hasdemonstrated outstanding performance in the comprehensive mapping of metabolitesthroughout the entire brain, encompassing crucial neurotransmitters such as Glu andGABA. Anticipating that ECCENTRIC will pave the way for novel advancements inneuroscience, we envision its potential to provide detailed insights into brainneurochemistry in both healthy and pathological conditions. This innovation ispoised to address crucial questions and facilitate groundbreaking discoveries inboth fundamental research and clinical studies.

## Supplementary Material

Supplementary Material

## Data Availability

Data can be obtained from the corresponding author (O.C.A.) upon request withinstitutional agreement for data sharing. 3D ECCENTRIC FID-MRSI sequence andreconstruction can be obtained from the corresponding author (A.K.) upon request forSiemens Healthcare MAGNETOM Terra / Terra.X MRI system.
