# Supplementary material for: ECCENTRIC: A fast and unrestrained approach for high-resolution in vivo metabolic imaging at ultra-high field MR
Source: Imaging Neurosci (Camb). 2024 Oct 15;2:imag-2-00313. doi: 10.1162/imag_a_00313 (PMC11638761; doi:10.1162/imag_a_00313)
Supplement: Supplementary Material [file imag_a_00313-supp.pdf]

Supplementary material to *ECCENTRIC: a fast and unrestrained approach for high-resolution in vivo metabolic imaging at ultra-high field MR*

I. ALGORITHM FOR CS-SENSE-LR RECONSTRUCTION

The following pseudocode describes the algorithm used to reconstruct the 3D-ECCENTRIC MRSI data with the CS-SENSE-LR model presented in eq.(2) in the main manuscript. This algorithm is built on the TGV reconstruction proposed by Knoll *et al.* [1] and extended to include MRSI data with a low-rank assumption and skull lipid suppression.

function CS-SENSE-LR-Recon($\mathbf{s}, \lambda, \beta$)
  $\mathbf{L} \leftarrow \mathcal{M}_{skull}\mathcal{A}^*\mathbf{s}$
  $\mathcal{P}_\mathbf{L} \leftarrow (\mathbb{1} + \beta\mathbf{L}^*\mathbf{L})^{-1}$
  $[\mathbf{U}, \Sigma, \mathbf{V}] \leftarrow \text{SVD}(\mathcal{P}_\mathbf{L}\mathcal{A}^*\mathbf{s})$
  $\mathbf{V} \leftarrow \Sigma \cdot \mathbf{V}$
  $\bar{\mathbf{V}} \leftarrow \mathbf{V}$, $\bar{\mathbf{U}} \leftarrow \mathbf{U}$, $\bar{\mathbf{L}} \leftarrow \mathbf{L}$
  $\mathbf{w}, \bar{\mathbf{w}} \leftarrow 0$, $\mathbf{p}, \mathbf{q}, \mathbf{r} \leftarrow 0$
  $\tau, \sigma > 0$
  repeat
    $\mathbf{p} \leftarrow \text{proj}_\lambda(\mathbf{p} + \sigma\nabla(\bar{\mathbf{U}} - \bar{\mathbf{w}}))$
    $\mathbf{q} \leftarrow \text{proj}_{2\lambda}(\mathbf{q} + \sigma\mathcal{E}\bar{\mathbf{w}})$
    $\mathbf{r} \leftarrow \text{prox}_2^\sigma\left(\mathbf{q} + \sigma\left(\mathcal{A}(\bar{\mathbf{U}}\bar{\mathbf{V}} + \bar{\mathbf{L}}) - \mathbf{s}\right)\right)$

    $\mathbf{U}_{old} \leftarrow \mathbf{U}$
    $\mathbf{V}_{old} \leftarrow \mathbf{V}$
    $\mathbf{L}_{old} \leftarrow \mathbf{L}$
    $\mathbf{w}_{old} \leftarrow \mathbf{w}$

    $\mathbf{U} \leftarrow \mathbf{U} + \tau\left(\text{div}_1\mathbf{p} - (\mathcal{P}_\mathbf{L}\mathcal{A}^*\mathbf{r}) \cdot (\bar{\mathbf{V}}^*/\Sigma^2)\right)$
    $\mathbf{V} \leftarrow \mathbf{V} - \tau\left(\bar{\mathbf{U}}^* \cdot (\mathcal{P}_\mathbf{L}\mathcal{A}^*\mathbf{r})\right)$
    $\mathbf{L} \leftarrow \mathbf{L} - \tau\left(M_{skull}\mathcal{A}^*\mathbf{r}\right)$
    $\mathbf{w} \leftarrow \mathbf{w} + \tau\left(\mathbf{p} + \text{div}_2\mathbf{q}\right)$

    $\bar{\mathbf{U}} \leftarrow 2\mathbf{U} - \mathbf{U}_{old}$
    $\bar{\mathbf{V}} \leftarrow 2\mathbf{V} - \mathbf{V}_{old}$
    $\bar{\mathbf{L}} \leftarrow 2\mathbf{L} - \mathbf{L}_{old}$
    $\bar{\mathbf{w}} \leftarrow 2\mathbf{w} - \mathbf{w}_{old}$
    $\mathcal{P}_\mathbf{L} \leftarrow (\mathbb{1} + \beta\bar{\mathbf{L}}^*\bar{\mathbf{L}})^{-1}$

    for $k = 1, \ldots, K$ **do**
      $\Sigma_{k,k} \leftarrow \sqrt{\sum_t |V_{k,t}|^2}$
      $nu \leftarrow \sqrt{\sum_r |U_{r,k}|^2}$
      for $r = 1, \ldots, N_r$ **do**
        $U_{r,k} \leftarrow U_{r,k}/nu$
      end for
    end for

  until $\mathbf{U}$ and $\mathbf{V}$ are converged

  return $[\mathbf{U}, \mathbf{V}]$

end function

In the pseudocode provided above, $\mathbf{s}$ is the measured data, $\mathcal{A}$ represents the forward operator, $\mathcal{FCB}$, fully described in the main manuscript, while $\mathcal{A}^*$ denotes its adjoint. $\mathbf{U}$ and $\mathbf{V}$ stand for the spatial and temporal components of the low-rank assumption introduced in main manuscript eq.(1).

It is assumed that the $l2$-norm of $\mathbf{U}$ components equals 1 ($\sqrt{\sum_r |U_{r,k}|^2} = 1$, $\forall k$), while Σ is a K by K diagonal matrix containing the $l2$-norm of the temporal components, $\mathbf{V}$,($\Sigma_{k,k} = \sqrt{\sum_t |V_{k,t}|^2}$, $\forall k$).

$\mathbf{L}$ denotes the skull lipid signal (an N_r by T array) which is concurrently estimated alongside $\mathbf{U}$ and $\mathbf{V}$ within the algorithm. $\mathcal{M}skull$ represents a mask operator nullifying all MRSI data outside the spatial support of the skull.

The matrix multiplication is denoted by the symbol "$\cdot$", expressing the identities $(\bar{\mathbf{U}}\bar{\mathbf{V}}) \cdot (\bar{\mathbf{V}}^*/\Sigma^2) = \bar{\mathbf{U}}$ and $\bar{\mathbf{U}}^* \cdot (\bar{\mathbf{U}}\bar{\mathbf{V}}) = \bar{\mathbf{V}}$.

$\mathcal{P}_{\mathbf{L}}$ represents the spectral projection matrix onto the metabolite spectral subspace, commonly referred to as L2-lipid-regularization lipid suppression [2]. It is calculated using the expression $(\mathbb{1}+\beta\mathbf{L}^*\mathbf{L})^{-1}$, with the notation adopted from [2]. The value of β is established at the maximum value where the mean absolute value of the diagonal of $\mathcal{P}_{\mathbf{L}}$ is greater than 0.8. This empirically determined threshold ensures full lipid signal suppression while minimizing distortion of the metabolite signal.

proj_α denotes the Euclidian projector that is given by $\text{proj}_\alpha(\mathbf{x}) = \frac{\mathbf{x}}{\max(1,|\mathbf{x}|/\alpha)}$, and prox_σ stands for the proximal mapping of the data fitting dual variable, $\mathbf{r}$, with $\text{prox}_\sigma(\mathbf{x}) = \frac{\mathbf{x}}{1+\sigma}$ [1].

The regularization parameter λ was adjusted to 3×10^{-4} by gradually increasing it from a low value until the noise-like artifacts in the metabolite maps disappeared as described by by Knoll *et al.* [1].

II. ADDITIONAL DATA

A. 3D ECCENTRIC ^{1}H-FID-MRSI results in four healthy volunteers

Here, we present the comprehensive dataset comprising reconstructed metabolite maps along with their corresponding error estimates (CRLB). The dataset includes NAA, tCre, Cho, Ins, NAAG, Glu, Gln, GSH and GABA. These data were acquired from four volunteers through successive acquisitions, with acceleration factors (AF) ranging from 1 to 4.

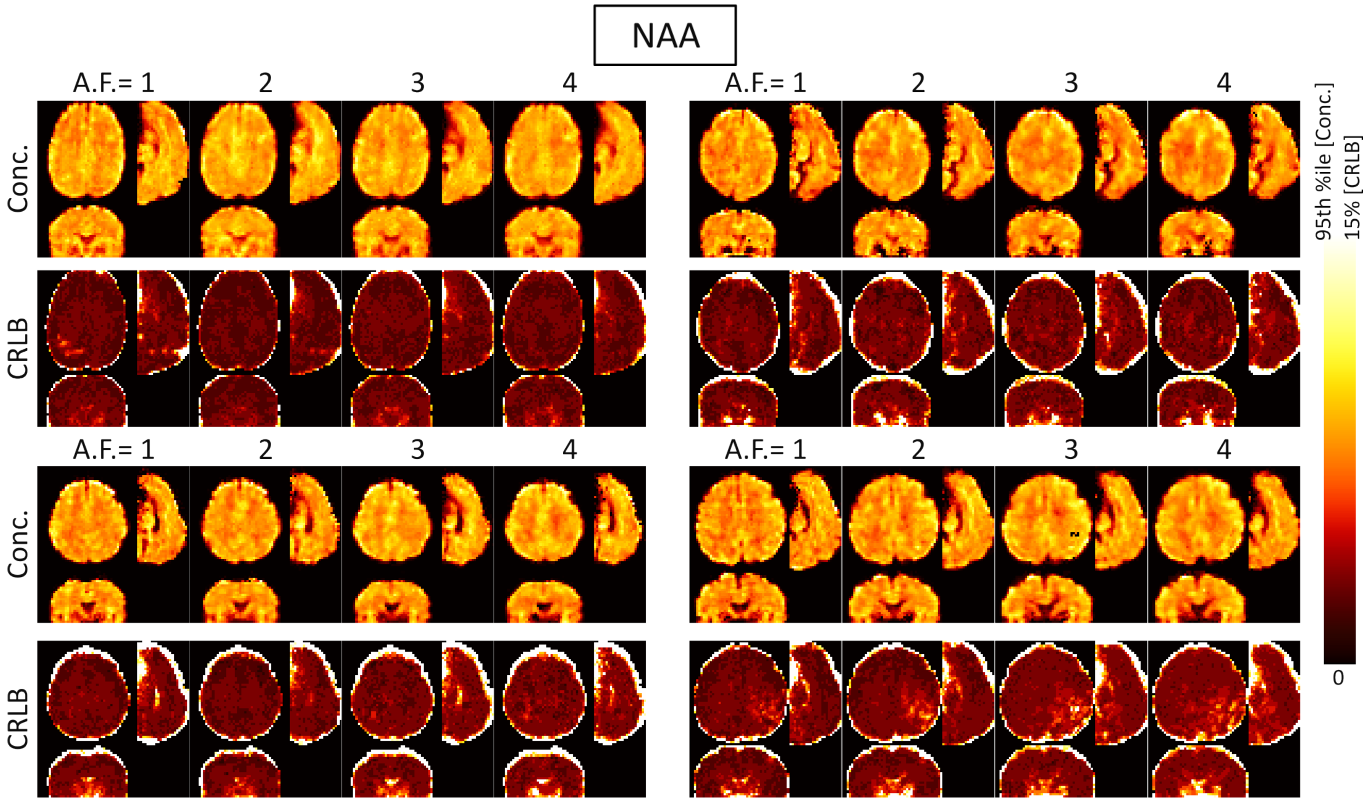

FIG. S1: N-acetylaspartate (NAA) concentration maps obtained from four consecutive measurements with increasing acceleration factors (A.F.) using 3D ECCENTRIC ^{1}H-FID-MRSI on four healthy volunteers. The color scale ranges from 0 to the 95th percentile value derived from all voxels in the brain. Corresponding Cramér-Rao Lower Bound (CRLB) maps are displayed with a color scale extending up to 15%

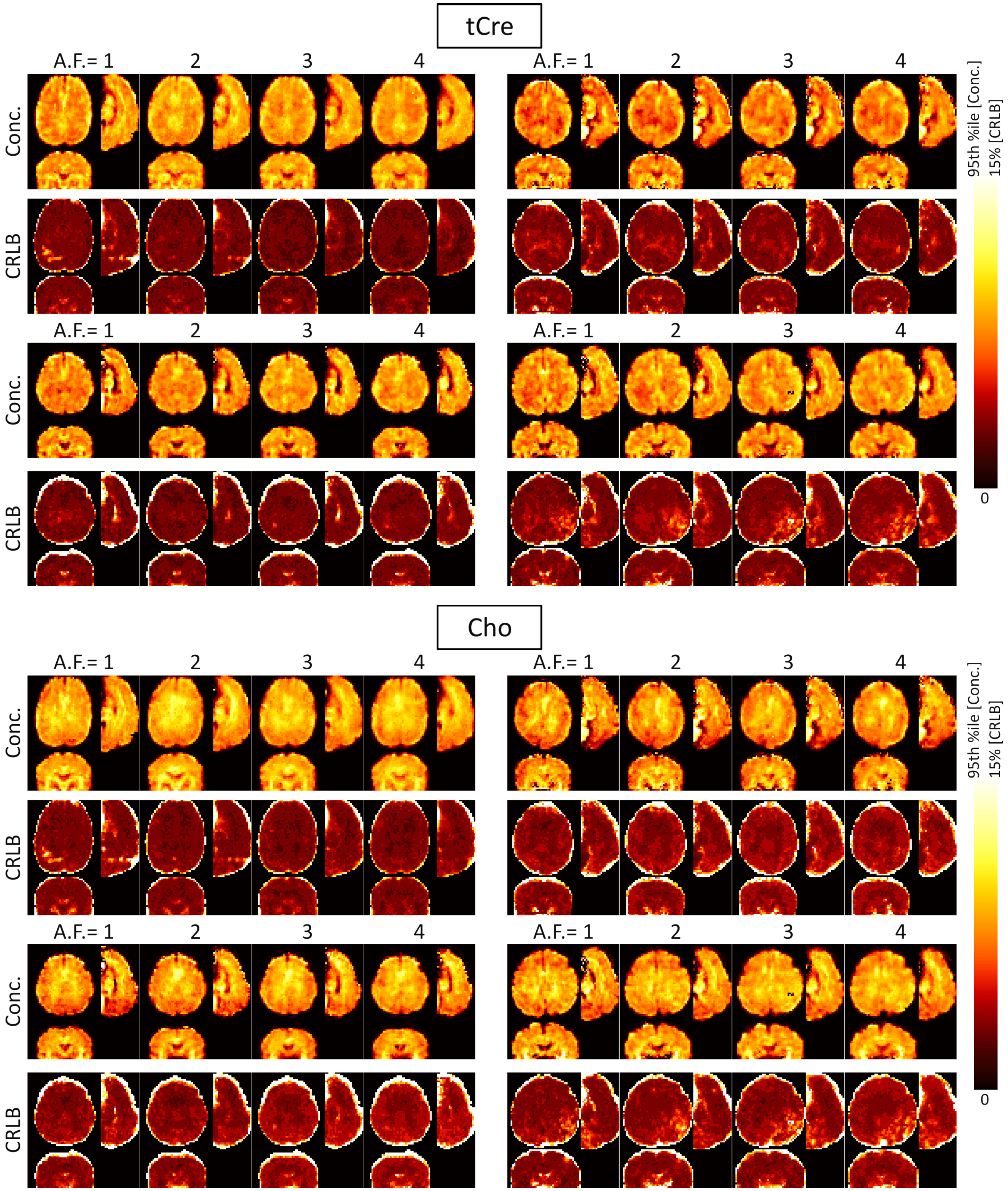

FIG. S2: Creatine + phosphocreatine (tCr) and Phosphocholine + glycerophosphocholine (Cho) concentration maps obtained from four consecutive measurements with increasing acceleration factors (A.F.) using 3D ECCENTRIC ^{1}H-FID-MRSI on four healthy volunteers. The color scale ranges from 0 to the 95th percentile value derived from all voxels in the brain. Corresponding Cramér-Rao Lower Bound (CRLB) maps are displayed with a color scale extending up to 15%

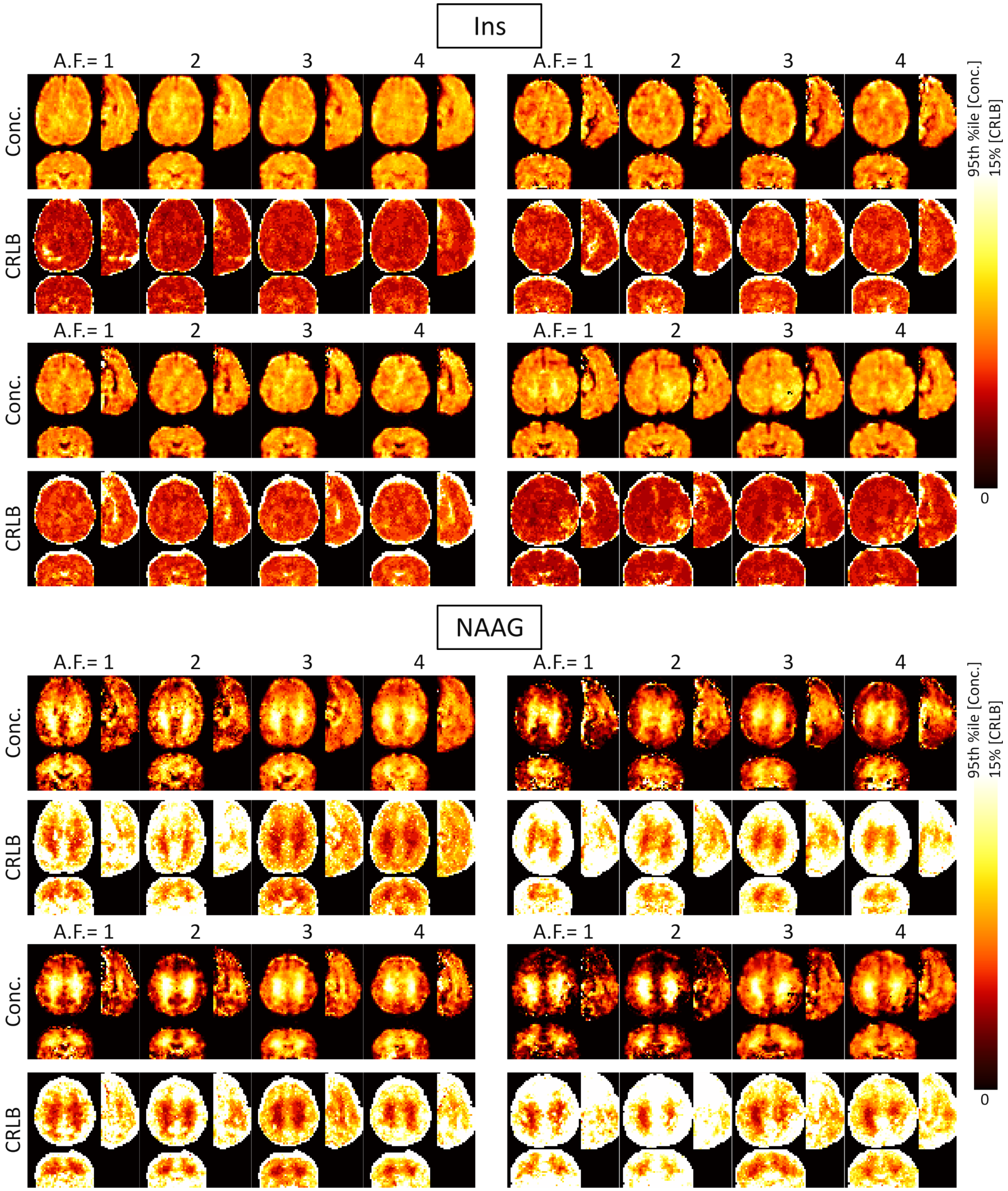

FIG. S3: Myo-inositol (Ins) and N-acetyl aspartylglutamate (NAAG) concentration maps obtained from four consecutive measurements with increasing acceleration factors (A.F.) using 3D ECCENTRIC ^{1}H-FID-MRSI on four healthy volunteers. The color scale ranges from 0 to the 95th percentile value derived from all voxels in the brain. Corresponding Cramér-Rao Lower Bound (CRLB) maps are displayed with a color scale extending up to 15%

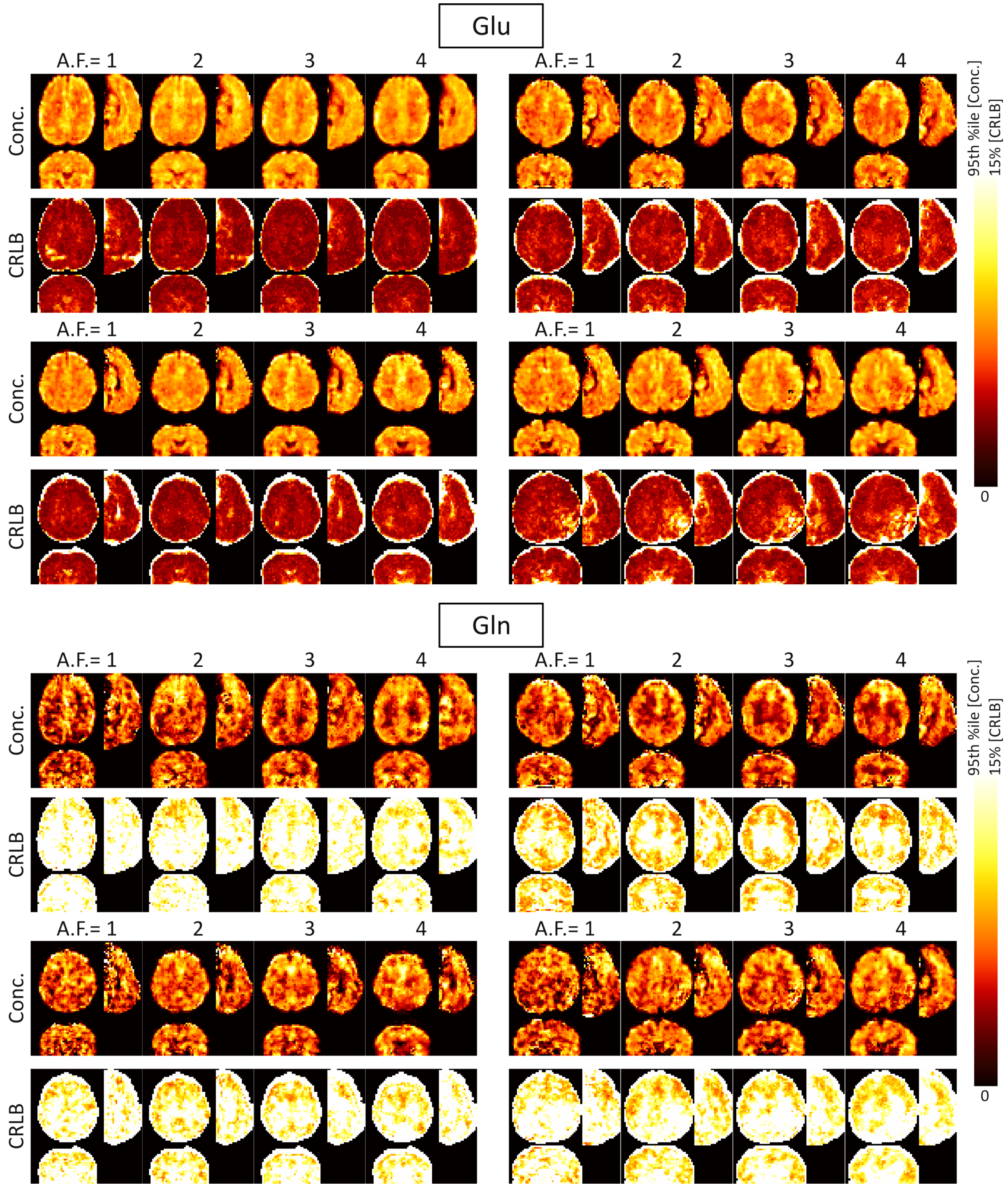

FIG. S4: Glutamate (Glu) and Glutamine (Gln) concentration maps obtained from four consecutive measurements with increasing acceleration factors (A.F.) using 3D ECCENTRIC ^{1}H-FID-MRSI on four healthy volunteers. The color scale ranges from 0 to the 95th percentile value derived from all voxels in the brain. Corresponding Cramér-Rao Lower Bound (CRLB) maps are displayed with a color scale extending up to 15%

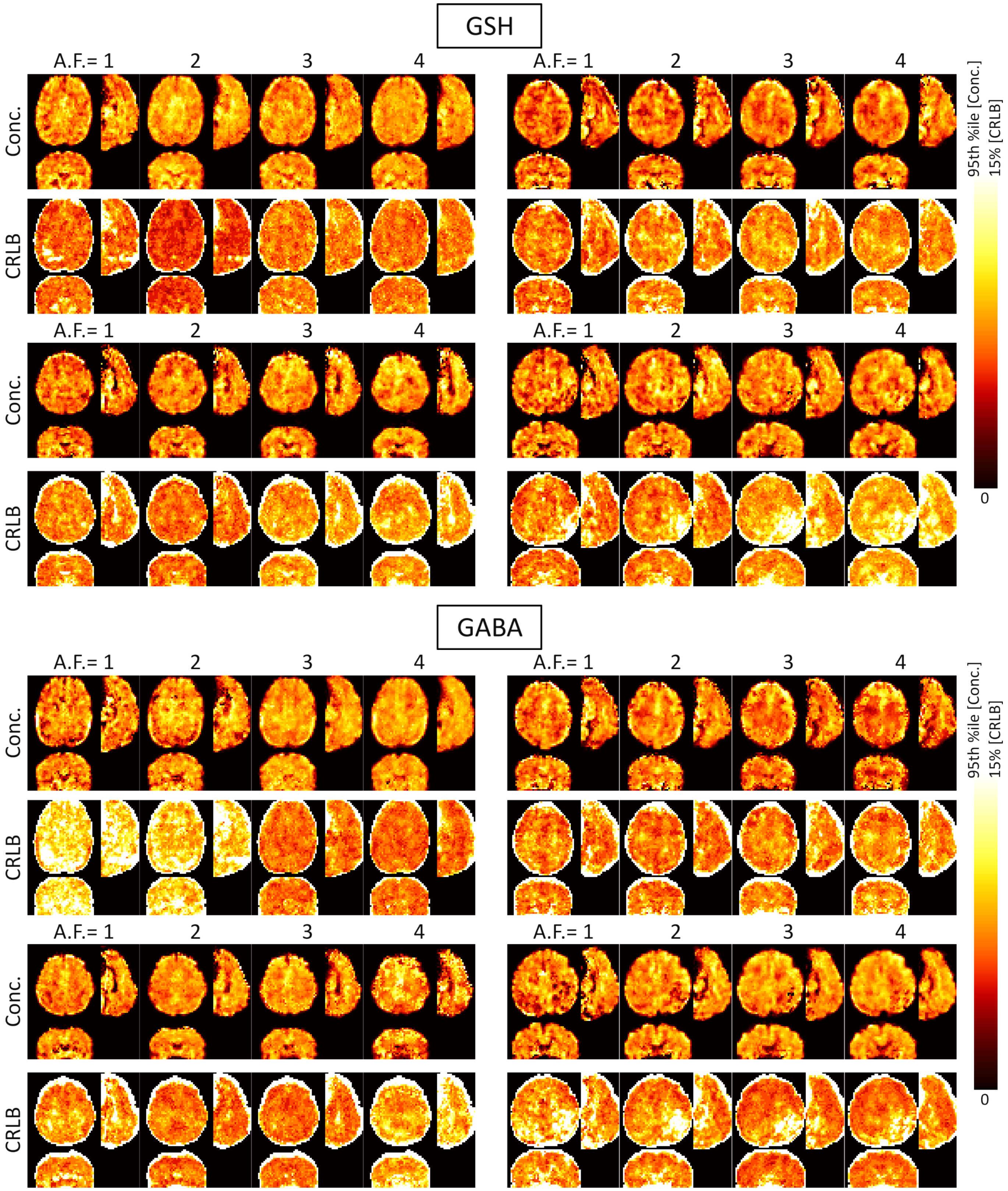

FIG. S5: Glutathione (GSH) and Gamma-aminobutyric acid (GABA) concentration maps obtained from four consecutive measurements with increasing acceleration factors (A.F.) using 3D ECCENTRIC ^{1}H-FID-MRSI on four healthy volunteers. The color scale ranges from 0 to the 95th percentile value derived from all voxels in the brain. Corresponding Cramér-Rao Lower Bound (CRLB) maps are displayed with a color scale extending up to 15%

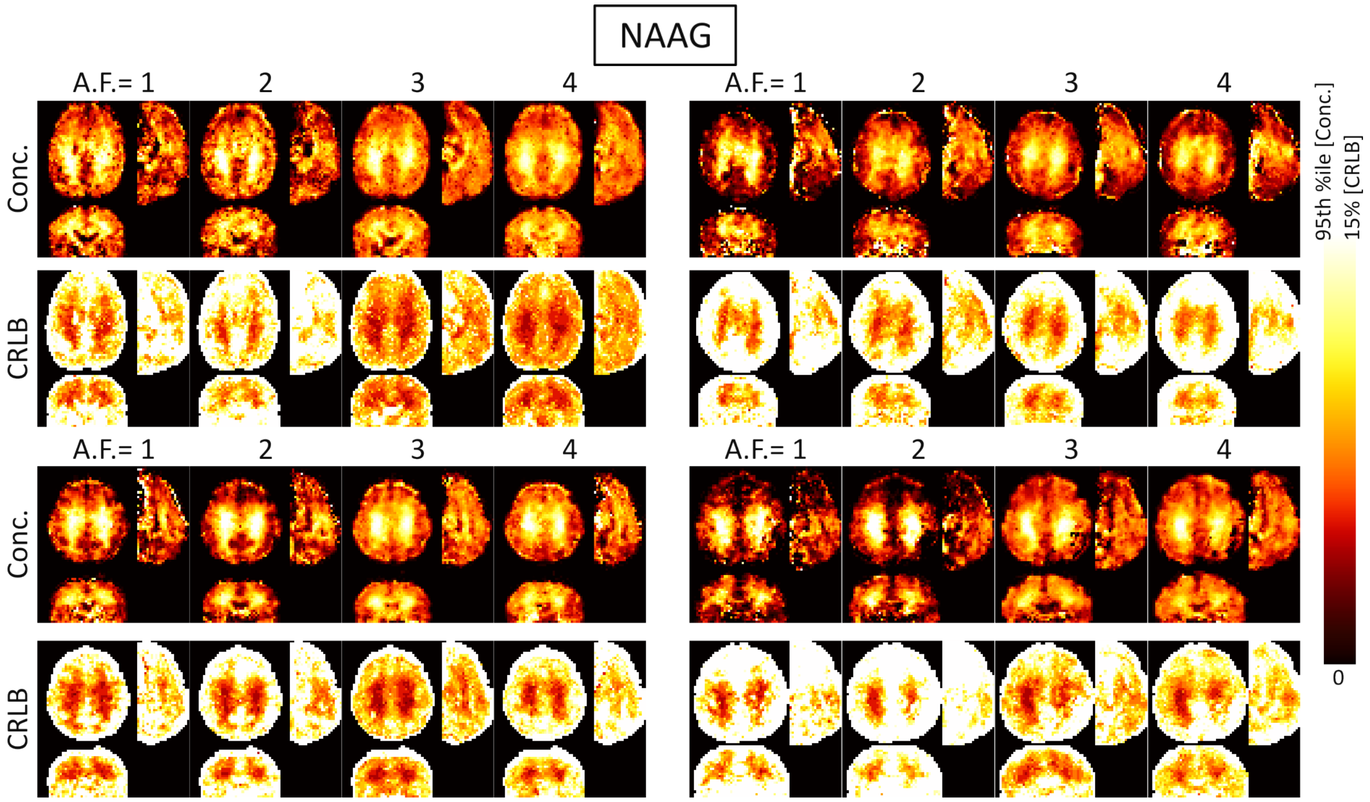

FIG. S6: N-acetyl aspartylglutamate (NAAG) concentration maps obtained from four consecutive measurements with increasing acceleration factors (A.F.) using 3D ECCENTRIC ^{1}H-FID-MRSI on four healthy volunteers. The color scale ranges from 0 to the 95th percentile value derived from all voxels in the brain. Corresponding Cramér-Rao Lower Bound (CRLB) maps are displayed with a color scale extending up to 15%

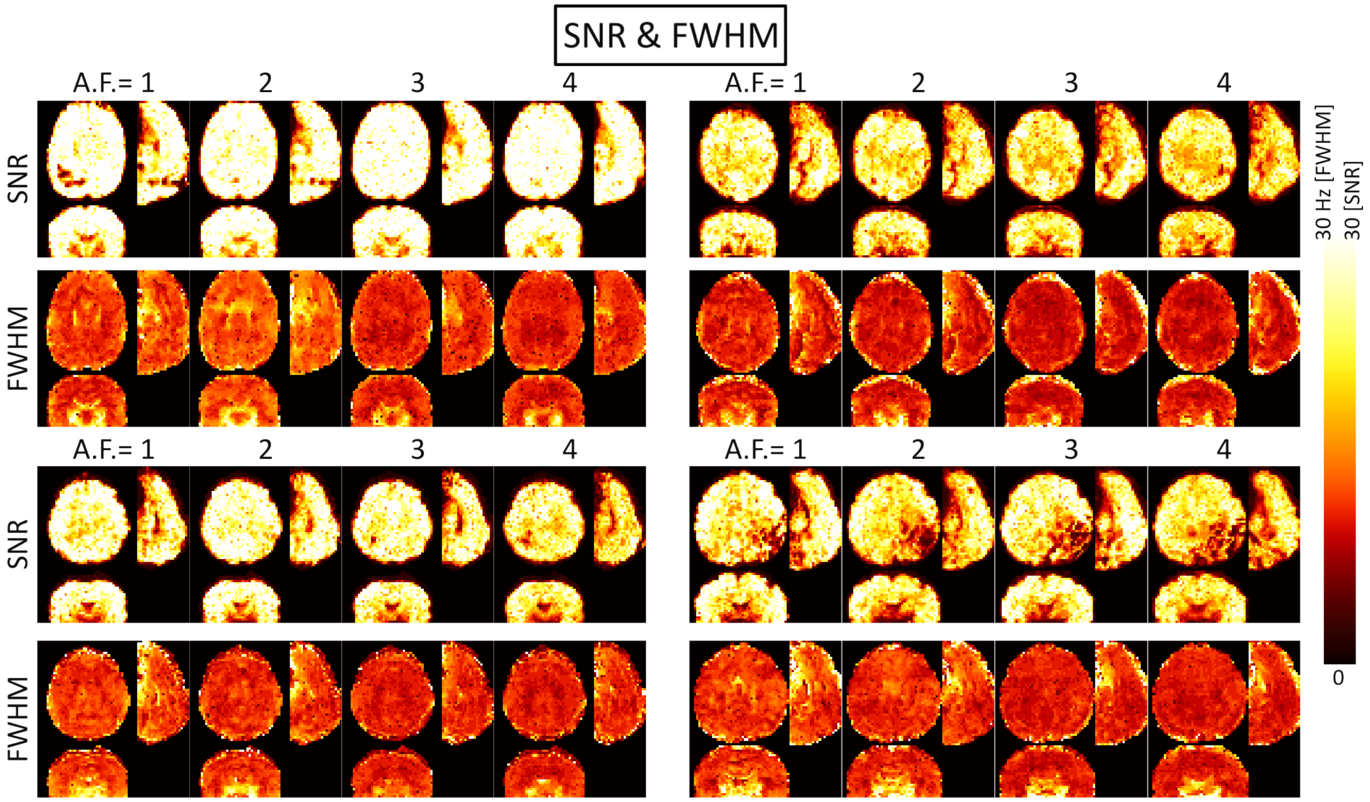

FIG. S7: Signal-to-Noise ratio (SNR) and spectral line-width (full width at half maximum, FWHM) maps obtained from four consecutive measurements with increasing acceleration factors (A.F.) using 3D ECCENTRIC ^{1}H-FID-MRSI on four healthy volunteers. The color scale ranges from 0 to 30 Hz for FWHM and 0 to 30 for SNR.

B. Alternative model reconstruction

In Fig.S8, 3D ECCENTRIC ^{1}H-FID-MRSI AF=2 from volunteer 1 is reconstructed with several alternative models. Following the notation of the of the main manuscript, we describe the models are defined as follows

SENSE	$\arg\min_{\rho,\mathbf{L}} \left\Vert \mathcal{W}\left(\mathbf{s} - \mathcal{FCB}\rho\right)\right\Vert_2^2$
SENSE-LR	$\arg\min_{\mathbf{U},\mathbf{V},\mathbf{L}} \left\Vert \mathcal{W}\left(\mathbf{s} - \mathcal{FCB}\left(\mathbf{UV}+\mathbf{L}\right)\right)\right\Vert_2^2$
CS-SENSE	$\arg\min_{\rho,\mathbf{L}} \left\Vert \mathcal{W}\left(\mathbf{s} - \mathcal{FCB}\left(\rho+\mathbf{L}\right)\right)\right\Vert_2^2 + \lambda \sum_{t=1}^{T} \mathrm{TGV}^2\{\rho(\mathbf{r},t)\}$
CS-SENSE-LR	$\arg\min_{\mathbf{U},\mathbf{V},\mathbf{L}} \left\Vert \mathcal{W}\left(\mathbf{s} - \mathcal{FCB}\left(\mathbf{UV}+\mathbf{L}\right)\right)\right\Vert_2^2 + \lambda \sum_{c=1}^{K} \mathrm{TGV}^2\{U_c\}$

The reconstruction model employed in the main manuscript, CS-SENSE-LR, demonstrates superior denoising capabilities for both metabolite maps and higher signal-to-noise ratio (SNR) maps, as illustrated in Figure S8. Notably, the full width at half maximum (FWHM) maps exhibit similar results for both CS-SENSE-LR and CS-SENSE. However, SENSE and SENSE-LR exhibit suboptimal performance for the reconstruction of the 3D ECCENTRIC MRSI data. These findings align with previous results obtained using a similar reconstruction model for Cartesian FID-MRSI reconstruction (Supplementary material in [3]).

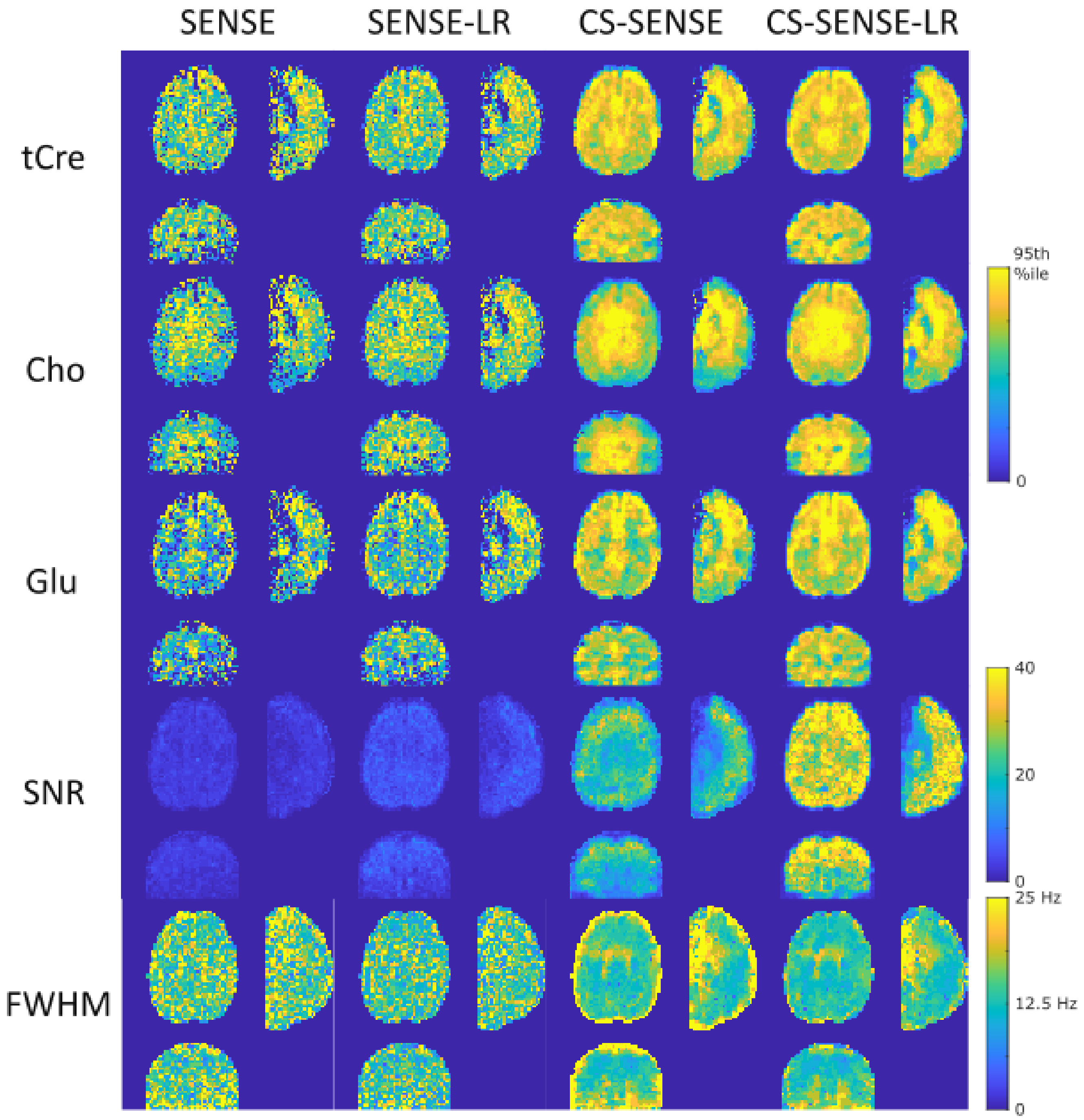

FIG. S8: Reconstruction of a 3D ECCENTRIC ^{1}H-FID-MRSI AF=2 dataset with several alternative models. (see text for detailed description)

C. 3D ECCENTRIC ^{1}H-FID-MRSI reconstruction with varying model rank, K

The model rank, denoted as K (main manuscript eq.(1)), was empirically determined through the following procedure. Initially, the components **U** and **V** were estimated using Singular Value Decomposition performed on the adjoint solution of the MRSI data, as outlined in the algorithm above. Subsequently, these initial components were visually inspected, and K was selected to ensure that components V_c for $c > K$ predominantly contained noise. An illustration of these initial spectral components is provided in fig.S11, wherein components beyond 30 are primarily

noise. This upper limit was consistently found to be approximately 35 across all subjects, and for safety, it was set to $K = 40$. Additionally, as depicted in fig.S9, when K falls below 20, key metabolite features begin to diminish, although the effect remains subtle. Notably, no significant disparity was observed in the resulting metabolite maps when K varied between 30 and 60. Regarding spectral quality parameters, the SNR consistently decreased with increasing K, while the FWHM remained unaffected. Fig.S10 shows the normalized root mean square errors (RMSE) of the metabolite maps reconstructed with rank $K = 20, \dots, 50$ relative to the metabolite maps with rank $K = 60$ are shown. The RMSE significantly decreases from $K = 10$ to 20, with only marginal improvements observed above $K = 30$. Consequently, selecting $K = 40$ was deemed a safe compromise, optimizing SNR while ensuring robustness.

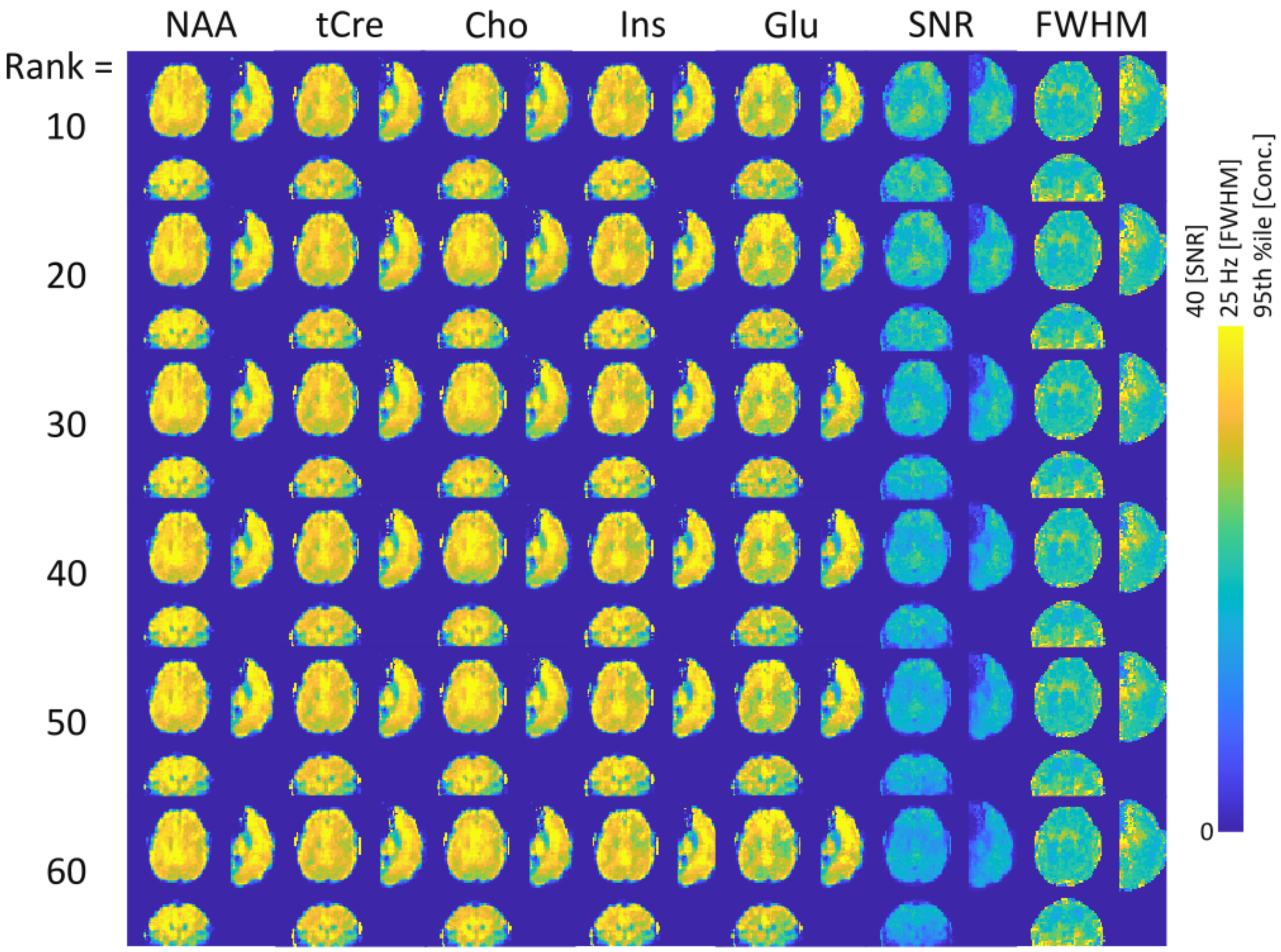

FIG. S9: Reconstruction of a 3D ECCENTRIC ^{1}H-FID-MRSI AF=2 dataset with varying model rank, K

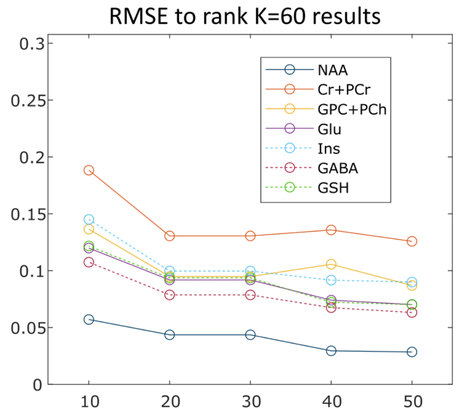

FIG. S10: Normalized root mean square error (RMSE) of metabolite maps reconstructed with specified rank K (x-axis). The error is calculated relative to the metabolite maps reconstructed with rank $K = 60$.

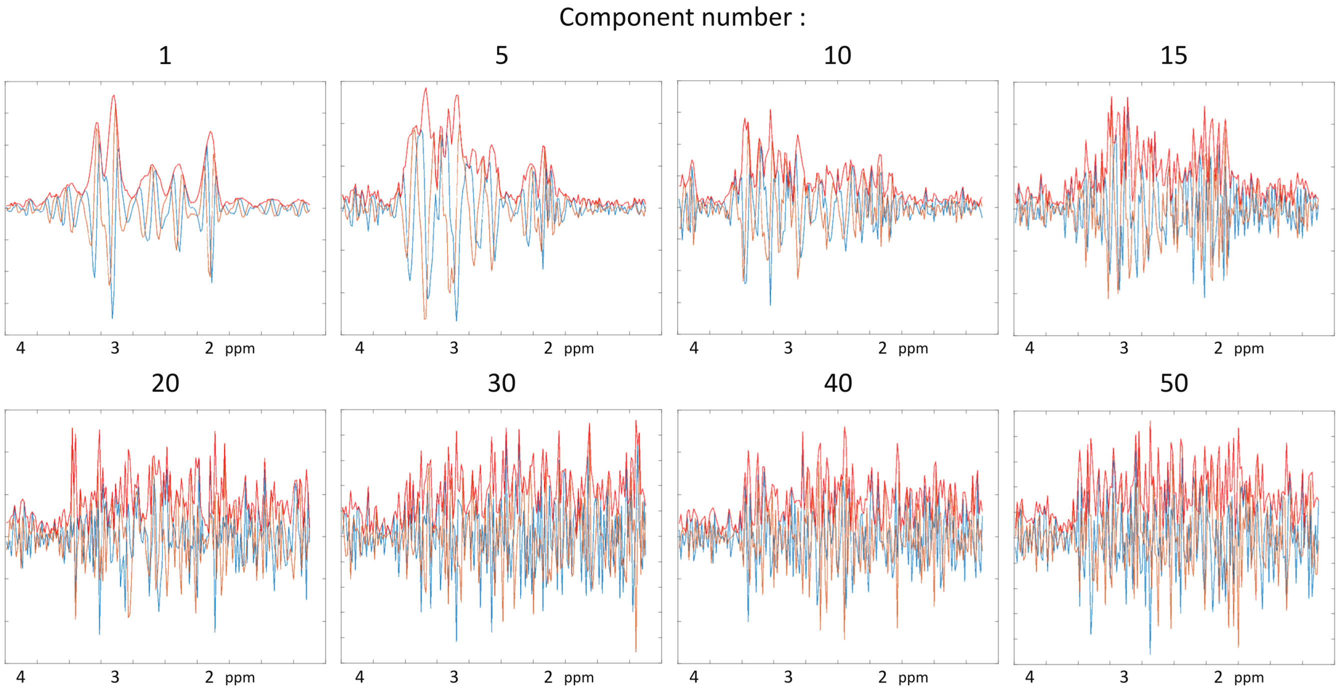

FIG. S11: Initial spectral components of the low-rank approximation for the 3D ECCENTRIC FID-MRSI AF=2 dataset. Blue represents real values, orange represents imaginary values, and red indicates the absolute value.

D. Comparative analysis of high-resolution and low-resolution water as reference signals

Here, we investigate the impact on MRSI results when using a rapid water reference data acquired either with low-resolution rosette parameters ($9.5 \times 9.5 \times 5.5$ mm^3, $23 \times 23 \times 19$ matrix, TA=1 min 16 sec) or acquired with identical parameters to the metabolite acquisition ($3.4 \times 3.4 \times 3.4$ mm^3, $64 \times 64 \times 31$ matrix, TA=9 min 20 sec) but with the water suppression RF turned off. The results are presented in fig.S12. Although the difference in acquisition resolution is visible around the skull in the absolute signal images, there is no significant effect observed over the coil sensitivity maps estimated when using either reference dataset nor for the resulting metabolite mapping. Due to the absence of T_1 contrast in the brain, the signal profile remains smooth and primarily reflects B_1 variations throughout the cerebral tissue, for which a low-resolution acquisition is sufficient.

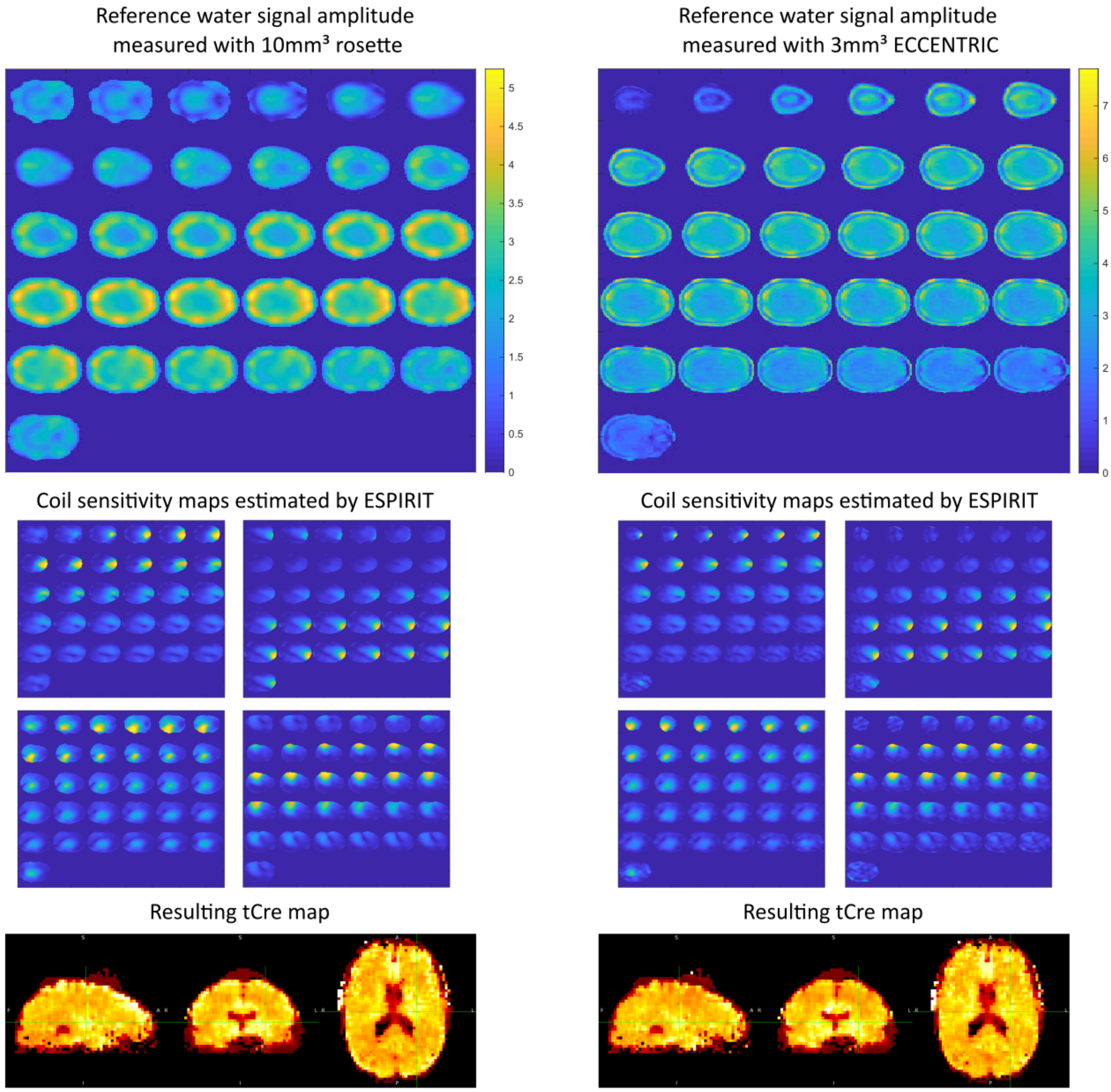

FIG. S12: Top, reference water signal acquired with either low-resolution rosette parameters ($9.5 \times 9.5 \times 5.5$ mm^3, $23 \times 23 \times 19$ matrix) or parameters identical to the metabolite acquisition ($3.4 \times 3.4 \times 3.4$ mm^3, $64 \times 64 \times 31$ matrix). Center, 4 out of 32 coil sensitivity profiles estimated from the reference water signal. Bottom, the total creatine (tCre) maps quantified using either the low resolution (left) or the high resolution (right) water reference signal. Both maps are shown on the same I.U. scale

E. ECCENTRIC water imaging in healthy volunteers

To investigate the ability of ECCENTRIC to image brain structure, we performed water imaging in several healthy volunteers using 3D FID-MRSI ECCENTRIC. The water suppression was turned off, and we used the 3.4 mm protocol with larger FA $= 40°$ and shorter TR $= 100$ ms to produce T_1 weighed images. ECCENTRIC k-space data were acquired fully sampled (AF=1) and the acceleration was obtained by retrospective CS undersampling in post-processing. Results in Fig. S13 show that fully sampled ECCENTRIC images reveal similar brain structure as T_1-weighted GRE images acquired with matched spatial resolution and tissue contrast. Considering the fully sampled

(AF=1) ECCENTRIC as ground truth, the SSIM $\geq$ 0.99 and correlation factor $\geq$ 0.92 for images obtained with AF = 1-12. Visually, almost no difference can be observed between images obtained with AF = 1-4. For AF $\geq$ 8 the noise level increases, which interferes with fine structural details.

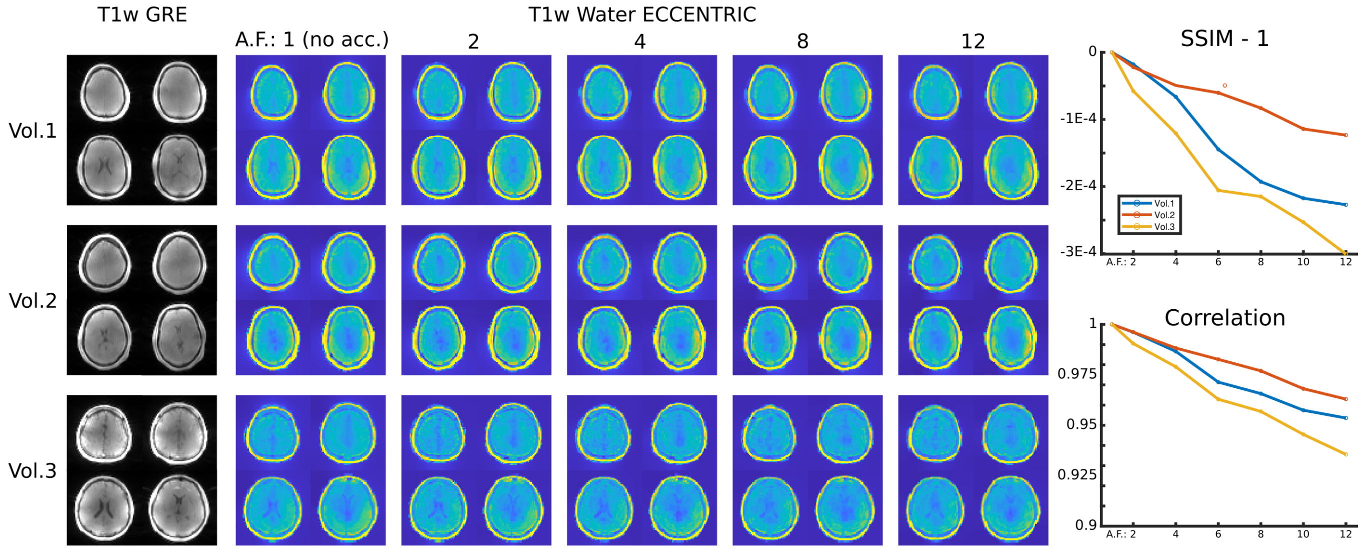

FIG. S13: Water imaging of human brain in healthy volunteers using 3D-ECCENTRIC with 3.4mm isotropic voxel size. Left, images obtained by T_1-weighted 3D GRE acquired with matched FA, TR, TE and spatial resolution. Middle, water images obtained with 3D-ECCENTRIC for acceleration factors 1-12. Right, SSIM and correlation factors for accelerated ECCENTRIC water images are calculated considering the fully sampled image (AF=1) as ground truth. Four slices are shown for each volunteer.

III. OPTIMIZING ECCENTRIC FOR HIGH SNR AND ACCELERATED HIGH-RESOLUTION MRSI

By design, the k-space acquisition by ECCENTRIC is characterized by: the circle radius (CR), compress sensing acceleration factor (AF), image matrix size (MS) and field-of-view (FoV). In addition, acquisition of the time dimension (FID) for spectroscopy is characterized by the spectral bandwidth (SW), the dwell-time, and the number of time points. In particular, for spectral-spatial encoding (SSE) there is a dependency between the spectral bandwidth, field-of-view and image resolution. Compared to other SSE schemes, ECCENTRIC allows very high flexibility in the choice of SW, FoV and MS, which is particularly needed at ultra-high field (7T and beyond) and to operate within the technical limits of the gradient system minimizing electrical, mechanical and thermal stress.

Importantly, the ECCENTRIC flexibility can be used to optimize the SNR and acquisition time while pushing the image resolution. ECCENTRIC parameters have different impact on the measured signal-to-noise ratio (SNR) and acquisition time (TA) as highlighted in the following table:

Change \ Effect	SNR	TA
Circle Radius $\nearrow$	$\searrow$	$\searrow$
CS Acceleration $\nearrow$	$\rightarrow$ (image smoothness $\nearrow$)	$\searrow$
Matrix Size $\nearrow$	$\searrow$	$\nearrow$

TABLE S1:

3D ECCENTRIC MRSI can be optimized by reducing CR and thus increasing the sampling density in k-space, which can be designed to sample more the center of k-space to increase SNR. In addition, the CR reduction allows a large range of spectral windows by controlling the gradient slew rate as needed based on the image resolution. However, the reduction in CR requires a higher CS acceleration for an equivalent acquisition time. As we showed, CS acceleration up to 4 provides high quality metabolite images, and this can be traded to optimize SNR with smaller CR.

To explore the flexibility of ECCENTRIC parameters for SNR optimization, three acquisitions with the same isotropic resolution (5mm) and acquisition time (14min) were acquired with different ECCENTRIC circle radii (CR)

and CS acceleration factors: 1) CR = kmax/4 and AF = 1; 2) CR = kmax/8 and AF = 2; 3) CR = kmax/16 and AF = 4. Sampling patterns of the k-space are shown in Fig. S14.

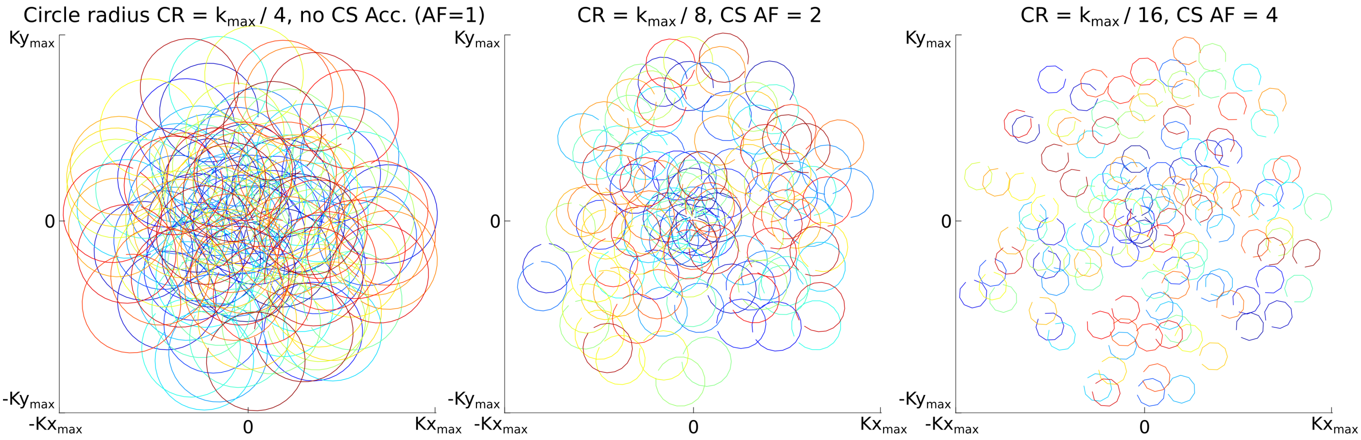

FIG. S14: ECCENTRIC k-space sampling for various circle radii (CR) and compress sensing acceleration (AF).

Metabolite maps obtained in a healthy volunteer are presented in Fig. S15. Higher SNR can be noticed for the six metabolites as the circle radii is decreased, while only minor blurring is apparent at the highest acceleration.

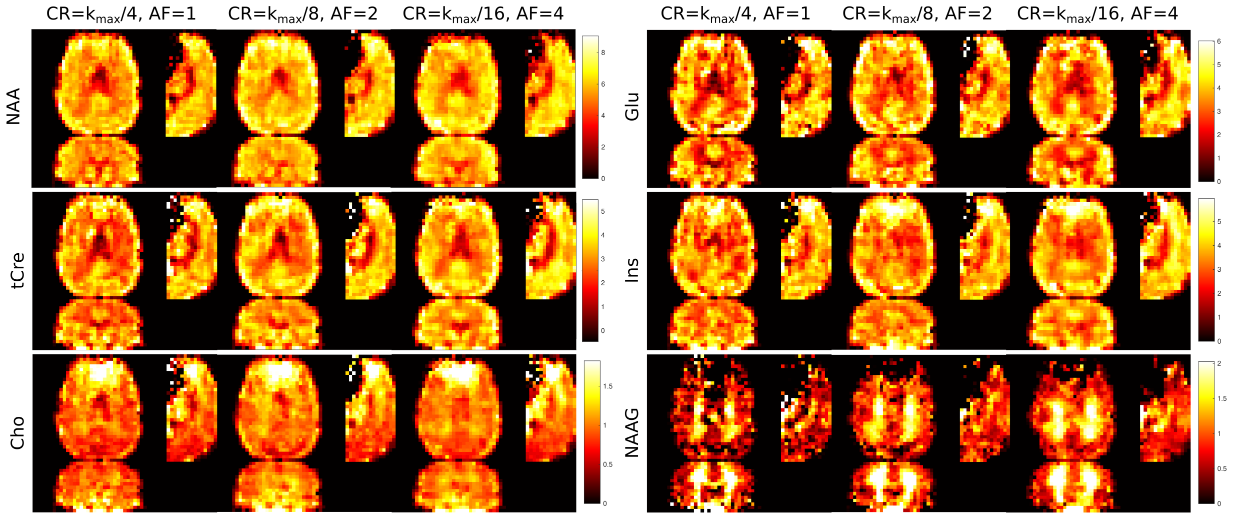

FIG. S15: Metabolite maps of NAA, total Creatine, total Choline, Glu, Ins and NAAG produced from 3D ^{1}H-FID-MRSI ECCENTRIC acquisitions at 5 mm isotropic image resolution in 14 min with various circle radii CR and compress sensing AF.

Quantitative analysis in Fig. S16 shows that decreasing CR results in a notable gain in metabolite SNR of + 30% for kmax/8 and + 40% for kmax/16 relative to kmax/4, respectively. In the same time, the linewidth is stable across the different protocols. The increase in SNR enables more precise metabolite quantification resulting in lower CRLB for spectral fitting, especially for the low signal metabolites. Among the 3 protocols, the protocol with CR = kmax/8 and AF = 2 showed the best performance with a marked increase in SNR and little visible blurring on metabolite maps. These results demonstrate that CR and AF allow SNR optimization of 3D ECCENTRIC MRSI for a desired image resolution and acquisition time.

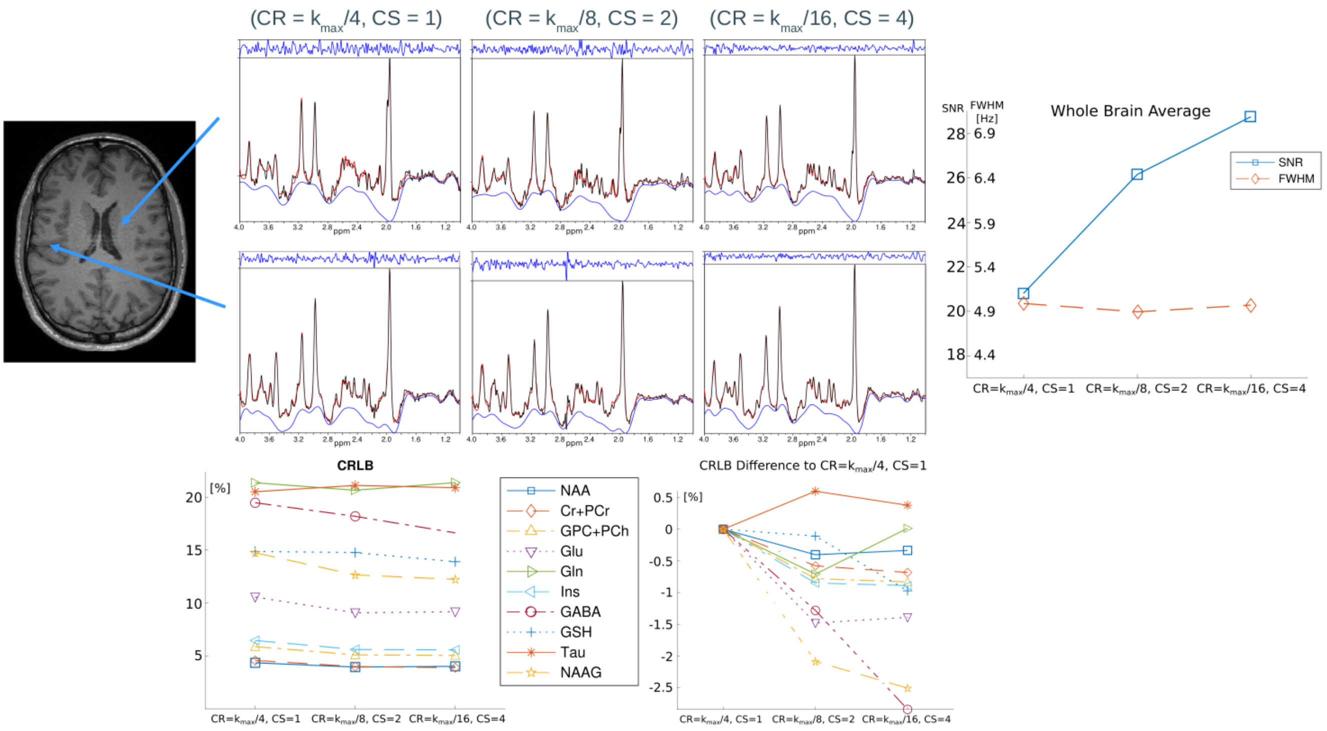

FIG. S16: Quantitative analysis of 3D ^{1}H-FID-MRSI ECCENTRIC acquisitions with various circle radius CR and compress sensing AF.

[1] F. Knoll, K. Bredies, T. Pock, and R. Stollberger, Magnetic Resonance in Medicine **65**, 480 (2011), ISSN 07403194.

[2] B. Bilgic, I. Chatnuntawech, A. P. Fan, K. Setsompop, S. F. Cauley, L. L. Wald, and E. Adalsteinsson, J Magn Reson Imaging **40**, 181 (2014), ISSN 15222586, NIHMS150003.

[3] A. Klauser, B. Strasser, B. Thapa, F. Lazeyras, and O. Andronesi, Journal of Magnetic Resonance **331** (2021), ISSN 10960856.
